# Pre-existing adipose tissue signaling profile related to obesity determines disease outcome of COVID-19: addressing obesity should be a priority for future pandemic preparedness

**DOI:** 10.3389/fendo.2025.1506065

**Published:** 2025-04-25

**Authors:** Arifa Parker, Kelly Petersen-Ross, Tongai Maponga, Samina Parkar, Nadiya Ahmed, Candice I. Snyders, Martin Kidd, Jantjie J. Taljaard, Graeme Meintjes, Coenraad F. N. Koegelenberg, Léanie Kleynhans, Carine Smith

**Affiliations:** ^1^ Division of General Medicine, Department of Medicine, Faculty of Medicine and Health Sciences, Stellenbosch University and Tygerberg Hospital, Cape Town, South Africa; ^2^ Experimental Medicine Unit, Department of Medicine, Faculty of Medicine and Health Sciences, Stellenbosch University and Tygerberg Hospital, Cape Town, South Africa; ^3^ Division of Virology, Department of Pathology, Faculty of Medicine and Health Sciences, Stellenbosch University and Tygerberg Hospital, Cape Town, South Africa; ^4^ Division of Dermatology, Department of Medicine, Faculty of Medicine and Health Sciences, Stellenbosch University and Tygerberg Hospital, Cape Town, South Africa; ^5^ Division of General Surgery, Department of Surgery, Faculty of Medicine and Health Sciences, Stellenbosch University and Tygerberg Hospital, Cape Town, South Africa; ^6^ Department of Science and Innovation - National Research Foundation Centre of Excellence for Biomedical Tuberculosis Research, South African Medical Research Council Centre for Tuberculosis Research, Division of Molecular Biology and Human Genetics, Department of Biomedical Sciences, Faculty of Medicine and Health Sciences, Stellenbosch University, Cape Town, South Africa; ^7^ Centre for Statistical Consultation, Department of Statistics and Actuarial Sciences, Stellenbosch University, Cape Town, South Africa; ^8^ Division of Infectious Diseases, Department of Medicine, Faculty of Medicine and Health Sciences, Stellenbosch University and Tygerberg Hospital, Cape Town, South Africa; ^9^ Department of Medicine, University of Cape Town and Groote Schuur Hospital, Cape Town, South Africa; ^10^ Wellcome Centre for Infectious Diseases Research in Africa, Institute of Infectious Disease and Molecular Medicine, University of Cape Town, Cape Town, South Africa; ^11^ Division of Pulmonology, Department of Medicine, Faculty of Medicine and Health Sciences, Stellenbosch University and Tygerberg Hospital, Cape Town, South Africa; ^12^ Mater Research Institute - The University of Queensland, Translational Research Institute, Brisbane, QLD, Australia

**Keywords:** COVID-19, adipose tissue, leptin, resistin, adiponectin, adipokine, obesity, HIV

## Abstract

**Objectives:**

Obesity is associated with COVID-19 severity and mortality. We investigated relationships between adipokines, cytokines and redox parameters with obesity, human immunodeficiency virus (HIV), severity and outcome.

**Methods:**

In the exploratory study, adipose tissue (AT) was sampled in patients with COVID-19 on admission. Concentrations of leptin, adiponectin, resistin, interleukin 1 beta (IL-1b), IL-2, IL-6, IL-10, IL-17, tumor necrosis factor alpha (TNF-a), monocyte chemoattractant protein 1 (MCP-1), Trolox equivalent antioxidant capacity (TEAC), oxidative stress (H_2_0_2_) and malonaldehyde (MDA) were determined.

**Results:**

Thirty-eight biopsies of subcutaneous adipose tissue were obtained (prevalence of HIV was 39% and of obesity 61%). Higher IL-6 serum concentrations (p=0.03) were associated with more severe COVID-19, and higher serum IL-10 concentrations, (p=0.03) with mortality. People with obesity had higher leptin concentrations (p=0.03, and p<0.01), lower adiponectin/leptin (p=0.03 and p<0.01), and higher leptin/resistin ratios (p=0.09 and p<0.01) in both AT and serum respectively. Higher leptin/resistin (p=0.04) and lower adiponectin/resistin (p=0.05) ratios in AT, but not serum, were predictive of mortality. HIV was not associated with any differences. Relationships between resistin and redox indicators, TEAC and MDA, suggest a dysregulation of metabolic vs immune-relevant effect of resistin, which differentially predicted severity and mortality. SARS-CoV-2 RNA was detected in the subcutaneous AT in 3/8 patients who demised, but only in 1/30 who survived.

**Conclusion:**

Given the significant link demonstrated between leptin dysregulation in obesity and mortal severity of COVID-19, addressing obesity should be a priority therapeutic target in terms of future pandemic preparedness. Mechanistic studies are recommended to further elucidate the importance of metabolic vs immune modulation by resistin in COVID-19, to identify future therapeutic targets.

## Introduction

The Coronavirus disease 2019 (COVID-19) pandemic highlighted the pleiotropic effects of adipose tissue (AT), and its link with infection and inflammation (1). AT is comprised of adipocytes and the stromal vascular fraction, made up of fibroblasts, endothelium, blood, macrophages, and other immune cells (2). In addition to its traditional function of energy storage, AT also has endocrine and immune properties (2). Both the adipocytes and macrophages in AT secrete pro- and anti-inflammatory cytokines which contribute to the overwhelming inflammatory response or ‘cytokine storm’ reported in patients hospitalized with severe COVID-19 (1, 2). Although the pandemic of COVID-19 is no longer an imminent global threat, much can be learned from the COVID-19 literature, in terms of measures to be put in place for future pandemic preparedness. This includes both management of patients during a pandemic, as well as management of the risk for co-morbid sequelae in the patient with obesity.

For example, obesity has been established as a significant risk factor for severe COVID-19 and mortality (3, 4). Obesity is associated with underlying chronic low grade localized AT inflammation, which may prime the immune system for an exaggerated inflammatory response to novel pathogens such as SARS-CoV-2 (5). Furthermore, severe acute respiratory syndrome coronavirus 2 (SARS-CoV-2) has been demonstrated in various extra-pulmonary sites, including AT, where it has been detected in both adipocytes and AT macrophages in post-mortem samples (6–8). The membrane protein angiotensin converting enzyme 2 (ACE-2) receptors are abundantly expressed in adipose tissue (9), which SARS-CoV-2 (as well as other similar viruses) can bind to facilitating cell entry and viral replication (5). In persons with obesity, adipocytes are hypertrophied and consequently have increased expression of ACE-2 receptors per cell (10), which along with impaired viral responses in obesity, may result in a higher intracellular SARS-CoV-2 load or reservoir in AT (11). In addition to these avenues by which obesity may affect prognosis in COVID-19, a preclinical report of longer-term adverse mitochondrial functionality (12), raises concerns of post-recovery susceptibility to chronic diseases in the obese patient – especially since obesity is already linked to unfavorable redox status.

In terms of future therapeutic targets, it is important to note that many biomarkers or outcome predictors in COVID-19, are also associated with obesity even in the absence of infection. Most prominently, leptin – known to be elevated in people with obesity (13) - was reported to recruit AT and alveolar macrophages and to induce secretion of interleukin (IL)-1, tumor necrosis factor alpha (TNF-a) and IL-6 (i.e. the cytokine storm), in COVID-19 disease (5) and thus contributed significantly to a pro-inflammatory outcome (5, 14, 15). Of interest, hyperleptinemia contributes to lung inflammation and is also linked to viral infections such as influenza (16), which underlined the broader applicability of COVID disease data to other viral threats. Furthermore, increased resistin, another hallmark of obesity (17) and pro-inflammatory action (18–20), was associated with increased IL-6 and TNF-a signaling, activation of complement, and the synthesis of TNF-a, IL-6 and monocyte chemoattractant protein 1 (MCP-1) in COVID disease (18, 19). By generating reactive oxygen species, resistin contributes to oxidative stress in various tissues, including the lung (20). Adiponectin, pathologically reduced in people with obesity (PWO) and by leptin, IL-6, and TNF-a (14), activates COVID-19-linked secretion of the anti-inflammatory cytokine, IL-10, and reduces secretion of IL-6 and TNF-a (15, 21).

Of further interest, although strong correlations have been reported between *serum* cytokine levels and redox parameters in a COVID-19 patient cohort (22), lack of a AT profile in parallel to this data, precludes conclusions on the contribution of obesity in the context of redox and the cytokine storm in COVID disease. An unbalanced redox status in the AT would play an important role in exacerbating a pro-inflammatory microenvironment which may contribute to progression or worse prognosis in conditions such as COVID-19, as well as exacerbating longer-term susceptibility to post-covid health issues resulting from chronic inflammation.

Therefore, the aim of the current study was to investigate the relationship between AT redox status and AT signaling profile vs prognosis in a condition such as COVID-19, to shed light on the importance of management of obesity during viral infection, as well as in preparation for future pandemic scenarios.

## Materials and methods

### Study design, setting, and population

We conducted a single center exploratory cross-sectional study investigating the sAT profile of patients admitted to the COVID-19 medical wards with COVID-19 pneumonia. The study was performed at Tygerberg Hospital, a university affiliated tertiary hospital in Cape Town, South Africa. The hospital is the largest in the Western Cape province and serves a population with a high burden of communicable (HIV and tuberculosis) and non-communicable diseases. Patient recruitment for this study occurred from 14 July 2021 until 08 September 2021, which coincided with the third wave of the pandemic in South Africa, when the Delta variant of SARS-CoV-2 predominated.

### Participant selection and ethical considerations

Ethical approval for this study was obtained from the Health Research Ethics Committee of Stellenbosch University (HREC Reference number: N20/04/002_COVID-19). As this was an exploratory study investigating the relationship between adipose tissue and COVID-19, we aimed to recruit a minimum of 20 to 30 patients. The samples size was determined by feasibility rather than statistical calculations. A total number of 38 patients were recruited before the COVID-19 wave ended and there were no further hospital admissions meeting the inclusion criteria. Written informed consent was obtained from all participants. All patients had to fulfill the following inclusion criteria: (1) be at least 18 years of age, (2) have a positive nasopharyngeal SARS-CoV-2 polymerase chain reaction (PCR) or antigen test, (3) be hospitalized with COVID-19 pneumonia requiring oxygen, (4) be within 48 hours of admission, and (5) should not have received > 2 doses of dexamethasone 6mg daily, or prednisone 40mg daily. Patients were excluded based on presence of any malignancy, autoimmune or connective tissue disease, or diabetic keto-acidosis, as well as any patient in ICU or who was intubated, incidental SARS-CoV-2 positive test (PCR or antigen) without a pneumonia, or inability or being unwilling to provide informed consent.

### Clinical and routine laboratory data collection

Clinical data collected included age, sex, symptoms (dyspnea, cough and/or fever), co-morbidities such as hypertension, diabetes, obesity, HIV status and tuberculosis, as well as an assessment of COVID-19 severity. A clinical definition for early stage severe COVID-19 included patients with symptoms (dyspnea, cough and/or fever) and minimal chest radiograph infiltrates, who required minimal supplementary oxygen (nasal prong or 40% facemask oxygen). Advanced stage severe COVID-19 was clinically defined as those with symptoms and significant chest radiograph infiltrates, who required non-rebreather mask oxygen or non-invasive ventilation in a medical ward setting. Obesity was defined as a body mass index (BMI) of ≥ 30 kg/m^2^. Duration of hospital stay, and outcome (survival to discharge or death in hospital) was recorded. Laboratory data generated as part of routine care was collected and included admission full blood counts, differential leukocyte counts, as well as concentrations of creatinine, general inflammatory markers (C reactive protein, lactate dehydrogenase), and the metabolic indicators glycated hemoglobin (HbA1C), vitamin D and cholesterol.

### AT and serum sampling and processing

AT samples were obtained between 10h00 and 12h00 to minimize any effect of diurnal variation in adipokine concentrations. A biopsy of abdominal sAT (akin to a skin biopsy) was collected from consenting participants by first anaesthetizing the skin after cleaning the area with chlorhexidine. A 6mm superficial skin incision was made just below the umbilicus and two 6mm punches of sAT were obtained. After the procedure the biopsy site was covered and cared for by standard procedures. None of the 38 patients required sutures or had complications after the adipose tissue biopsy procedure. Biopsies were immediately flash frozen in liquid nitrogen and stored at -80°C until analysis. On the day of analysis, biopsies were thawed on ice. AT biopsies were mechanically homogenized in 250uL standard PBS (pH 7.2) using the Omni bead ruptor (Sigma Aldrich, USA). Thereafter the homogenates were centrifuged at 10,000xg for 15 minutes at 4°C to obtain the supernatants used for the analyses. The sAT homogenate supernatant obtained was immediately analyzed to determine adipokine concentrations. The lipid rich (pellet) layer of homogenate was removed via scraping of tubes and stored at -80°C. This was used to extract RNA for detection of SARS-CoV-2. Total protein content of each sAT homogenate sample was determined by using a Jenway 7415 nanodrop Micro-volume Spectrophotometer (Cole-Parmer Instrument Company, LLC, United Kingdom). The concentrations of analytes in sAT were expressed relative to sample protein content.

Blood for cytokine analysis was collected into serum separation tubes (Vacutainer, BD Systems, Plymouth, UK) within 15 minutes of collection of sAT biopsies. Blood samples were allowed to clot at room temperature for 10 minutes and then centrifuged at 1,300xg for 10 minutes, within 1 hour of samples collection. The serum was aliquoted and stored at -80°C for batch analysis.

### AT and serum cytokine and chemokine signaling profile

Concentrations of the following cytokines and adipokines were measured using Discovery Luminex Assays from R&D Systems (LXSAHM; R&D Systems, Inc., MN, USA): adiponectin, leptin, resistin, IL-1b, IL-2, IL-6, TNF-a, interferon gamma (IFN-g), IL-17, IL-10, and MCP-1. All analytes were measured simultaneously in sAT samples (diluted 1:2) using an 11-plex assay. Two kits were used for serum samples: a 1-plex to measure adiponectin (sample diluted 1:200) and a 10-plex (sample diluted 1:2) to measure the remaining analytes. Assays were performed following the manufacturer’s instructions and the plates read on the Luminex LX200 Instrument with xPONENT software.

### AT viral detection

RNA was extracted from 160µL of the homogenate using the NucleoSpin RNA Virus kit (Macherey-Nagel, Germany). Samples were eluted into 50µL of elution buffer. Testing for SARS-CoV-2 RNA through detection of the E gene was performed using reverse-transcriptase real-time PCR utilizing the SARS-CoV-2 ModularDx kit (TIB MOLBIOL, Germany) on the CFX96 Touch real-time PCR system (Bio-Rad, USA). The SARS-CoV-2 ModularDx kit can further differentiate SARS-CoV-2 sequences if they belong to the Delta or other variants by detecting the deletion mutation at positions 157/158 of the Delta within the spike region.

### AT redox and signaling profile

#### Hydrogen peroxide assay

Free radical estimation was conducted using a commercial Hydrogen Peroxide (H_2_O_2_) colorimetric assay kit (Elabscience, EBC-K102-S, USA) with minor modifications. Adipose tissue homogenates were centrifuged at 10,000xg at 4°C for 10 minutes and supernatants were collected for the assay. Briefly, 100ul of the buffer solution 1 was added to a 96 well plate and incubated for 10 minutes at 37°C. Thereafter, 100ul of reagent 2 was added along with either 10ul of H_2_O_2_ standard or 20ul of sAT supernatant. The 96 well plate was then read at 405nm on a microplate reader (Victor Nivo Multimode Plate Reader, Perkin Elmer).

#### Trolox equivalent antioxidant capacity (TEAC) assay

Total antioxidant capacity was measured using the TEAC assay adapted from Miller, 1993. The radical 2,2’-Azino-bis(3-ethylbenzothiazoline-6-sulfonic acid) (Sigma, A1888) was combined with Potassium-peroxodisulphate (Merck,105091) to develop overnight. In a 96 well plate the sample was added to the ABTS mix and incubated at room temperature for 30 minutes. The plate was then read at 734nm on the Victor Nivo Multimode Plate Reader, Perkin Elmer.

#### Thiobarbituric acid reactive substances (TBARS) assay

Oxidative damage was measured using TBARS assay which measures the lipid peroxidation product, malondialdehyde (MDA) in a sample. The method used was adapted from Varshney and Kale (1990). Briefly, butanol and saturated sodium chloride are added to the MDA-TBA complex formed in a sample. Absorbance was read at 532nm (with a reference wavelength of 572nm) using a Victor Nivo Multimode Plate Reader, Perkin Elmer.

### Statistical analysis

Clinical parameters were compared between BMI groups and outcome groups (alive or died) using cross tabulation and the Fisher Exact test for categorical data, and one-way ANOVA for continuous data. Immune marker values that were too low to be extrapolated by the xPONENT Software, were imputed to the lowest value that could be extrapolated by the software minus 10% of the range. For adiponectin concentrations in sAT > 20% of values were too high to be extrapolated by the xPONENT Software so the fluorescence intensity (FI) was used as a surrogate. Corresponding FI of leptin and resistin were used to calculate adipokine ratios in sAT. Serum IFN-g was not included in analysis as > 20% of both observed concentrations and fluorescence intensities were too low to be extrapolated by the software. Immune marker concentrations were compared between above mentioned groups using one-way ANOVA. Homogeneity-of-variance was checked using the Levene’s test and in cases where it did not hold, the Welch test was done. However, in all cases the Welch test gave similar results to the ANOVA results. Normality of marker concentrations were first checked by visually inspecting normal probability plots, and in cases where outliers were detected, winsorizing was done by calculating interquartile range (IQR) scores using the R package “outliers”. Values outside the IQR ranges were changed to fall on the borders of the IQR ranges, thus still retaining the rank of the data points (reducing their influence on the mean) and leaving the non-outlier data points untouched. The Benjamini-Hochberg procedure was used to correct for multiple testing. The adjusted p-values did not meet the 5% level of significance; therefore, unadjusted p-values were reported. The effect of potential confounders (age, sex, obesity, hypertension, diabetes, HIV and severity) was assessed using a Factored ANOVA which includes each variable as a second main effect in the ANOVA (together with patient outcome). To understand the interplay between signaling role players, Spearman correlations were calculated between redox parameters and adipokines, with consideration of obesity, disease severity and outcome.

## Results

### Baseline clinical data and routine laboratory data

The mean (± standard deviation) age of participating patients was 51 ± 10 years, and most patients were female (n=29, 76%). None had a history of prior COVID-19 or had received any COVID-19 vaccines. The most common comorbidities were obesity (n=23, 61%), hypertension (n=23, 61%) and HIV (n=15, 39%) (**Table 1**). All patients had at least one symptom, commonly dyspnea (n=36, 95%), cough (n=32, 84%) and fever (n=17, 45%), with a median (range) duration of symptoms of 9 (2 to 10) days at the time of sampling. There were 11 patients with early stage severe COVID-19 (29%) and 27 with advanced stage severe COVID-19 (71%). In people with HIV (PWH), 12 had at least one other comorbidity (80%). The mean CD4 count was 428 ± 290 cells/ul and HIV viral loads were available and suppressed in 14 of 15 PWH (93%). Where antiretroviral therapy was documented, 9 were on dolutegravir-based regimens, and 2 were on atazanavir/ritonavir-based regimens.

**Table 1:** Baseline clinical and laboratory test findings of COVID-19 hospitalized patients.

	All patients (n=38)	BMI ≥ 30 kg/m^2^ (n=23)	BMI < 30 kg/m^2^ (n=15)	p value	Died (n=8)	Survived (n=30)	p value
Clinical Features							
Age* (years)	51 (10.1)	52	50	0.69	55	50	0.17
Sex, Female	29 (76)	21 (91)	8 (53)	0.02	7 (88)	22 (73)	0.65
Sex, Male	9 (24)	2 (9)	7 (47)		1 (12)	8 (27)	
Cough	32 (84)	21 (91)	11 (73)	0.19	6 (19)	26 (81)	0.59
Dyspnea	36 (95)	22 (96)	14 (93)	1.00	8 (75)	28 (93)	0.32
Fever	17 (45)	11 (48)	6 (40)	0.74	2 (25)	15 (50)	0.26
Obesity	23 (61)	–	–	–	6 (75)	17 (57)	0.33
Hypertension	23 (61)	15 (65)	8 (53)	0.51	6 (75)	17 (57)	0.33
HIV	15 (39)	6 (26)	9 (60)	0.05	3 (38)	12 (40)	1.00
Diabetes	10 (26)	7 (30)	3 (20)	0.71	3 (38)	7 (23)	0.41
Dyslipidemia	4 (11)	3 (13)	1 (7)	1.00	2 (50)	2 (50)	0.19
Tuberculosis	1 (3)	0	1 (7)	–	1 (100)	0	–
Early severe	11 (29)	8 (35)	3 (20)	0.47	0 (0)	11 (100)	0.19
Advanced severe	27 (71)	15 (65)	12 (80)		8 (30)	19 (70)	
Laboratory tests							
HbA1c* (%)	7.3 (2.3)	7.4 (2.3)	7.1 (2.5)	0.69	7.4 (2,2	7.3 (2.4)	0.91
Cholesterol* (mmol/L)	4.0 (0.9)	4.0	4.0		4.0 (0.9)	4.0 (0.9)	0.98
Vitamin D* (nmol/L)	37.7 (16.4)	33 (13)	45 (18)	0.02	38 (12)	38 (18)	0.95
CRP** (mg/L)	117 (35-412)	125 (45 - 260)	105 (35 - 412)		100 (35 - 260)	119 (45 - 412)	
LDH* (U/L)	511 (175)	595 (184)	428 (125)	0.02	438 (168)	530 (177)	0.30
P/F Ratio*	114 (51)	121 (51)	103 (51)	0.33	102 (49)	118 (52)	0.42

Data is expressed as n (%) or *mean (standard deviation) or **median (interquartile range). HIV, Human Immunodeficiency Virus; BMI, Body mass index; HbA1C, glycated hemoglobin; CRP, C reactive protein; LDH, Lactate dehydrogenase; P/F ratio, partial pressure of oxygen to fraction of inspired oxygen. Fisher Exact test for categorical data, and one-way ANOVA for continuous data were used to test for significance.

PWO had significantly higher serum LDH, although higher LDH did not seem to be a prognostic indicator for survival (**Table 1**). Although PWO exhibited lower vitamin D concentrations than non-obese patients, low vitamin D was similar in patients who survived and those who died.

### Obesity-associated differences in blood and AT adipokine profile is linked to disease outcome

There were no differences in serum and sAT cytokine and adipokine concentrations in PWH compared to people without HIV. Obesity was associated with higher leptin concentrations, lower adiponectin/leptin and higher leptin/resistin ratios in both serum and sAT (**Table 2**). However, adipose tissue (but not serum) adipokine dysregulation - higher leptin/resistin and lower adiponectin/resistin – was associated with mortality.

**Table 2:** Adipose tissue and serum adipokine concentrations and adipokine ratios stratified by obesity, COVID-19 severity, and outcome.

Adipose Tissue:	Obesity			COVID-19 severity			Outcome		
	BMI ≥ 30 kg/m^2^	BMI < 30 kg/m^2^	p	Advanced Severe	Early Severe	p	Died	Survived	p
Adiponectin*	1672 (1290)	1133 (1228)	0.21	1359 (1216)	1705 (1449)	0.46	1184 (1110)	1533 (1326)	0.5
Leptin*	**1683 (1477)**	**692 (963)**	**0.03**	1171 (1299)	1587 (1572)	0.40	1059 (999)	1354 (1467)	0.60
Resistin*	860 (1097)	635 (894)	0.51	775 (1088)	761 (857)	0.97	428 (749)	863 (1067)	0.29
Leptin/resistin*	5.6 (5.4)	2.8 (4.0)	0.09	5.0 (5.7)	3.2 (2.5)	0.34	**7.7 (7.2)**	**3.6 (4.0)**	**0.04**
Adiponectin/resistin*	17.2 (25.6)	17.9 (27.0)	0.94	19.2 (24.3)	17.0 (30.2)	0.81	**8.1 (8.5)**	**20.1 (28.2)**	**0.05**
Adiponectin/leptin*	**9.0 (14.42)**	**22.5 (21.8)**	**0.03**	16.5 (21.0)	9.0 (10.1)	0.27	19.0 (25.8)	13.1 (16.6)	0.44
Serum:									
Adiponectin (mg/ml)	10.04 (3.0)	10.08 (4.4)	0.97	10.1 (3.6)	10.0 (3.5)	0.95	10.5 (3.2)	10.0 (3.7)	0.67
Leptin (ug/ml)	**68 (52)**	**26 (25)**	**<0.01**	47 (48)	63 (49)	0.36	70 (71)	47 (40)	0.23
Resistin (ug/ml)	24 (7)	22 (10)	0.57	23 (9)	24 (7)	0.89	23 (8)	24 (10)	0.68
Leptin/resistin	**3 (2.2)**	**1.3 (1.1)**	**<0.01**	2.1 (1.9)	2.8 (2.2)	0.35	3.1 (2.6)	2.1 (1.8)	0.22
Adiponectin/resistin	460 (178)	558 (351)	0.26	514 (287)	462 (186)	0.58	415 (239)	495 (269)	0.85
Adiponectin/leptin*	**241 (214)**	**884 (956)**	**<0.01**	599 (792)	239 (160)	0.15	470 (657)	501 (708)	0.91

Data reflect the differences in the means [± standard deviations(SD)] or the *winsorised means (± SD) of the ratios or concentrations measured in picograms per milligrams (pg/mg) for adipose tissue and picograms per milliliter (pg/ml) for serum. mg/ml, milligram/milliliter; ug/ml, microgram/milliliter. One way ANOVA was used to test for significance.Bold values = p ≤ 0.05.

In line with COVID literature, higher serum IL-6 concentration was associated with advanced severe disease, while higher serum IL-10 also predicted mortality (**Table 3**). AT concentrations of inflammatory cytokines did not align with changes reflected in serum. However, all AT cytokine concentrations were very low, which may have resulted in suboptimal detection accuracy. The decision was therefore made to exclude this data from interpretation. Correcting for age, sex, obesity, hypertension, diabetes, HIV and severity did not change the interpretation of any of the findings reported here (**Supplementary Data Sheet 1**).

**Table 3:** Adipose tissue and serum cytokine concentrations stratified by obesity, COVID-19 severity, and outcome.

	Obesity			COVID-19 severity			Outcome		
Adipose Tissue:	BMI ≥ 30 kg/m^2^	BMI < 30 kg/m^2^	p	Advanced Severe	Early severe	p	Died	Survived	p
TNF-a*	0.4 (0.3)	0.3 (0.3)	0.18	0.3 (0.3)	0.4 (0.4)	0.35	0.2 (0.2)	0.4 (0.4)	0.18
IL-2	94 (76)	74 (95)	0.47	75 (66)	114 (115)	0.19	51 (39)	95 (90)	0.19
IL-1b	0.7 (0.6)	0.4 (0.5)	0.23	0.8 (0.7)	0.5 (0.5)	0.15	0.3 (0.2)	0.7 (0.6)	0.08
IL -6*	1.4 (1.2)	1.1 (1.2)	0.45	1.2 (1.1)	1.6 (1.5)	0.34	0.9 (0.7)	1.4 (1.3)	0.25
IL-17*	0.7 (0.5)	0.5 (0.5)	0.38	0.6 (0.4)	0.7 (0.6)	0.31	0.4 (0.4)	0.7 (0.5)	0.21
IL-10	1.1 (0.8)	0.8 (0.8)	0.33	0.8 (0.7)	1.3 (1.1)	0.16	0.7 (0.5)	1.1 (0.9)	0.27
MCP-1*	14.5 (12.6)	12.0 (11.9)	0.53	12.7 (11.6)	15.5 (14.0)	0.53	9.2 (8.4)	14.7 (12.9)	0.26
Serum:									
TNF-a	9.7 (5.3)	7.8 (3.8)	0.25	8.9 (4.7)	9.1 (5.1)	0.92	9.1 (3.6)	8.9 (5.1)	0.93
IL-2	149 (43)	140 (45)	0.55	142 (45)	153 (41)	0.51	145 (35)	145 (46)	0.97
IL-1b*	**5.5 (0.6)**	**5.2 (0.4)**	**0.03**	5.3 (0.6)	5.5 (0.5)	0.33	5.4 (0.7)	5.4 (0.5)	0.84
IL- 6	11.7 (11.3)	14.1 (13.2)	0.55	**15.3 (12.7)**	**6.2 (6.1)**	**0.03**	16.8 (13.4)	11.6 (11.6)	0.28
IL-17*	3.2 (2.8)	2.3 (2.4)	0.30	2.8 (2.7)	2.8 (2.8)	0.96	2.7 (2.9)	2.9 (2.7)	0.89
IL-10	5.1 (3.3)	5.0 (4.3)	0.94	5.5 (4.1)	3.9 (2.0)	0.23	**7.5 (5.2)**	**4.4 (2.9)**	**0.03**
MCP-1	408 (298)	580 (515)	0.20	516 (416)	378 (360)	0.34	540 (471)	459 (387)	0.52

Data reflect the differences in the means (± SD) or the *winsorised means (± SD) of cytokine concentrations, in pg/mg for adipose tissue and pg/ml for serum. One way ANOVA was used to test for significance.Bold values = p < 0.05.

### Antioxidant capacity of AT reflects changes in response and predicted disease outcome

**Figure 1:**
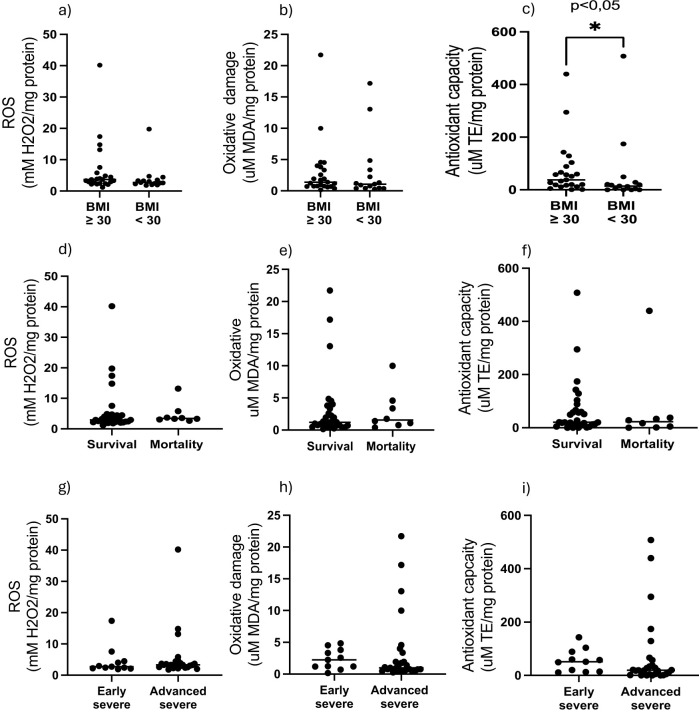
Adipose tissue homogenate levels of H_2_O_2_, MDA and Trolox equivalent antioxidant capacity stratified by **(a–c)** obesity, **(d–e)** outcome, **(g–i)** COVID-19 severity.

**Figure 2:**
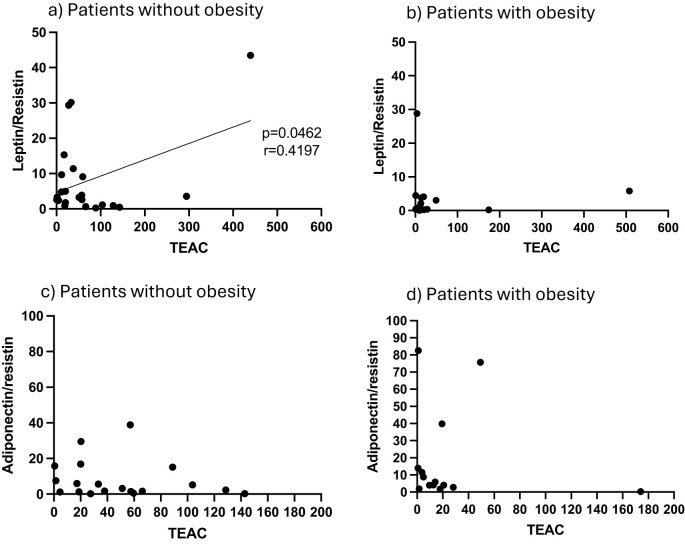
**(a)** Adipose tissue homogenate levels of Trolox equivalent antioxidant capacity (TEAC) in correlation to the ratio of leptin/resistin for patients classified as non-obese **(a)** and obese **(b)**, as well as the ratio of adiponectin/resistin in non-obese **(c)** and obese **(d)** patients.

**Figure 3:**
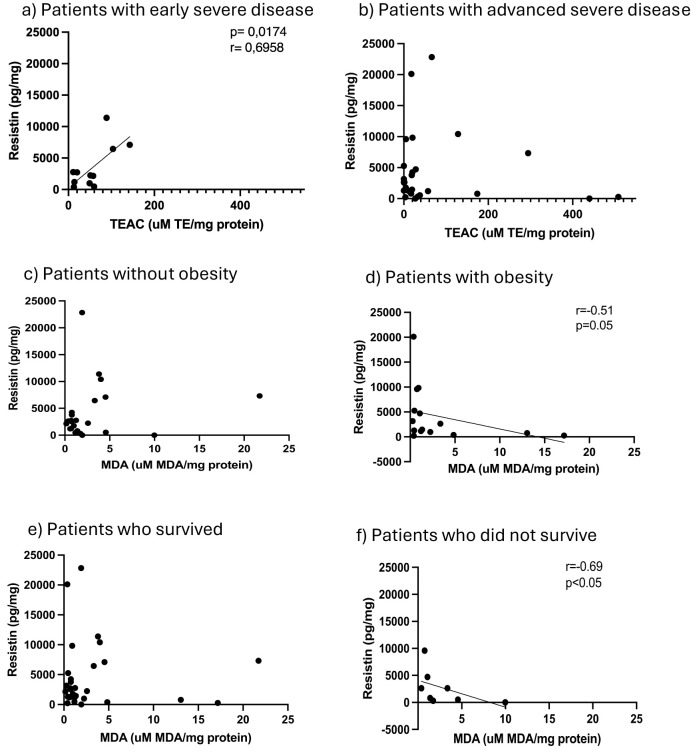
Adipose tissue homogenate levels of resistin in comparison to Trolox equivalent antioxidant capacity for patients classified as early severe disease **(a)** and advanced severe disease **(b)**, resistin in comparison to malondialdehyde (MDA) for patients without obesity **(c)** and patients with obesity **(d)** and resistin in comparison to malondialdehyde (MDA) for patients classified as surviving **(e)** and non-surviving **(f)**.

In terms of redox indicators, neither AT hydrogen peroxide levels (as proxy for presence of reactive oxygen species), nor the indicator of oxidative damage, malonaldehyde (MDA), were associated with any of the stratification factors (**Figure 1**). However, total antioxidant capacity (TEAC) was significantly higher in the non-obese group (**Figure 1**). In order to interrogate the potential influence of obesity on antioxidant capacity in the context of COVID-19 disease outcome, data was assessed for potential correlations between AT TEAC and AT adipokine ratios associating with *mortality* in the current study. Indeed, while in non-obese patients, TEAC was positively correlated with leptin:resistin ratio, this correlation was lost in obesity (**Figures 2a, b**). No correlation was observed between TEAC and adiponectin:resistin ratio, regardless of obesity status (**Figures 2c, d**). Furthermore, resistin was positively correlated with TEAC in early severe disease (but not advanced severe disease) (**Figures 3a, b**) and negatively correlated to MDA in patients with obesity (but not in people without obesity) (**Figures 3c, d**), as well as in the patient group who did not survive (but not in the survivors) (**Figures 3e, f**).

### Adipose tissue SARS-CoV-2 RNA

Low levels of SARS-CoV-2 RNA were detected in only four (10.5%) sAT samples. All 4 had advanced severe disease (n=4/27, 15%), of whom 2 suffered from obesity and another from HIV. Three of the 8 patients who died and only 1 of the 30 who survived (n=3/8 (38%) vs 1/30 (3%), p = 0.02) had detectable SARS-CoV-2 RNA in sAT within 48 hours of hospital admission. None of the positive sequences belonged to the Delta variant.

## Discussion

Despite the relatively low sample number, our data aligns with other COVID-19 literature reporting cytokine and adipokine dysregulation in serum (22–26), underlining the significant magnitude of the dysregulation. For example, the finding of elevated concentrations of serum IL-6 in severe COVID-19 and the elevation of the anti-inflammatory cytokine IL-10 in serum -likely as compensatory mechanism - in patients who died, is in keeping with existing data that suggests hyperinflammation or a cytokine storm contributes to severity and outcomes in COVID-19 (27). IL-10 has also previously been shown to reflect a poor prognosis if elevated early in COVID-19 (27).

To the best of our knowledge, no other published studies on adipokine signaling in adipose tissue in COVID-19 exist. Thus, current data add to existing literature by also demonstrating similar adipokine dysregulation in the sAT - specifically higher leptin/resistin and lower adiponectin/resistin ratios, which were associated with COVID-19 mortality. Adipokine ratios are novel biomarkers that are more sensitive indicators of AT dysfunction and dysregulation of adipokine signaling than adipokines alone and have also been reported in the COVID-19 context (24, 28). This result is also somewhat different from the majority of relevant COVID-related literature which seems to link increase *serum* inflammatory cytokine and adipokine content to *severity* of disease, but not necessarily mortality (19, 24, 28, 29). Importantly, while *serum* adipokine levels did not sensitively predict mortality in our cohort either, AT adipokine ratios did, which confirms adipose tissue – and by implication pre-existing obesity - as a primary contributor to adipokine dysregulation of immune activation that reaches mortal magnitude in COVID disease. Regardless of the specific molecular mechanisms involved – which remain to be elucidated (5, 28, 30) – this result alone, underlines the urgency of addressing obesity itself as a “therapeutic target”, not only to alleviate risk for chronic inflammatory diseases linked to obesity, but also as a measure of future pandemic preparedness. In this context, the recent advances on anti-obesity drug development made by leading pharmaceutical giants – if made available at affordable prices – could significantly and beneficially impact mortality risk in infectious pandemics in future.

The current data set may be too modest to provide conclusive answers in terms of viral distribution and sequestration. However, the fact that detectable levels of SARS-CoV-2 RNA in sAT biopsies performed early after hospital admission, was associated with advanced severe disease and increased risk of mortality in the current cohort, could suggest adipose tissue as a viral reservoir. In support of this interpretation, previous studies have demonstrated the virus in 49% to 56% of various adipose depots in postmortem samples (6–8, 31). This should be further investigated in larger populations (or perhaps preclinical models at this point), as sequestration of viral RNA in adipose tissue may further exacerbate already present obesity-associated signaling dysregulation.

Of interest in terms of mechanisms, the relationships between resistin and redox indicators TEAC and MDA reported here, suggest uncoupling of the immune vs metabolic role of resistin in determining disease severity and outcome respectively.

Current results, despite the relatively low sample size, reflect the same profile, confirming the robustness of these observations. Analysis of relationships between different signaling role players in sAT in the current study, suggests a significant role for resistin and its interplay with other adipokines. The stratification of correlations between resistin and redox indicators according to obesity, disease severity and outcome, suggests that disease severity may be determined by immune-linked resistin function, while mortality is linked to a metabolic function of resistin, similar to what is observed in obesity. Resistin is known to have pro-inflammatory effects (18–20). Patients with early severe disease exhibited the ability to upregulate AT TEAC with increasing resistin, while those with more advanced severe disease were unable to do so, suggesting failure of endogenous antioxidant systems. This relationship between AT TEAC and resistin was not evident when stratifying for disease outcome, but AT TEAC exhibited a positive correlation to leptin:resistin ratio in non-obese individuals, which was lost in obesity. Given that increased leptin:resistin ratio was associated with COVID-19 mortality, these data suggest that the mortality associated with increased leptin:resistin ratio, may be due to failure of the endogenous antioxidant mechanisms to adequately respond in patients with obesity.

In contrast, both obesity and mortality are linked to a negative correlation between resistin and MDA (an indicator of oxidative damage to cell membranes) (32), arguing against early oxidative damage as determining factor. Considering that resistin is also known to impair glucose uptake into tissue (33), the hyperleptinaemia observed in sAT is suggestive of leptin resistance and a more metabolic role for resistin in these groups. This is in line with the finding that oxidative damage (MDA) did not parallel increases in resistin in obese and non-surviving patients. Obesity causes leptin resistance even before SARS-CoV-2 infection, thus dysregulating sAT leptin/resistin signaling in a manner shifting the role of resistin to a metabolic, rather than an immune function. Given the known effect of resistin on glucose transport, and the presence of a diabetic-like state in COVID-19 patients (22) – which may be exacerbated by cortisol action triggered by the elevated IL-10 secretion in the cytokine storm - we propose that metabolic dysregulation is also a contributor to mortality. This is supported in part by the early elevation of IL-10 reflecting a poor prognosis in COVID-19 (27). Although our interpretation is currently largely speculative, it is in line with current literature. The notion that therapy targeting resistin action in a way that uncouples metabolic vs immune pathways, may present greater versatility in terms of treatment of especially those patients with comorbidities such as obesity, warrants mechanistic studies to fully elucidate future therapeutic targets and biomarkers. In the current study, HIV did not have predictive power, which further supports a significant metabolic component as determinant of outcome.

### Strengths and limitations

This is the first study to characterize the relationships between adiponectin, leptin and resistin in sAT and patients with COVID-19, stratified for disease outcome, obesity and disease severity. This is also the only study of adipose tissue in COVID-19 patients which includes PWH. Given the exploratory nature of this study, there are several limitations which should be considered when interpreting the findings. Considering the small sample, we report the unadjusted p-values which should be interpreted as hypotheses or trends rather than as definitive conclusions, When correcting for multiple comparisons, the adjusted p-values were not below the 5% significance level but this may be a reflection of the increased risk of Type II error. These findings should therefore be interpreted in context of the sample size limitations, however the trends or hypothesis derived from the unadjusted data are still useful and may provide information on areas that should be validated in a larger cohort. In this analysis we corrected for confounders, however it is possible confounding still occurred. We used Factored ANOVA instead of multivariable regression because of sample size limitations. Factors such as smoking, alcohol and drug use, diet and other co-infections, which are likely to affect hormonal and cytokine responses, may also impact the interpretation of the findings. In addition, the study was conducted at only one site and caution must be taken when generalizing the data to other populations and time periods. Because adipose tissue was obtained from awake patients (as opposed to postmortem or intra-operative sampling), testing was limited to a small volume specimen. Further analysis of biobanked samples should include tests such as immunohistochemistry to definitively characterize viral particles in tissue compartments vs. blood in the adipose tissue. Inclusion of a SARS-CoV-2 negative control group and a larger sample size would have strengthened the study findings and should be a consideration in the design of future studies of this nature. Nevertheless, the current data aligns with findings of studies from larger cohorts, supporting the robustness of our more modest data set.

Further research is needed to explore the role of pharmacological and non-pharmacological interventions to address leptin resistance and especially the function of resistin with consideration of metabolic vs immune-linked pathways. The feasibility of this approach is evident from emerging reports (34). There is also role for similar studies in other viral infections, such as influenza, where obesity is a risk factor for mortality.

## Conclusion

Current data suggests a role for resistin in COVID-19 outcome. Although the assessment of sAT for biomarkers is not a feasible approach in an emergency patient care setting, the current study sheds light on the potential role of resistin in disease outcome. Further research is needed to investigate whether targeted therapy to regulate adipokine interplay has potential to reduce mortality in COVID-19. Future pandemic preparedness plans should prioritize public health and pharmaceutical interventions to target obesity itself as therapeutic target, including in low to middle income settings.

## Data Availability

The raw data supporting the conclusions of this article will be made available by the authors, without undue reservation.

## Author disclaimer

The opinions, findings and conclusions expressed in this manuscript reflect those of the authors alone. Given that this research was funded, in part, by the Wellcome Trust for the purposes of open access, GM has applied a CC BY public copyright license to any Author Accepted Manuscript version arising from this submission.

## References

[1] Ryan PMD Caplice NM. Is adipose tissue a reservoir for viral spread, immune activation, and cytokine amplification in coronavirus disease 2019? In: Obesity, vol. 28. New Jersey, United States: Blackwell Publishing Inc (2020). p. 1191–4. doi: 10.1002/oby.22843 PMC7264526 32314868

[2] Cypess AM. Reassessing human adipose tissue. New Engl J Med. (2022) 386:768–79. doi: 10.1056/NEJMra2032804 35196429

[3] Gao M Piernas C Astbury NM Hippisley-Cox J O’Rahilly S Aveyard P. Associations between body-mass index and COVID-19 severity in 6·9 million people in England: a prospective, community-based, cohort study. Lancet Diabetes Endocrinol. (2021) 9:350–9. doi: 10.1016/S2213-8587(21)00089-9 PMC8081400 33932335

[4] Parker A Boloko L Moolla MS Ebrahim N Ayele BT Broadhurst AGB. Clinical features and outcomes of COVID-19 admissions in a population with a high prevalence of HIV and tuberculosis: a multicentre cohort study. BMC Infect Dis. (2022) 22. doi: 10.1186/s12879-022-07519-8 PMC9207843 35725387

[5] Maurya R Sebastian P Namdeo M Devender M Gertler A. COVID-19 severity in obesity: leptin and inflammatory cytokine interplay in the link between high morbidity and mortality. Front Immunol. (2021) 12:649359. doi: 10.3389/fimmu.2021.649359 34220807 PMC8250137

[6] Saccon TD Mousovich-Neto F Ludwig RG Carregari VC dos Anjos Souza AB dos Passos ASC. SARS-CoV-2 infects adipose tissue in a fat depot- and viral lineage-dependent manner. Nat Commun. (2022) 13. doi: 10.1038/s41467-022-33218-8 PMC9521555 36175400

[7] Basolo A Poma AM Bonuccelli D Proietti A Macerola E Ugolini C. Adipose tissue in COVID-19: detection of SARS-CoV-2 in adipocytes and activation of the interferon-alpha response. J Endocrinol Invest. (2022) 45:1021–9. doi: 10.1007/s40618-022-01742-5 PMC8852916 35169984

[8] Martínez-Colón GJ Ratnasiri K Chen H Jiang S Zanley E Rustagi A. SARS-CoV-2 infection drives an inflammatory response in human adipose tissue through infection of adipocytes and macrophages. Sci Transl Med. (2022) 14. doi: 10.1126/scitranslmed.abm9151 PMC9529056 36137009

[9] Li MY Li L Zhang Y Wang XS. Expression of the SARS-CoV-2 cell receptor gene ACE2 in a wide variety of human tissues. Infect Dis Poverty. (2020) 9. doi: 10.1186/s40249-020-00662-x PMC7186534 32345362

[10] Al-Benna S. Association of high level gene expression of ACE2 in adipose tissue with mortality of COVID-19 infection in obese patients. Obes Med. (2020) 19. doi: 10.1016/j.obmed.2020.100283 PMC7368415 32835126

[11] Yu L Zhang X Ye S Lian H Wang H Ye J. Obesity and COVID-19: mechanistic insights from adipose tissue. J Clin Endocrinol Metab Endocrine Soc. (2022) 107:1799–811. doi: 10.1210/clinem/dgac137 PMC8992328 35262698

[12] Cao X Nguyen V Tsai J Gao C Tian Y Zhang Y. The SARS-CoV-2 spike protein induces long-term transcriptional perturbations of mitochondrial metabolic genes, causes cardiac fibrosis, and reduces myocardial contractile in obese mice. Mol Metab. (2023) 74:101756. doi: 10.1016/j.molmet.2023.101756 37348737 PMC10281040

[13] Obradovic M Sudar-Milovanovic E Soskic S Essack M Arya S Stewart AJ. Leptin and obesity: role and clinical implication. Front Endocrinol (Lausanne). (2021) 12:585887. doi: 10.3389/fendo.2021.585887 34084149 PMC8167040

[14] Achari AE Jain SK. Adiponectin, a therapeutic target for obesity, diabetes, and endothelial dysfunction. Int J Mol Sci MDPI AG. (2017) 18. doi: 10.3390/ijms18061321 PMC5486142 28635626

[15] Ouchi N Walsh K. Adiponectin as an anti-inflammatory factor. Clinica Chimica Acta. (2007) 380:24–30. doi: 10.1016/j.cca.2007.01.02 PMC2755046 17343838

[16] Guglielmi V Colangeli L D’Adamo M Sbraccia P. Susceptibility and severity of viral infections in obesity: lessons from influenza to COVID-19. Does leptin play a role? Int J Mol Sci. (2021) 22:3183. doi: 10.3390/ijms22063183 33804765 PMC8003928

[17] Lazar MA. Resistin- and Obesity-associated metabolic diseases. Horm Metab Res. (2007) 39:710–6. doi: 10.1055/s-2007-985897 17952831

[18] Acquarone E Monacelli F Borghi R Nencioni A Odetti P. Resistin: A reappraisal. Mech Ageing Dev. (2019) 178:46–63. doi: 10.1016/j.mad.2019.01.004 30650338

[19] de Nooijer AH Kooistra EJ Grondman I Janssen NAF Joosten LAB van de Veerdonk FL. Adipocytokine plasma concentrations reflect influence of inflammation but not body mass index (BMI) on clinical outcomes of COVID-19 patients: A prospective observational study from the Netherlands. Clin Obes. (2023) 13. doi: 10.1111/cob.12568 36426776

[20] Lin Q Johns RA. Resistin family proteins in pulmonary diseases. Am J Physiol Lung Cell Mol Physiol. (2020) 319:422–34. doi: 10.1152/ajplung.00040.2020 PMC7518061 32692581

[21] Choi HM Doss HM Kim KS. Multifaceted physiological roles of adiponectin in inflammation and diseases. Int J Mol Sci MDPI AG. (2020) 21. doi: 10.3390/ijms21041219 PMC7072842 32059381

[22] Petrushevska M Zendelovska D Atanasovska E Eftimov A Spasovska K. Presentation of cytokine profile in relation to oxidative stress parameters in patients with severe COVID-19: a case-control pilot study. F1000Res. (2021) 10:719. doi: 10.12688/f1000research.55166.2 34868558 PMC8603313

[23] Tonon F Di Bella S Giudici F Zerbato V Segat L Koncan R. Discriminatory value of adiponectin to leptin ratio for COVID-19 pneumonia. Int J Endocrinol. (2022) 2022:9908450. doi: 10.1155/2022/9908450 35529082 PMC9072020

[24] Di Filippo L De Lorenzo R Sciorati C Capobianco A Lorè NI Giustina A. Adiponectin to leptin ratio reflects inflammatory burden and survival in COVID-19: Adiponectin and leptin in COVID-19. Diabetes Metab. (2021) 47. doi: 10.1016/j.diabet.2021.101268 PMC8320244 34333093

[25] Wang J Xu Y Zhang X Wang S Peng Z Guo J. Leptin correlates with monocytes activation and severe condition in COVID-19 patients. J Leukoc Biol. (2021) 110:9–20. doi: 10.1002/JLB.5HI1020-704R 33404078 PMC10016867

[26] Larsson A Lipcsey M Hultström M Frithiof R Eriksson M. Plasma leptin is increased in intensive care patients with COVID-19-an investigation performed in the pronMed-cohort. Biomedicines. (2021) 10:4. doi: 10.3390/biomedicines10010004 35052684 PMC8773415

[27] Lu L Zhang H Dauphars DJ He YW. A potential role of interleukin 10 in COVID-19 pathogenesis. Trends Immunol. (2021) 42:3–5. doi: 10.1016/j.it.2020.10.012 33214057 PMC7605819

[28] Perrotta F Scialò F Mallardo M Signoriello G D’Agnano V Bianco A. Adiponectin, leptin, and resistin are dysregulated in patients infected by SARS-cov-2. Int J Mol Sci. (2023) 24. doi: 10.3390/ijms24021131 PMC9861572 36674646

[29] Flikweert AW Kobold ACM van-der-Sar-van-der-Brugge S Heeringa P Rodenhuis-Zybert IA Bijzet J. Circulating adipokine levels and COVID-19 severity in hospitalized patients. Int J Obes. (2023) 47:126–37. doi: 10.1038/s41366-022-01246-5 PMC9742670 36509969

[30] Sudhakar M Winfred SB Meiyazhagan G Venkatachalam DP. Mechanisms contributing to adverse outcomes of COVID-19 in obesity. Mol Cell Biochem. (2022) 477:1155–93. doi: 10.1007/s11010-022-04356-w PMC8793096 35084674

[31] Zickler M Stanelle-Bertram S Ehret S Heinrich F Lange P Schaumburg B. Replication of SARS-CoV-2 in adipose tissue determines organ and systemic lipid metabolism in hamsters and humans. Cell Metab Cell Press;. (2022) 34:1–2. doi: 10.1016/j.cmet.2021.12.002 PMC8663969 34895500

[32] Weiss SL Deutschman CS. Elevated malondialdehyde levels in sepsis-something to’stress’ about? Crit Care. (2014) 18:1–2. doi: 10.1186/cc13786 PMC4056888 25029036

[33] Palanivel R Maida A Liu Y Sweeney G. Regulation of insulin signalling, glucose uptake and metabolism in rat skeletal muscle cells upon prolonged exposure to resistin. Diabetologia. (2006) 49:183–90. doi: 10.1007/s00125-005-0060-z 16341686

[34] Zabeau L Wauman J Dam J Van Lint S Burg E De Geest J. A novel leptin receptor antagonist uncouples leptin’s metabolic and immune functions. Cell Mol Life Sci. (2019) 76:1201–14. doi: 10.1007/s00018-019-03004-9 PMC11105424 30659329

