# Supplementary material for: Pre-existing adipose tissue signaling profile related to obesity determines disease outcome of COVID-19: addressing obesity should be a priority for future pandemic preparedness
Source: Front Endocrinol (Lausanne). 2025 Apr 25;16:1506065. doi: 10.3389/fendo.2025.1506065 (PMC12061698; doi:10.3389/fendo.2025.1506065)
Supplement: Supplementary file 1 [file DataSheet1.pdf]

Supplementary Data:

1. Supplementary Tables 1 and 2
2. Supplementary Data 1: Obesity and adipokines
3. Supplementary Data 2: Other confounders and adipokines
4. Supplementary Data 3: Adiponectin Resistin Ratio, cytokines and cofounders

Supplementary Table 1: Adipose tissue and serum adipokine concentrations and adipokine ratios stratified by obesity, COVID-19 severity, and outcome

	**Obesity**				**COVID-19 severity**				**Outcome**			
**Adipose Tissue:**	**BMI ≥ 30**	**BMI < 30**	**p**	**p adjusted**	**Advanced Severe**	**Early Severe**	**p**	**p adjusted**	**Died**	**Survived**	**p**	**p adjusted**
Adiponectin*	1672 (1290)	1133 (1228)	0.21	0.63	1359 (1216)	1705 (1449)	0.46	0.73	1184 (1110)	1533 (1326)	0.5	0.74
Leptin*	**1683 (1477)**	**692 (963)**	**0.03**	0.29	1171 (1299)	1587 (1572)	0.40	0.68	1059 (999)	1354 (1467)	0.60	0.77
Resistin*	860 (1097)	635 (894)	0.51	0.74	775 (1088)	761 (857)	0.97	0.97	428 (749)	863 (1067)	0.29	0.63
Leptin/resistin*	5.6 (5.4)	2.8 (4.0)	0.09	0.59	5.0 (5.7)	3.2 (2.5)	0.34	0.63	**7.7 (7.2)**	**3.6 (4.0)**	**0.04**	0.35
Adiponectin/resistin*	17.2 (25.6)	17.9 (27.0)	0.94	0.97	19.2 (24.3)	17.0 (30.2)	0.81	0.97	**8.1 (8.5)**	**20.1 (28.2)**	**0.05**	0.39
Adiponectin/leptin*	**9.0 (14.42)**	**22.5 (21.8)**	**0.03**	0.29	16.5 (21.0)	9.0 (10.1)	0.27	0.63	19.0 (25.8)	13.1 (16.6)	0.44	0.73
**Serum:**												
Adiponectin (mg/ml)	10.04 (3.0)	10.08 (4.4)	0.97	0.97	10.1 (3.6)	10.0 (3.5)	0.95	0.97	10.5 (3.2)	10.0 (3.7)	0.67	0.84
Leptin (ug/ml)	**68 (52)**	**26 (25)**	**<0.01**	0.18	47 (48)	63 (49)	0.36	0.64	70 (71)	47 (40)	0.23	0.63
Resistin (ug/ml)	24 (7)	22 (10)	0.57	0.75	23 (9)	24 (7)	0.89	0.97	23 (8)	24 (10)	0.68	0.84
Leptin/resistin	**3 (2.2)**	**1.3 (1.1)**	**<0.01**	0.07	2.1 (1.9)	2.8 (2.2)	0.35	0.63	3.1 (2.6)	2.1 (1.8)	0.22	0.63
Adiponectin/resistin	460 (178)	558 (351)	0.26	0.63	514 (287)	462 (186)	0.58	0.75	415 (239)	495 (269)	0.85	0.97
Adiponectin/leptin*	**241 (214)**	**884 (956)**	**<0.01**	0.13	599 (792)	239 (160)	0.15	0.63	470 (657)	501 (708)	0.91	0.97

Data reflect the differences in the means (± standard deviations(SD)) or the *winsorised means (± SD) of the ratios or concentrations measured in picograms per milligrams (pg/mg) for adipose tissue and picograms per millilitre (pg/ml) for serum. mg/ml = milligram/millilitre. ug/ml = microgram/millilitre. *One way ANOVA was used to test for significance. Benjamini-Hochberg procedure (BH) was used to derive adjusted p-values*

Supplementary Table 2: Adipose Tissue and serum cytokine concentrations stratified by obesity, COVID-19 severity, and outcome

	Obesity				COVID-19 severity				Outcome			
**Adipose Tissue:**	**BMI ≥ 30**	**BMI < 30**	**p**	**p adjusted**	**Advanced Severe**	**Early severe**	**p**	**p adjusted**	**Died**	**Survived**	**p**	**p adjusted**
TNF-a*	0.4 (0.3)	0.3 (0.3)	0.18	0.63	0.3 (0.3)	0.4 (0.4)	0.35	0.63	0.2 (0.2)	0.4 (0.4)	0.18	0.63
IL-2	94 (76)	74 (95)	0.47	0.73	75 (66)	114 (115)	0.19	0.63	51 (39)	95 (90)	0.19	0.63
IL-1b	0.7 (0.6)	0.4 (0.5)	0.23	0.63	0.8 (0.7)	0.5 (0.5)	0.15	0.63	0.3 (0.2)	0.7 (0.6)	0.08	0.57
IL -6*	1.4 (1.2)	1.1 (1.2)	0.45	0.73	1.2 (1.1)	1.6 (1.5)	0.34	0.63	0.9 (0.7)	1.4 (1.3)	0.25	0.63
IL-17*	0.7 (0.5)	0.5 (0.5)	0.38	0.66	0.6 (0.4)	0.7 (0.6)	0.31	0.63	0.4 (0.4)	0.7 (0.5)	0.21	0.63
IL-10	1.1 (0.8)	0.8 (0.8)	0.33	0.63	0.8 (0.7)	1.3 (1.1)	0.16	0.63	0.7 (0.5)	1.1 (0.9)	0.27	0.63
MCP-1*	14.5 (12.6)	12.0 (11.9)	0.53	0.74	12.7 (11.6)	15.5 (14.0)	0.53	0.74	9.2 (8.4)	14.7 (12.9)	0.26	0.63
**Serum:**												
TNF-a	9.7 (5.3)	7.8 (3.8)	0.25	0.63	8.9 (4.7)	9.1 (5.1)	0.92	0.97	9.1 (3.6)	8.9 (5.1)	0.93	0.97
IL-2	149 (43)	140 (45)	0.55	0.74	142 (45)	153 (41)	0.51	0.74	145 (35)	145 (46)	0.97	0.97
IL-1b*	**5.5 (0.6)**	**5.2 (0.4)**	**0.03**	0.29	5.3 (0.6)	5.5 (0.5)	0.33	0.63	5.4 (0.7)	5.4 (0.5)	0.84	0.97
IL- 6	11.7 (11.3)	14.1 (13.2)	0.55	0.74	**15.3 (12.7)**	**6.2 (6.1)**	**0.03**	0.29	16.8 (13.4)	11.6 (11.6)	0.28	0.63
IL-17*	3.2 (2.8)	2.3 (2.4)	0.30	0.63	2.8 (2.7)	2.8 (2.8)	0.96	0.97	2.7 (2.9)	2.9 (2.7)	0.89	0.97
IL-10	5.1 (3.3)	5.0 (4.3)	0.94	0.97	5.5 (4.1)	3.9 (2.0)	0.23	0.63	**7.5 (5.2)**	**4.4 (2.9)**	**0.03**	0.29
MCP-1	408 (298)	580 (515)	0.20	0.63	516 (416)	378 (360)	0.34	0.63	540 (471)	459 (387)	0.52	0.74

Data reflect the differences in the means (± standard deviations) or the *winsorised means (± SD) of the concentrations of cytokines, measured in picograms per milligrams (pg/mg) for adipose tissue and picograms per millilitre (pg/ml) for serum. *One way ANOVA was used to test for significance. Benjamini-Hochberg procedure (BH) was used to derive adjusted p-values*

1) Adiponectin(BL)

1.1) ANOVA

	ANOVA type: III			
	Sum Sq	DF	F value	p value
(Intercept)	2.55815E+15	1	194.818	<0.01
Obesity	22425759298	1	0.002	0.97
Final outcome	2.43928E+12	1	0.186	0.67
Residuals	4.59585E+14	35		

2) Adiponectin(FI AT)(winsorized)

2.1) ANOVA

	ANOVA type: III			
	Sum Sq	DF	F value	p value
(Intercept)	41060119.1	1	25.26	<0.01
Obesity	2694608.46	1	1.66	0.21
Final outcome	828514.37	1	0.51	0.48
Residuals	56881632.03	35		

3) Adiponectin/Leptin(AT FI)(winsorized)

3.1) ANOVA

	ANOVA type: III			
	Sum Sq	DF	F value	p value
(Intercept)	7482.27	1	23.83	<0.01
Obesity	1667.81	1	5.31	0.03
Final outcome	240.63	1	0.77	0.39
Residuals	10989.94	35		

4) Adiponectin/Resistin(BL)

4.1) ANOVA

	ANOVA type: III			
	Sum Sq	DF	F value	p value
(Intercept)	6491126.78	1	94.24	<0.01
Obesity	87440.52	1	1.27	0.27
Final outcome	3306.48	1	0.05	0.83
Residuals	2410659.24	35		

5) Leptin(BL)

5.1) ANOVA

	ANOVA type: III			
	Sum Sq	DF	F value	p value
(Intercept)	70531070340	1	37.7	<0.01
Obesity	15655187790	1	8.37	<0.01
Final outcome	3010575280	1	1.61	0.21
Residuals	65484181294	35		

6) Leptin/Resistin(AT)(winsorized)

6.1) ANOVA

	ANOVA type: III			
	Sum Sq	DF	F value	p value
(Intercept)	703.7	1	32.34	<0.01
Obesity	69.56	1	3.2	0.08
Final outcome	103.77	1	4.77	0.04
Residuals	761.52	35		

7) leptin/Resistin(BL)

7.1) ANOVA

	ANOVA type: III			
	Sum Sq	DF	F value	p value
(Intercept)	142.87	1	42.25	<0.01
Obesity	26.36	1	7.8	<0.01
Final outcome	5.75	1	1.7	0.2
Residuals	118.34	35		

8) Resistin(BL)

8.1) ANOVA

	ANOVA type: III			
	Sum Sq	DF	F value	p value
(Intercept)	13404725802	1	192.34	<0.01
Obesity	22249294.6	1	0.32	0.58
Final outcome	10800714.23	1	0.15	0.7
Residuals	2439281567	35		

9) Adiponectin/leptin(BL)(winsorized)

9.1) ANOVA

	ANOVA type: III			
	Sum Sq	DF	F value	p value
(Intercept)	7595553.47	1	19.26	<0.01
Obesity	3758114.58	1	9.529	<0.01
Final outcome	1508.92	1	0.004	0.95
Residuals	13803194.25	35		

10) Adiponectin/Resistin(AT F1)(winsorized)

10.1) ANOVA

	ANOVA type: III			
	Sum Sq	DF	F value	p value
(Intercept)	4794.51	1	7.11	0.01
Obesity	6.9	1	0.01	0.92
Final outcome	916.42	1	1.36	0.25
Residuals	23616.06	35		

11) Leptin(AT)(winsorized)

11.1) ANOVA

	ANOVA type: III			
	Sum Sq	DF	F value	p value
(Intercept)	29222280.02	1	16.95	<0.01
Obesity	9026611.88	1	5.24	0.03
Final outcome	644190.55	1	0.37	0.54
Residuals	60346842.42	35		

12) Resistin(AT)(winsorized)

12.1) ANOVA

	ANOVA type: III			
	Sum Sq	DF	F value	p value
(Intercept)	9357372.3	1	8.99	<0.01
Obesity	493188.92	1	0.47	0.5
Final outcome	1221974.43	1	1.17	0.29
Residuals	36441054.54	35		

1) R lm

1.1) Adiponectin(BL) with HIV

1.1.1) ANOVA

	ANOVA type: III			
	Sum Sq	DF	F value	p value
(Intercept)	2.44362E+15	1	202.23	<0.01
HIV	3.66942E+13	1	3.04	0.09
Final outcome	2.05174E+12	1	0.17	0.68
Residuals	4.22913E+14	35		

1.2) Adiponectin(FI AT)(winsorized) with HIV

1.2.1) ANOVA

	ANOVA type: III			
	Sum Sq	DF	F value	p value
(Intercept)	45900286.66	1	27.02	<0.01
HIV	121580.54	1	0.07	0.79
Final outcome	754689.9	1	0.44	0.51
Residuals	59454659.96	35		

1.3) Adiponectin/Leptin(AT FI)(winsorized) with HIV

1.3.1) ANOVA

	ANOVA type: III			
	Sum Sq	DF	F value	p value
(Intercept)	5525.86	1	16.08	<0.01
HIV	629.86	1	1.83	0.18
Final outcome	199.87	1	0.58	0.45
Residuals	12027.88	35		

1.4) Adiponectin/Resistin(BL) with HIV

1.4.1) ANOVA

	ANOVA type: III			
	Sum Sq	DF	F value	p value
(Intercept)	6005970.28	1	85.97	<0.01
HIV	53094.76	1	0.76	0.39
Final outcome	2165.18	1	0.03	0.86
Residuals	2445005.01	35		

1.5) Leptin(BL) with HIV

1.5.1) ANOVA

	ANOVA type: III			
	Sum Sq	DF	F value	p value
(Intercept)	82026831044	1	35.45	<0.01
HIV	149559540.9	1	0.06	0.8
Final outcome	3274432192	1	1.42	0.24
Residuals	80989809544	35		

1.6) Leptin/Resistin(AT)(winsorized) with HIV

1.6.1) ANOVA

	ANOVA type: III			
	Sum Sq	DF	F value	p value
(Intercept)	786.14	1	33.1083	<0.01
HIV	0.02	1	0.001	0.98
Final outcome	107.28	1	4.5181	0.04
Residuals	831.06	35		

1.7) leptin/Resistin(BL) with HIV

1.7.1) ANOVA

	ANOVA type: III			
	Sum Sq	DF	F value	p value
(Intercept)	169.88	1	41.24	<0.01
HIV	0.53	1	0.13	0.72
Final outcome	6.35	1	1.54	0.22
Residuals	144.18	35		

1.8) Resistin(BL) with HIV

1.8.1) ANOVA

	ANOVA type: III			
	Sum Sq	DF	F value	p value
(Intercept)	13403018673	1	192.34	<0.01
HIV	22627903.75	1	0.32	0.57
Final outcome	10795237.72	1	0.15	0.7
Residuals	2438902957	35		

1.9) Adiponectin/leptin(BL)(winsorized) with HIV

1.9.1) ANOVA

	ANOVA type: III			
	Sum Sq	DF	F value	p value
(Intercept)	5791030.99	1	11.543	<0.01
HIV	1921.68	1	0.004	0.95
Final outcome	6139.21	1	0.012	0.91

Residuals	17559387.15	35		

1.10) Adiponectin/Resistin(AT F1)(winsorized) with HIV

1.10.1) ANOVA

	ANOVA type: III			
	Sum Sq	DF	F value	p value
(Intercept)	5140.24	1	7.65	<0.01
HIV	113.84	1	0.17	0.68
Final outcome	899.71	1	1.34	0.25
Residuals	23509.12	35		

1.11) Leptin(AT)(winsorized) with HIV

1.11.1) ANOVA

	ANOVA type: III			
	Sum Sq	DF	F value	p value
(Intercept)	36829492.33	1	18.67	<0.01
HIV	347199.01	1	0.18	0.68
Final outcome	529520.6	1	0.27	0.61
Residuals	69026255.3	35		

1.12) Resistin(AT)(winsorized) with HIV

1.12.1) ANOVA

	ANOVA type: III			
	Sum Sq	DF	F value	p value
(Intercept)	9926340.68	1	9.42	<0.01
HIV	42271.58	1	0.04	0.84
Final outcome	1199185.23	1	1.14	0.29
Residuals	36891971.88	35		

1.13) Adiponectin(BL) with severity

1.13.1) ANOVA

	ANOVA type: III			
	Sum Sq	DF	F value	p value
(Intercept)	1.95317E+15	1	148.765	<0.01
severity	84238010207	1	0.006	0.94
Final outcome	2.4573E+12	1	0.187	0.67
Residuals	4.59523E+14	35		

1.14) Adiponectin(FI AT)(winsorized) with severity

1.14.1) ANOVA

	ANOVA type: III			
	Sum Sq	DF	F value	p value
(Intercept)	38661188.37	1	22.91	<0.01
severity	515588.52	1	0.31	0.58
Final outcome	348752.15	1	0.21	0.65
Residuals	59060651.98	35		

1.15) Adiponectin/Leptin(AT FI)(winsorized) with severity

1.15.1) ANOVA

	ANOVA type: III			
	Sum Sq	DF	F value	p value
(Intercept)	3602.65	1	10.19	<0.01
severity	289.17	1	0.82	0.37
Final outcome	67.89	1	0.19	0.66
Residuals	12368.58	35		

1.16) Adiponectin/Resistin(BL) with severity

1.16.1) ANOVA

	ANOVA type: III			
	Sum Sq	DF	F value	p value
(Intercept)	4415558.09	1	62.3369	<0.01
severity	18918.14	1	0.2671	0.61
Final outcome	9.87	1	0.0001	0.99
Residuals	2479181.63	35		

1.17) Leptin(BL) with severity

1.17.1) ANOVA

	ANOVA type: III			
	Sum Sq	DF	F value	p value
(Intercept)	81876331629	1	37.39	<0.01
severity	4497224980	1	2.05	0.16
Final outcome	5834130549	1	2.66	0.11
Residuals	76642144105	35		

1.18) Leptin/Resistin(AT)(winsorized) with severity

1.18.1) ANOVA

	ANOVA type: III			
	Sum Sq	DF	F value	p value
(Intercept)	558.91	1	23.61	<0.01

severity	2.48	1	0.1	0.75
Final outcome	85.84	1	3.63	0.07
Residuals	828.61	35		

1.19) leptin/Resistin(BL) with severity

1.19.1) ANOVA

	ANOVA type: III			
	Sum Sq	DF	F value	p value
(Intercept)	162.44	1	41.74	<0.01
severity	8.5	1	2.18	0.15
Final outcome	11.07	1	2.84	0.1
Residuals	136.21	35		

1.20) Resistin(BL) with severity

1.20.1) ANOVA

	ANOVA type: III			
	Sum Sq	DF	F value	p value
(Intercept)	10581502020	1	150.8	<0.01
severity	5670774.49	1	0.08	0.78
Final outcome	15850375.58	1	0.23	0.64
Residuals	2455860087	35		

1.21) Adiponectin/leptin(BL)(winsorized) with severity

1.21.1) ANOVA

	ANOVA type: III			
	Sum Sq	DF	F value	p value
(Intercept)	2327644.57	1	4.98	0.03
severity	1193056.53	1	2.55	0.12
Final outcome	189117.18	1	0.4	0.53
Residuals	16368252.3	35		

1.22) Adiponectin/Resistin(AT F1)(winsorized) with severit

1.22.1) ANOVA

	ANOVA type: III			
	Sum Sq	DF	F value	p value
(Intercept)	3447.78	1	5.11	0.03
severity	15.54	1	0.02	0.88
Final outcome	890.1	1	1.32	0.26
Residuals	23607.42	35		

1.23) Leptin(AT)(winsorized) with severity

1.23.1) ANOVA

	ANOVA type: III			
	Sum Sq	DF	F value	p value
(Intercept)	32440829.2	1	16.59	<0.01
severity	947612.99	1	0.48	0.49
Final outcome	142815.76	1	0.07	0.79
Residuals	68425841.31	35		

1.24) Resistin(AT)(winsorized) with severity

1.24.1) ANOVA

	ANOVA type: III			
	Sum Sq	DF	F value	p value
(Intercept)	6553006.67	1	6.24	0.02
severity	178710.44	1	0.17	0.68
Final outcome	1367481.49	1	1.3	0.26
Residuals	36755533.02	35		

1.25) Adiponectin(BL) with Sex

1.25.1) ANOVA

	ANOVA type: III			
	Sum Sq	DF	F value	p value
(Intercept)	2.00388E+15	1	152.85	<0.01
Sex	7.64566E+11	1	0.06	0.81
Final outcome	2.7668E+12	1	0.21	0.65
Residuals	4.58842E+14	35		

1.26) Adiponectin(FI AT)(winsorized) with Sex

1.26.1) ANOVA

	ANOVA type: III			
	Sum Sq	DF	F value	p value
(Intercept)	31810605.25	1	18.76	<0.01
Sex	230496.55	1	0.14	0.71
Final outcome	871026.65	1	0.51	0.48
Residuals	59345743.95	35		

1.27) Adiponectin/Leptin(AT FI)(winsorized) with Sex

1.27.1) ANOVA

	ANOVA type: III			
	Sum Sq	DF	F value	p value

(Intercept)	9027.19	1	31.35	<0.01
Sex	2580.91	1	8.96	<0.01
Final outcome	459.22	1	1.6	0.21
Residuals	10076.83	35		

1.28) Adiponectin/Resistin(BL) with Sex

1.28.1) ANOVA

	ANOVA type: III			
	Sum Sq	DF	F value	p value
(Intercept)	5474207.26	1	79.62	<0.01
Sex	91798.08	1	1.34	0.26
Final outcome	8469.66	1	0.12	0.73
Residuals	2406301.69	35		

1.29) Leptin(BL) with Sex

1.29.1) ANOVA

	ANOVA type: III			
	Sum Sq	DF	F value	p value
(Intercept)	37732251230	1	19.51	<0.01
Sex	13448154208	1	6.95	0.01
Final outcome	1697723367	1	0.88	0.36
Residuals	67691214877	35		

1.30) Leptin/Resistin(AT)(winsorized) with Sex

1.30.1) ANOVA

	ANOVA type: III			
	Sum Sq	DF	F value	p value
(Intercept)	517.28	1	22.14	<0.01
Sex	13.49	1	0.58	0.45
Final outcome	95.41	1	4.08	0.05
Residuals	817.59	35		

1.31) leptin/Resistin(BL) with Sex

1.31.1) ANOVA

	ANOVA type: III			
	Sum Sq	DF	F value	p value
(Intercept)	74.68	1	22.33	<0.01
Sex	27.66	1	8.27	<0.01
Final outcome	3.13	1	0.93	0.34
Residuals	117.04	35		

1.32) Resistin(BL) with Sex

1.32.1) ANOVA

	ANOVA type: III			
	Sum Sq	DF	F value	p value
(Intercept)	10144469906	1	144.79	<0.01
Sex	9384933.85	1	0.13	0.72
Final outcome	8631736.35	1	0.12	0.73
Residuals	2452145927	35		

1.33) Adiponectin/leptin(BL)(winsorized) with Sex

1.33.1) ANOVA

	ANOVA type: III			
	Sum Sq	DF	F value	p value
(Intercept)	10642049.95	1	30.18	<0.01
Sex	5221463.79	1	14.81	<0.01
Final outcome	53763.8	1	0.15	0.7
Residuals	12339845.04	35		

1.34) Adiponectin/Resistin(AT F1)(winsorized) with Sex

1.34.1) ANOVA

	ANOVA type: III			
	Sum Sq	DF	F value	p value
(Intercept)	3421.86	1	5.08	0.03
Sex	27.03	1	0.04	0.84
Final outcome	939.44	1	1.39	0.25
Residuals	23595.94	35		

1.35) Leptin(AT)(winsorized) with Sex

1.35.1) ANOVA

	ANOVA type: III			
	Sum Sq	DF	F value	p value
(Intercept)	19676364.31	1	10.29	<0.01
Sex	2422776.94	1	1.27	0.27
Final outcome	892493.58	1	0.47	0.5
Residuals	66950677.36	35		

1.36) Resistin(AT)(winsorized) with Sex

1.36.1) ANOVA

	ANOVA type: III

	Sum Sq	DF	F value	p value
(Intercept)	8413926.13	1	7.98	<0.01
Sex	42879.84	1	0.04	0.84
Final outcome	1108342.67	1	1.05	0.31
Residuals	36891363.63	35		

1.37) Adiponectin(BL) with Hypertension

1.37.1) ANOVA

	ANOVA type: III			
	Sum Sq	DF	F value	p value
(Intercept)	2.54045E+15	1	196.73	<0.01
Hypertension	7.63809E+12	1	0.59	0.45
Final outcome	3.85456E+12	1	0.3	0.59
Residuals	4.51969E+14	35		

1.38) Adiponectin(FI AT)(winsorized) with Hypertension

1.38.1) ANOVA

	ANOVA type: III			
	Sum Sq	DF	F value	p value
(Intercept)	37318603.22	1	23.32	<0.01
Hypertension	3563784.28	1	2.23	0.14
Final outcome	1332986.58	1	0.83	0.37
Residuals	56012456.21	35		

1.39) Adiponectin/Leptin(AT FI)(winsorized) with Hypertens

1.39.1) ANOVA

	ANOVA type: III			
	Sum Sq	DF	F value	p value
(Intercept)	7776.3	1	24.78	<0.01
Hypertension	1673.52	1	5.33	0.03
Final outcome	430.43	1	1.37	0.25
Residuals	10984.23	35		

1.40) Adiponectin/Resistin(BL) with Hypertension

1.40.1) ANOVA

	ANOVA type: III			
	Sum Sq	DF	F value	p value
(Intercept)	6236534.62	1	88.53	<0.01
Hypertension	32523.38	1	0.46	0.5
Final outcome	6133.98	1	0.09	0.77
Residuals	2465576.38	35		

1.41) Leptin(BL) with Hypertension

1.41.1) ANOVA

	ANOVA type: III			
	Sum Sq	DF	F value	p value
(Intercept)	73818955695	1	32.72	<0.01
Hypertension	2176298323	1	0.96	0.33
Final outcome	2468169035	1	1.09	0.3
Residuals	78963070762	35		

1.42) Leptin/Resistin(AT)(winsorized) with Hypertension

1.42.1) ANOVA

	ANOVA type: III			
	Sum Sq	DF	F value	p value
(Intercept)	774.49	1	32.65	<0.01
Hypertension	0.88	1	0.04	0.85
Final outcome	107.84	1	4.55	0.04
Residuals	830.21	35		

1.43) leptin/Resistin(BL) with Hypertension

1.43.1) ANOVA

	ANOVA type: III			
	Sum Sq	DF	F value	p value
(Intercept)	147.86	1	36.8	<0.01
Hypertension	4.06	1	1.01	0.32
Final outcome	4.7	1	1.17	0.29
Residuals	140.65	35		

1.44) Resistin(BL) with Hypertension

1.44.1) ANOVA

	ANOVA type: III			
	Sum Sq	DF	F value	p value
(Intercept)	13305766725	1	189.74	<0.01
Hypertension	7119829.33	1	0.1	0.75
Final outcome	14090505.33	1	0.2	0.66
Residuals	2454411032	35		

1.45) Adiponectin/leptin(BL)(winsorized) with Hypertensio

1.45.1) ANOVA

	ANOVA type: III			
	Sum Sq	DF	F value	p value
(Intercept)	6389693.57	1	13.058	<0.01
Hypertension	434904.74	1	0.889	0.35
Final outcome	505.73	1	0.001	0.97
Residuals	17126404.09	35		

1.46) Adiponectin/Resistin(AT F1)(winsorized) with Hyperte

1.46.1) ANOVA

	ANOVA type: III			
	Sum Sq	DF	F value	p value
(Intercept)	4483.78	1	6.65	0.01
Hypertension	37.36	1	0.06	0.82
Final outcome	948.85	1	1.41	0.24
Residuals	23585.6	35		

1.47) Leptin(AT)(winsorized) with Hypertension

1.47.1) ANOVA

	ANOVA type: III			
	Sum Sq	DF	F value	p value
(Intercept)	28407995.49	1	15.25	<0.01
Hypertension	4190856.66	1	2.25	0.14
Final outcome	1090890.9	1	0.59	0.45
Residuals	65182597.65	35		

1.48) Resistin(AT)(winsorized) with Hypertension

1.48.1) ANOVA

	ANOVA type: III			
	Sum Sq	DF	F value	p value
(Intercept)	8283123.91	1	8.06	<0.01
Hypertension	984391.16	1	0.96	0.33
Final outcome	1512667.29	1	1.47	0.23
Residuals	35949852.3	35		

1.49) Adiponectin(BL) with Diabetes

1.49.1) ANOVA

	ANOVA type: III			
	Sum Sq	DF	F value	p value
(Intercept)	2.18201E+15	1	174.3	<0.01
Diabetes	2.14639E+13	1	1.71	0.2
Final outcome	4.63633E+12	1	0.37	0.55

Residuals	4.38143E+14	35		

1.50) Adiponectin(FI AT)(winsorized) with Diabetes

1.50.1) ANOVA

	ANOVA type: III			
	Sum Sq	DF	F value	p value
(Intercept)	45111470.45	1	26.86	<0.01
Diabetes	783977.63	1	0.47	0.5
Final outcome	969735.4	1	0.58	0.45
Residuals	58792262.86	35		

1.51) Adiponectin/Leptin(AT FI)(winsorized) with Diabetes

1.51.1) ANOVA

	ANOVA type: III			
	Sum Sq	DF	F value	p value
(Intercept)	5032.49	1	14.11	<0.01
Diabetes	172.82	1	0.48	0.49
Final outcome	264.44	1	0.74	0.4
Residuals	12484.93	35		

1.52) Adiponectin/Resistin(BL) with Diabetes

1.52.1) ANOVA

	ANOVA type: III			
	Sum Sq	DF	F value	p value
(Intercept)	5050546.76	1	75.43	<0.01
Diabetes	154555.65	1	2.31	0.14
Final outcome	10499.4	1	0.16	0.69
Residuals	2343544.11	35		

1.53) Leptin(BL) with Diabetes

1.53.1) ANOVA

	ANOVA type: III			
	Sum Sq	DF	F value	p value
(Intercept)	73862181070	1	31.92	<0.01
Diabetes	139144384.9	1	0.06	0.81
Final outcome	3427008612	1	1.48	0.23
Residuals	81000224700	35		

1.54) Leptin/Resistin(AT)(winsorized) with Diabetes

1.54.1) ANOVA

	ANOVA type: **III**			
	Sum Sq	DF	F value	p value
(Intercept)	619.22	1	27.11	<0.01
Diabetes	31.71	1	1.39	0.25
Final outcome	121.27	1	5.31	0.03
Residuals	799.37	35		

1.55) leptin/Resistin(BL) with Diabetes

1.55.1) ANOVA

	ANOVA type: **III**			
	Sum Sq	DF	F value	p value
(Intercept)	143.13	1	34.89	<0.01
Diabetes	1.11	1	0.27	0.61
Final outcome	6.88	1	1.68	0.2
Residuals	143.59	35		

1.56) Resistin(BL) with Diabetes

1.56.1) ANOVA

	ANOVA type: **III**			
	Sum Sq	DF	F value	p value
(Intercept)	13522419270	1	209	<0.01
Diabetes	197059892.5	1	3.05	0.09
Final outcome	2295590.71	1	0.04	0.85
Residuals	2264470969	35		

1.57) Adiponectin/leptin(BL)(winsorized) with Diabetes

1.57.1) ANOVA

	ANOVA type: **III**			
	Sum Sq	DF	F value	p value
(Intercept)	5079360.91	1	10.13	<0.01
Diabetes	12246.25	1	0.024	0.88
Final outcome	4106.92	1	0.008	0.93
Residuals	17549062.58	35		

1.58) Adiponectin/Resistin(AT F1)(winsorized) with Diabete

1.58.1) ANOVA

	ANOVA type: III			
	Sum Sq	DF	F value	p value
(Intercept)	5058.78	1	7.55	<0.01

Diabetes	171.41	1	0.26	0.62
Final outcome	1003.64	1	1.5	0.23
Residuals	23451.55	35		

1.59) Leptin(AT)(winsorized) with Diabetes

1.59.1) ANOVA

	ANOVA type: III			
	Sum Sq	DF	F value	p value
(Intercept)	35984852.02	1	18.36	<0.01
Diabetes	781329.3	1	0.4	0.53
Final outcome	721938.3	1	0.37	0.55
Residuals	68592125.01	35		

1.60) Resistin(AT)(winsorized) with Diabetes

1.60.1) ANOVA

	ANOVA type: III			
	Sum Sq	DF	F value	p value
(Intercept)	12162388.44	1	12.06	<0.01
Diabetes	1645556.03	1	1.63	0.21
Final outcome	1562116.94	1	1.55	0.22
Residuals	35288687.43	35		

1.61) Adiponectin(BL) with Age

1.61.1) ANOVA

	ANOVA type: III			
	Sum Sq	DF	F value	p value
(Intercept)	9.34834E+13	1	7.16	0.01
Age	2.90919E+12	1	0.22	0.64
Final outcome	1.2727E+12	1	0.1	0.76
Residuals	4.56698E+14	35		

1.62) Adiponectin(FI AT)(winsorized) with Age

1.62.1) ANOVA

	ANOVA type: III			
	Sum Sq	DF	F value	p value
(Intercept)	10533398.61	1	6.54	0.02
Age	3186204	1	1.98	0.17
Final outcome	198220.47	1	0.12	0.73
Residuals	56390036.5	35		

1.63) Adiponectin/Leptin(AT FI)(winsorized) with Age

1.63.1) ANOVA

	ANOVA type: III			
	Sum Sq	DF	F value	p value
(Intercept)	662.75	1	1.84	0.18
Age	67.96	1	0.19	0.67
Final outcome	261.13	1	0.73	0.4
Residuals	12589.79	35		

1.64) Adiponectin/Resistin(BL) with Age

1.64.1) ANOVA

	ANOVA type: III			
	Sum Sq	DF	F value	p value
(Intercept)	284419.61	1	3.986	0.05
Age	689.81	1	0.01	0.92
Final outcome	1935.03	1	0.027	0.87
Residuals	2497409.95	35		

1.65) Leptin(BL) with Age

1.65.1) ANOVA

	ANOVA type: III			
	Sum Sq	DF	F value	p value
(Intercept)	6669566060	1	2.89	0.1
Age	302168577.2	1	0.13	0.72
Final outcome	3592929083	1	1.56	0.22
Residuals	80837200508	35		

1.66) Leptin/Resistin(AT)(winsorized) with Age

1.66.1) ANOVA

	ANOVA type: III			
	Sum Sq	DF	F value	p value
(Intercept)	117.94	1	5.1	0.03
Age	21.93	1	0.95	0.34
Final outcome	124.52	1	5.39	0.03
Residuals	809.15	35		

1.67) leptin/Resistin(BL) with Age

1.67.1) ANOVA

	ANOVA type: III			
	Sum Sq	DF	F value	p value

(Intercept)	18.92	1	4.65	0.04
Age	2.23	1	0.55	0.46
Final outcome	7.73	1	1.9	0.18
Residuals	142.47	35		

1.68) Resistin(BL) with Age

1.68.1) ANOVA

	ANOVA type: III			
	Sum Sq	DF	F value	p value
(Intercept)	666584372.3	1	9.478	<0.01
Age	115880.5	1	0.002	0.97
Final outcome	10356996.22	1	0.147	0.7
Residuals	2461414981	35		

1.69) Adiponectin/leptin(BL)(winsorized) with Age

1.69.1) ANOVA

	ANOVA type: III			
	Sum Sq	DF	F value	p value
(Intercept)	230817.95	1	0.46	0.5
Age	3442.47	1	0.007	0.93
Final outcome	8206.89	1	0.016	0.9
Residuals	17557866.36	35		

1.70) Adiponectin/Resistin(AT F1)(winsorized) with Age

1.70.1) ANOVA

	ANOVA type: III			
	Sum Sq	DF	F value	p value
(Intercept)	141.25	1	0.21	0.65
Age	14.75	1	0.02	0.88
Final outcome	918.23	1	1.36	0.25
Residuals	23608.21	35		

1.71) Leptin(AT)(winsorized) with Age

1.71.1) ANOVA

	ANOVA type: III			
	Sum Sq	DF	F value	p value
(Intercept)	8925133.61	1	4.7	0.04
Age	2866193.62	1	1.51	0.23
Final outcome	111407.47	1	0.06	0.81
Residuals	66507260.68	35		

1.72) Resistin(AT)(winsorized) with Age

1.72.1) ANOVA

	ANOVA type: III			
	Sum Sq	DF	F value	p value
(Intercept)	769759.69	1	0.73	0.4
Age	27771.83	1	0.03	0.87
Final outcome	1048774.88	1	0.99	0.33
Residuals	36906471.63	35		

y

sion

n

ension

es

1) R lm

1.1) Adiponectin/Resistin(AT F1)(winsorized) with Obesity

1.1.1) ANOVA

	ANOVA type: III			
	Sum Sq	DF	F value	p value
(Intercept)	4794.51	1	7.11	0.01
Obesity	6.9	1	0.01	0.92
Final outcome	916.42	1	1.36	0.25
Residuals	23616.06	35		

1.1.2) Levene's test

	Levene's test for homogeneity of variance	
	F test	p-value
Obesity	F(1, 36)=0.01	0.91
Final outcome	F(1, 36)=7.59	<0.01

1.1.3) Final outcome LS means graph

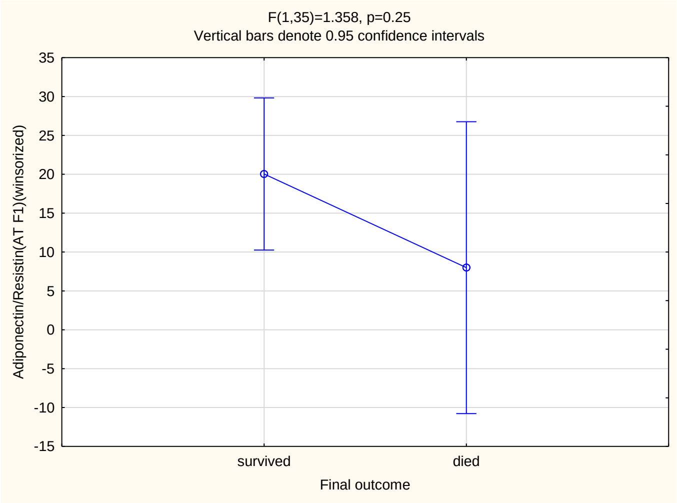

1.1.4) Welch tests

1.1.4.1) Final outcome adjusted means plot

1.2) Adiponectin/Resistin(AT F1)(winsorized) with HIV

1.2.1) ANOVA

	ANOVA type: III			
	Sum Sq	DF	F value	p value
(Intercept)	5140.24	1	7.65	<0.01
HIV	113.84	1	0.17	0.68
Final outcome	899.71	1	1.34	0.25
Residuals	23509.12	35		

1.2.2) Levene's test

	Levene's test for homogeneity of variance	
	F test	p-value
HIV	F(1, 36)=0.43	0.51
Final outcome	F(1, 36)=7.59	<0.01

1.2.3) Final outcome LS means graph

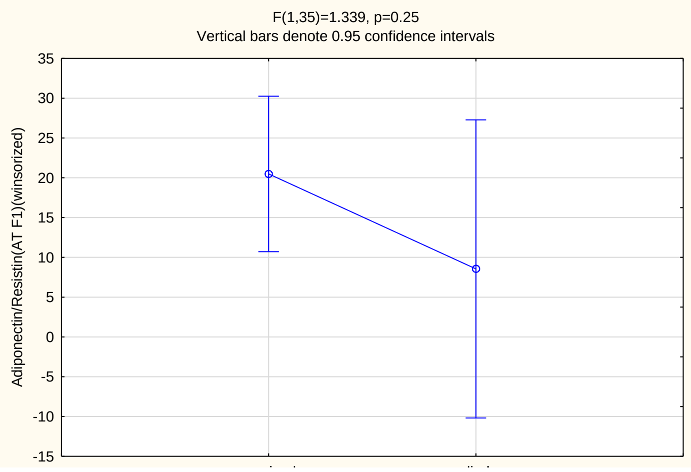

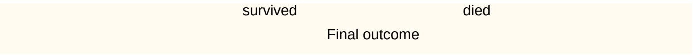

1.2.4) Welch tests

1.2.4.1) Final outcome adjusted means plot

1.3) Adiponectin/Resistin(AT F1)(winsorized) with severity

1.3.1) ANOVA

	ANOVA type: III			
	Sum Sq	DF	F value	p value
(Intercept)	3447.78	1	5.11	0.03
severity	15.54	1	0.02	0.88
Final outcome	890.1	1	1.32	0.26
Residuals	23607.42	35		

1.3.2) Levene's test

	Levene's test for homogeneity of variance	
	F test	p-value
severity	F(1, 36)=0.35	0.56
Final outcome	F(1, 36)=7.59	<0.01

1.3.3) Final outcome LS means graph

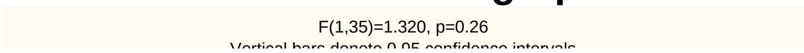

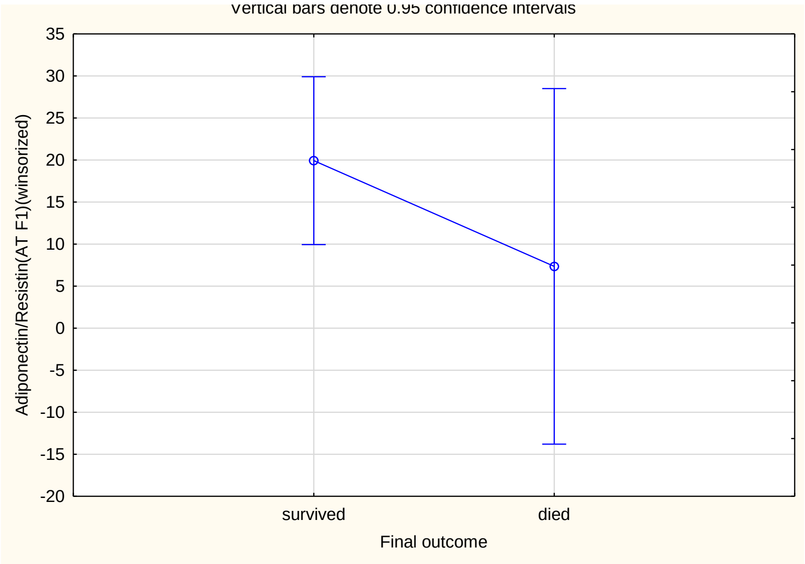

1.3.4) Welch tests

1.3.4.1) Final outcome adjusted means plot

1.4) Adiponectin/Resistin(AT F1)(winsorized) with Sex

1.4.1) ANOVA

	ANOVA type: III			
	Sum Sq	DF	F value	p value
(Intercept)	3421.86	1	5.08	0.03
Sex	27.03	1	0.04	0.84
Final outcome	939.44	1	1.39	0.25
Residuals	23595.94	35		

1.4.2) Levene's test

	Levene's test for homogeneity of variance	
	F test	p-value
Sex	F(1, 36)=0.00	0.95
Final outcome	F(1, 36)=7.59	<0.01

1.4.3) Final outcome LS means graph

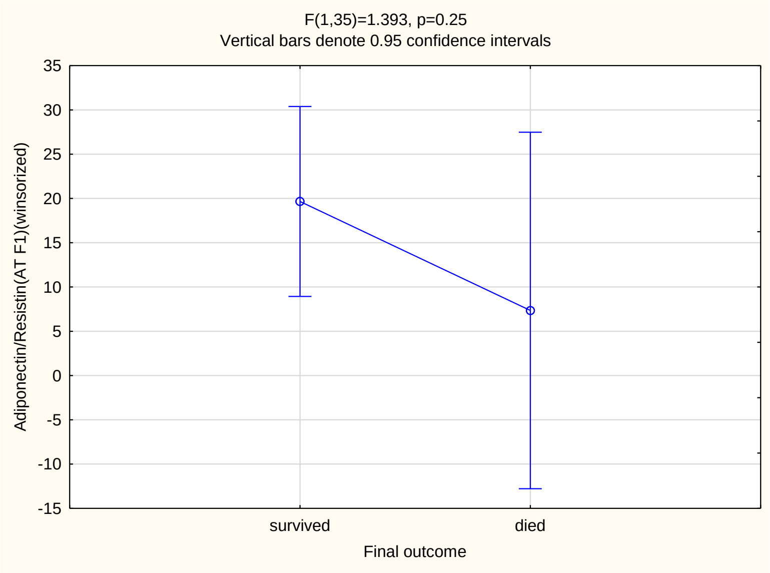

1.4.4) Welch tests

1.4.4.1) Final outcome adjusted means plot

1.5) Adiponectin/Resistin(AT F1)(winsorized) with Hyperte

1.5.1) ANOVA

	ANOVA type: III			
	Sum Sq	DF	F value	p value
(Intercept)	4483.78	1	6.65	0.01
Hypertension	37.36	1	0.06	0.82
Final outcome	948.85	1	1.41	0.24
Residuals	23585.6	35		

1.5.2) Levene's test

	Levene's test for homogeneity of variance	
	F test	p-value
Hypertension	F(1, 36)=0.98	0.33
Final outcome	F(1, 36)=7.59	<0.01

1.5.3) Final outcome LS means graph

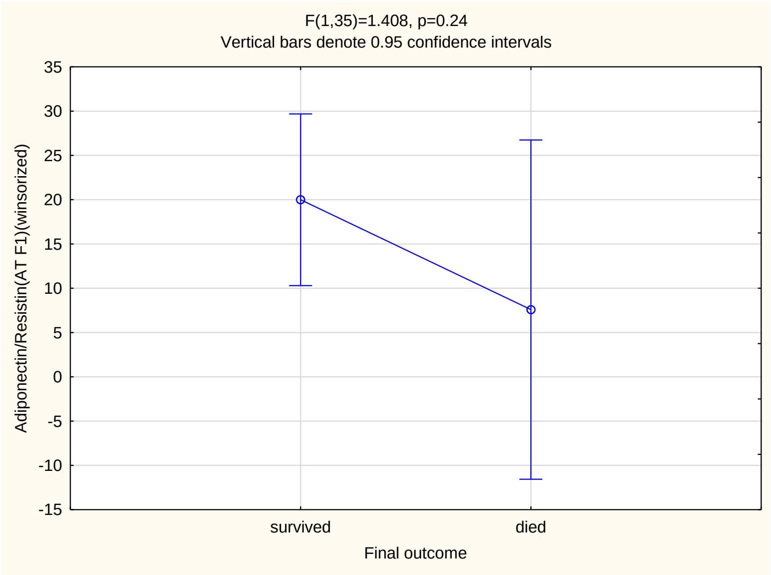

1.5.4) Welch tests

1.5.4.1) Final outcome adjusted means plot

1.6) Adiponectin/Resistin(AT F1)(winsorized) with Diabete[illegible]

1.6.1) ANOVA

	ANOVA type: III			
	Sum Sq	DF	F value	p value
(Intercept)	5058.78	1	7.55	<0.01
Diabetes	171.41	1	0.26	0.62
Final outcome	1003.64	1	1.5	0.23
Residuals	23451.55	35		

1.6.2) Levene's test

	Levene's test for homogeneity of variance	
	F test	p-value
Diabetes	F(1, 36)=0.06	0.81
Final outcome	F(1, 36)=7.59	<0.01

1.6.3) Final outcome LS means graph

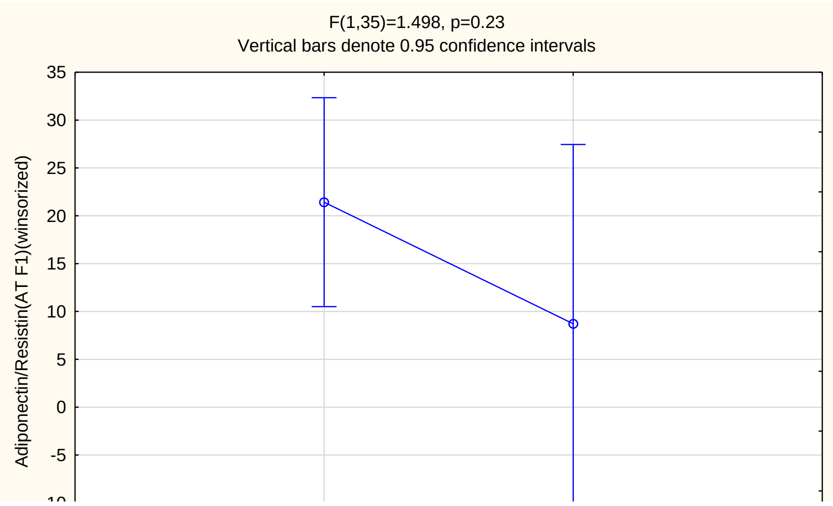

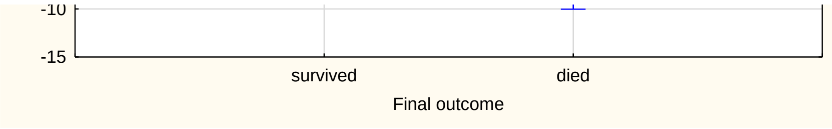

1.6.4) Welch tests

1.6.4.1) Final outcome adjusted means plot

1.7) Adiponectin/Resistin(AT F1)(winsorized) with Age

1.7.1) ANOVA

	ANOVA type: III			
	Sum Sq	DF	F value	p value
(Intercept)	141.25	1	0.21	0.65
Age	14.75	1	0.02	0.88
Final outcome	918.23	1	1.36	0.25
Residuals	23608.21	35		

1.7.2) Levene's test

	Levene's test for homogeneity of variance	
	F test	p-value
Final outcome	F(1, 36)=7.59	<0.01

1.7.3) Final outcome LS means graph

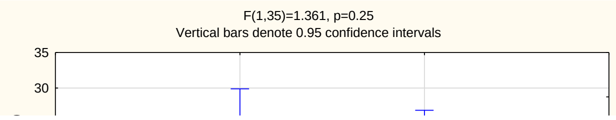

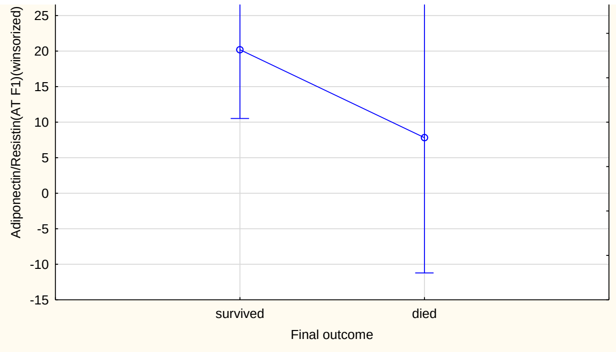

1.7.4) Welch tests

1.7.4.1) Final outcome adjusted means plot

2) R lm

2.1) IL2 (AT) with Obesity

2.1.1) ANOVA

	ANOVA type: III			
	Sum Sq	DF	F value	p value
(Intercept)	122579.88	1	17.81	<0.01
Obesity	4105.65	1	0.6	0.45
Final outcome	12445.98	1	1.81	0.19
Residuals	240896.54	35		

2.1.2) Levene's test

	Levene's test for homogeneity of variance	
	F test	p-value
Obesity	F(1, 36)=0.18	0.67
Final outcome	F(1, 36)=3.05	0.09

2.1.3) Final outcome LS means graph

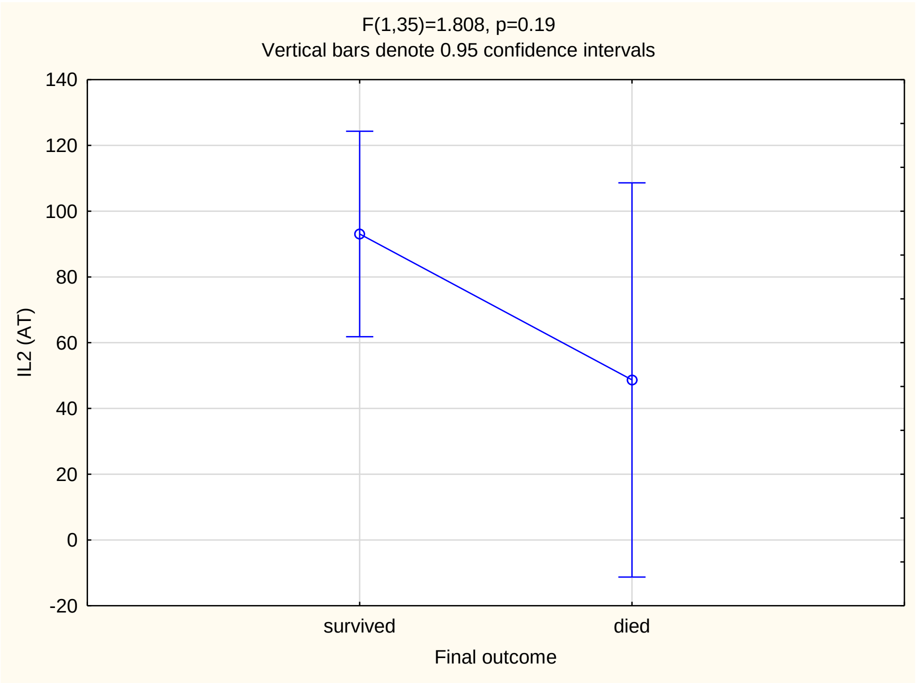

2.2) IL10(AT) with Obesity

2.2.1) ANOVA

	ANOVA type: III			
	Sum Sq	DF	F value	p value
(Intercept)	16.27	1	23.17	<0.01
Obesity	0.74	1	1.05	0.31
Final outcome	0.93	1	1.32	0.26
Residuals	24.58	35		

2.2.2) Levene's test

	Levene's test for homogeneity of variance	
	F test	p-value
Obesity	F(1, 36)=0.88	0.35
Final outcome	F(1, 36)=2.08	0.16

2.2.3) Final outcome LS means graph

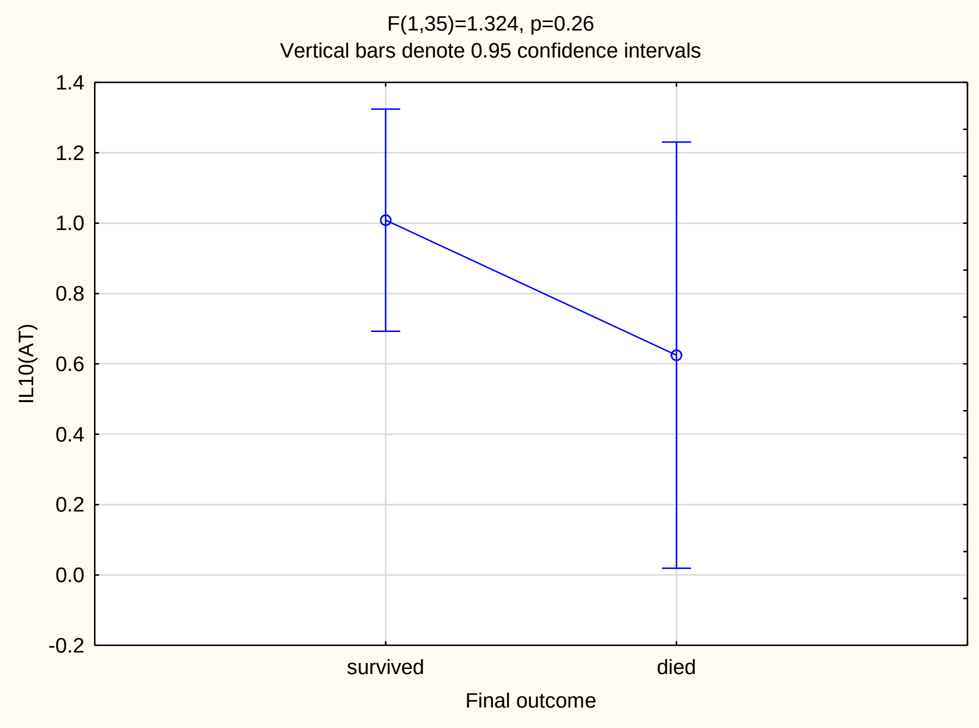

2.3) MCP1(BL) with Obesity

2.3.1) ANOVA

	ANOVA type: III			
	Sum Sq	DF	F value	p value
(Intercept)	6570229.83	1	40.88	<0.01
Obesity	273960.7	1	1.7	0.2
Final outcome	46158.7	1	0.29	0.6
Residuals	5625037.1	35		

2.3.2) Levene's test

	Levene's test for homogeneity of variance	
	F test	p-value
Obesity	F(1, 36)=2.66	0.11
Final outcome	F(1, 36)=0.44	0.51

2.3.3) Final outcome LS means graph

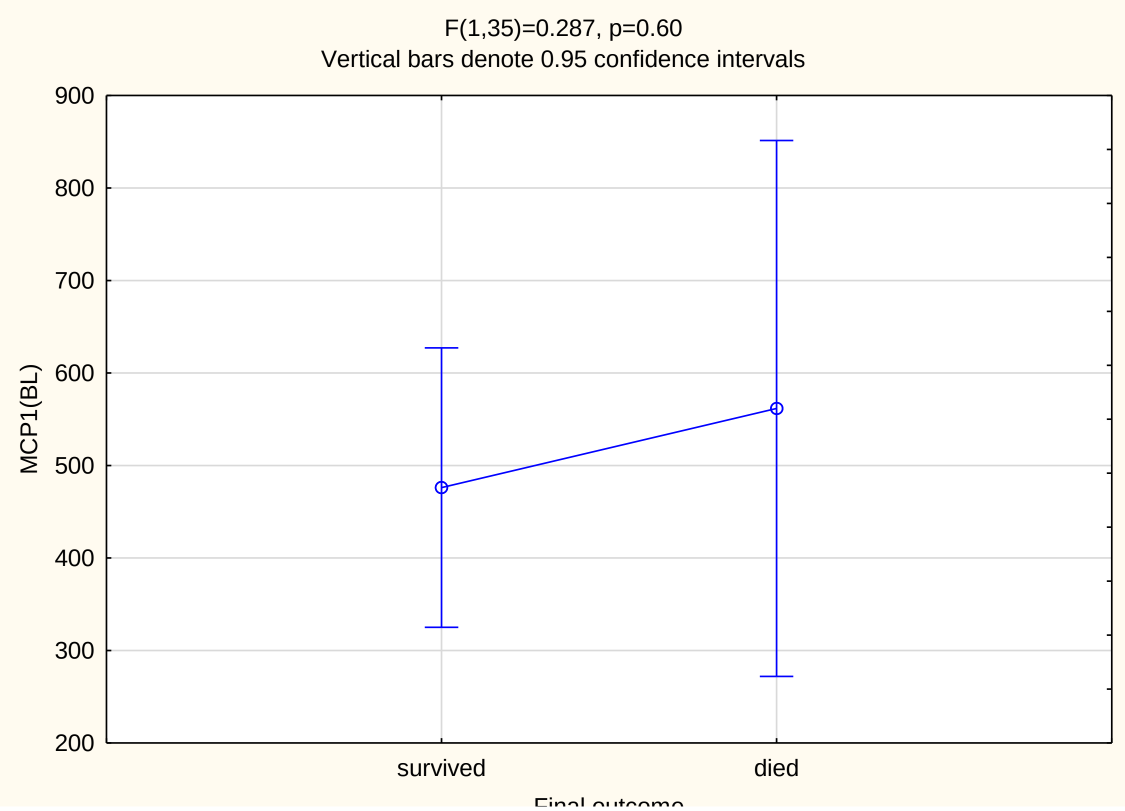

2.4) IL6(BL) with Obesity

2.4.1) ANOVA

	ANOVA type: III			
	Sum Sq	DF	F value	p value
(Intercept)	5122.27	1	35.31	<0.01
Obesity	56.52	1	0.39	0.54
Final outcome	179.22	1	1.24	0.27
Residuals	5077.74	35		

2.4.2) Levene's test

	Levene's test for homogeneity of variance	
	F test	p-value
Obesity	F(1, 36)=0.75	0.39
Final outcome	F(1, 36)=0.60	0.44

2.4.3) Final outcome LS means graph

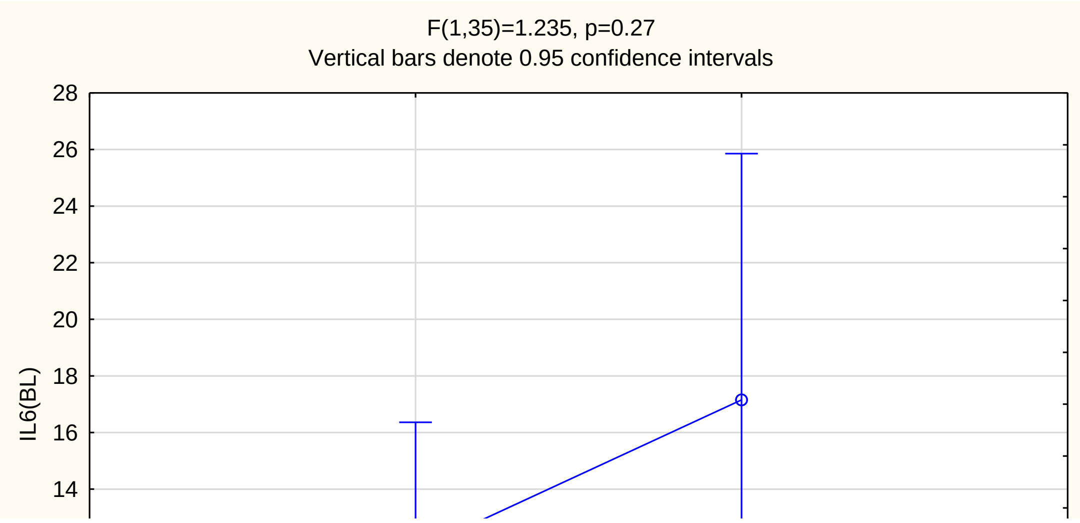

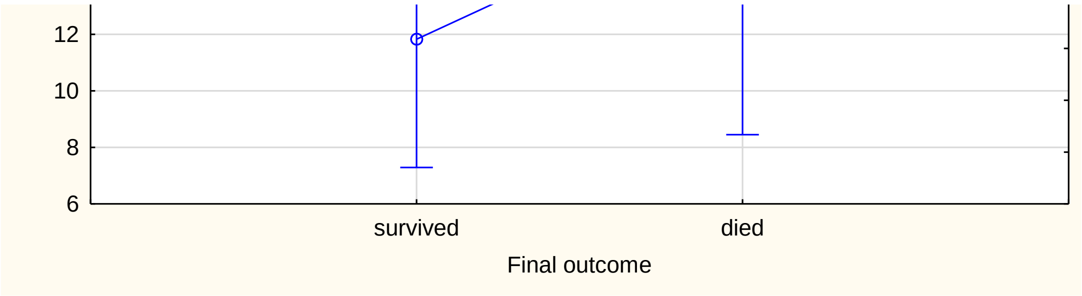

2.5) IL2(BL) with Obesity

2.5.1) ANOVA

	ANOVA type: III			
	Sum Sq	DF	F value	p value
(Intercept)	505587.58	1	256.392	<0.01
Obesity	711.83	1	0.361	0.55
Final outcome	5.1	1	0.003	0.96
Residuals	69017.55	35		

2.5.2) Levene's test

	Levene's test for homogeneity of variance	
	F test	p-value
Obesity	F(1, 36)=0.44	0.51
Final outcome	F(1, 36)=0.79	0.38

2.5.3) Final outcome LS means graph

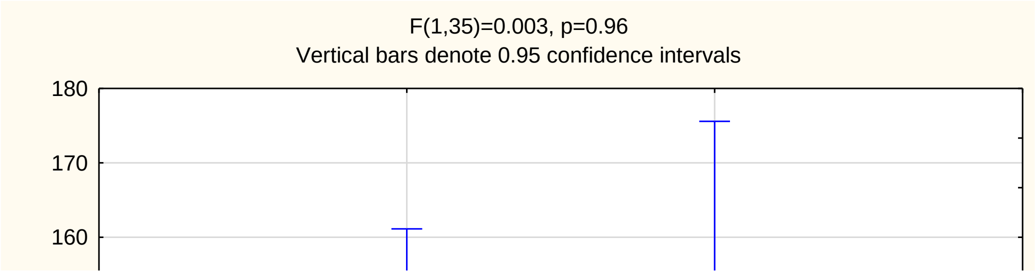

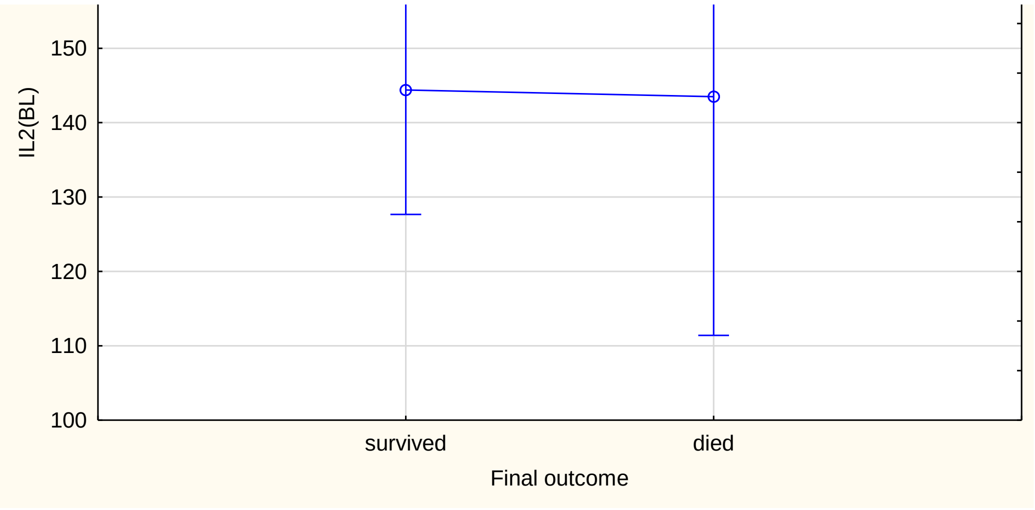

2.6) IL10(BL) with Obesity

2.6.1) ANOVA

	ANOVA type: III			
	Sum Sq	DF	F value	p value
(Intercept)	860.75	1	68.392	<0.01
Obesity	0.02	1	0.001	0.97
Final outcome	58.69	1	4.663	0.04
Residuals	440.49	35		

2.6.2) Levene's test

	Levene's test for homogeneity of variance	
	F test	p-value
Obesity	F(1, 36)=3.68	0.06
Final outcome	F(1, 36)=8.59	<0.01

2.6.3) Final outcome LS means graph

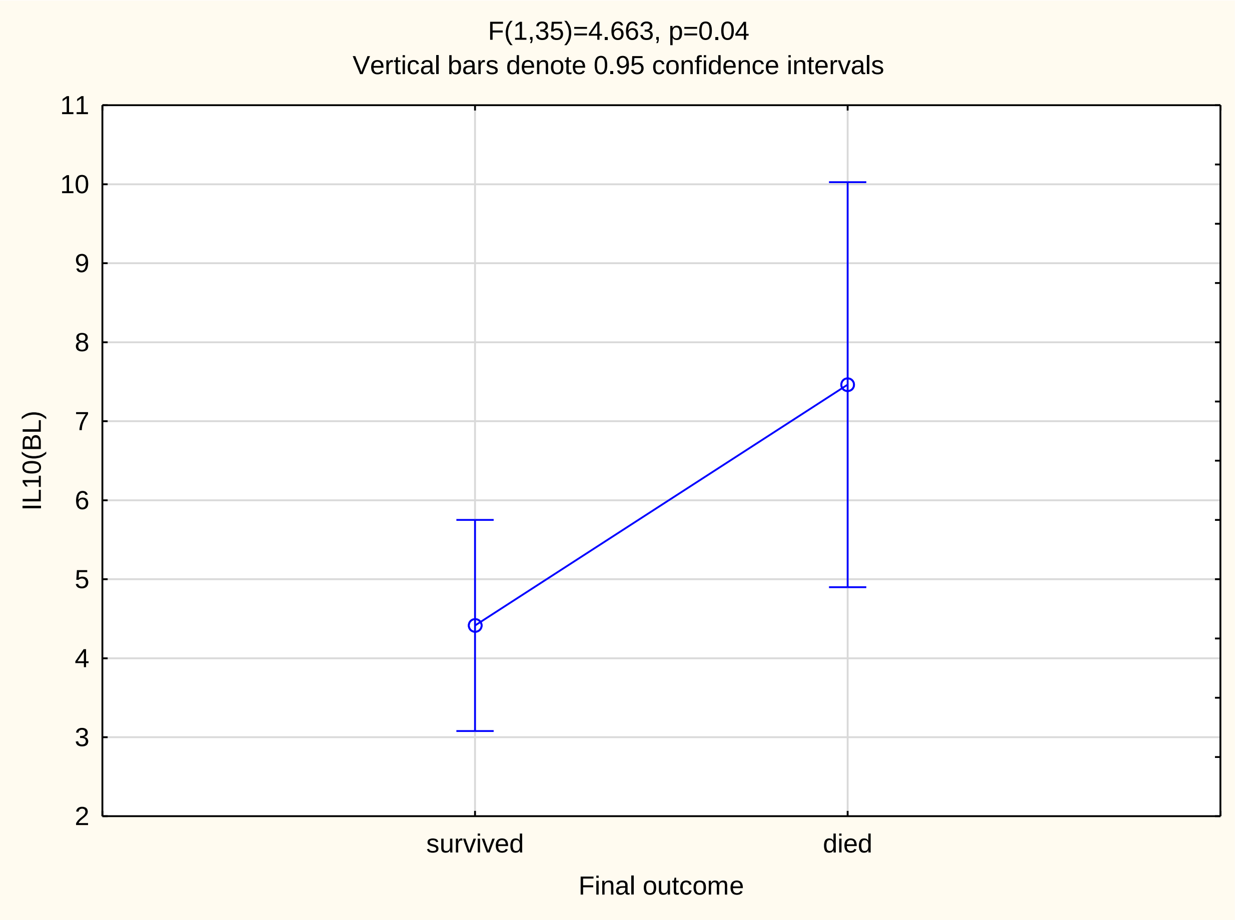

2.6.4) Welch tests

2.6.4.1) Final outcome adjusted means plot

2.7) TNFa(BL) with Obesity

2.7.1) ANOVA

	ANOVA type: III			
	Sum Sq	DF	F value	p value
(Intercept)	1885.75	1	81.018	<0.01
Obesity	30.52	1	1.311	0.26
Final outcome	0.1	1	0.004	0.95
Residuals	814.65	35		

2.7.2) Levene's test

	Levene's test for homogeneity of variance	
	F test	p-value
Obesity	F(1, 36)=1.40	0.24
Final outcome	F(1, 36)=0.58	0.45

2.7.3) Final outcome LS means graph

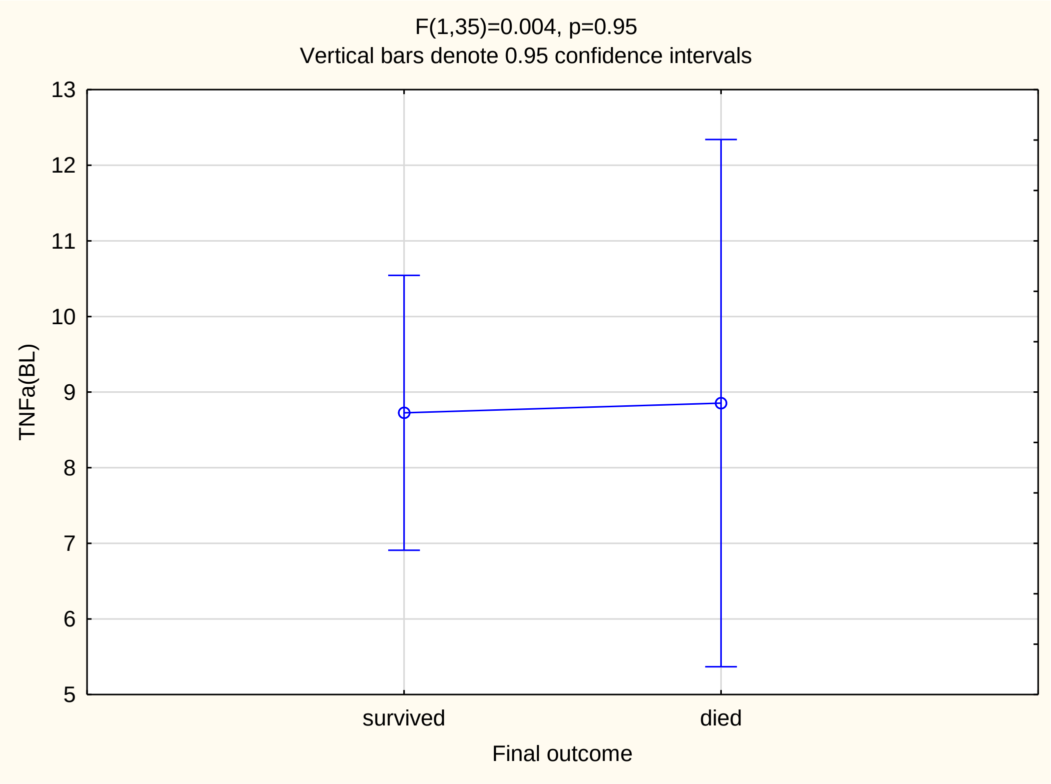

2.8) TNFa(AT)(winsorized) with Obesity

2.8.1) ANOVA

	ANOVA type: III			
	Sum Sq	DF	F value	p value
(Intercept)	2.12	1	20.65	<0.01
Obesity	0.21	1	2.03	0.16
Final outcome	0.21	1	2.05	0.16
Residuals	3.59	35		

2.8.2) Levene's test

	Levene's test for homogeneity of variance	
	F test	p-value
Obesity	F(1, 36)=1.42	0.24
Final outcome	F(1, 36)=4.89	0.03

2.8.3) Final outcome LS means graph

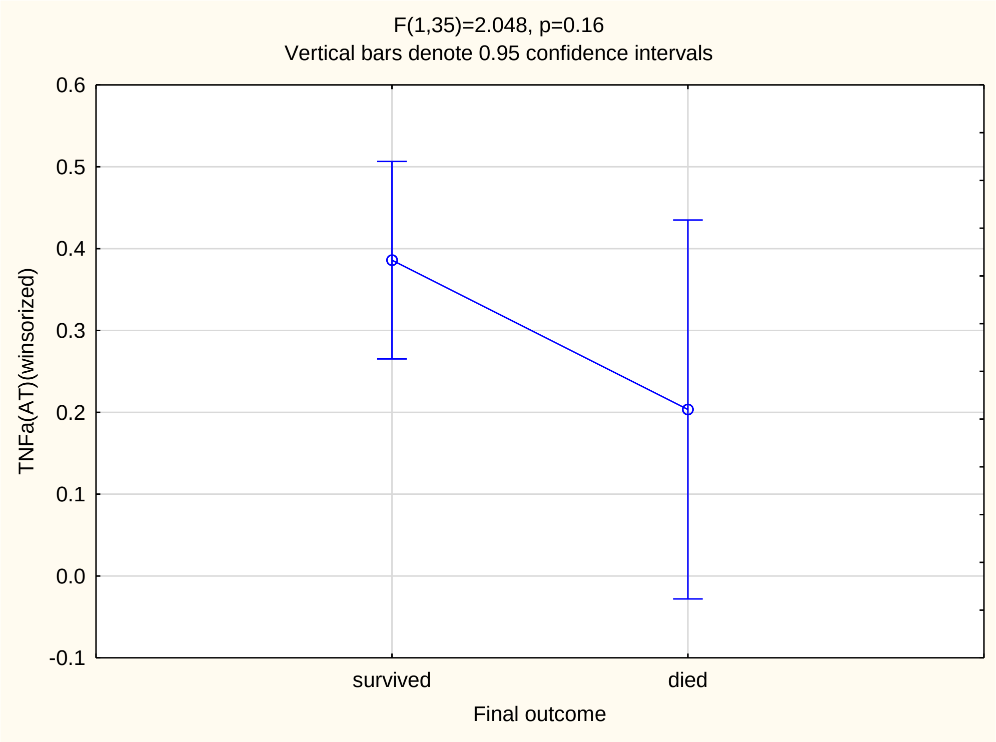

2.9) MCP1(AT)(winsorized) with Obesity

2.9.1) ANOVA

	ANOVA type: III			
	Sum Sq	DF	F value	p value
(Intercept)	3289.05	1	21.96	<0.01
Obesity	65.02	1	0.43	0.51
Final outcome	197.47	1	1.32	0.26
Residuals	5241.31	35		

2.9.2) Levene's test

	Levene's test for homogeneity of variance	
	F test	p-value
Obesity	F(1, 36)=0.46	0.5
Final outcome	F(1, 36)=0.93	0.34

2.9.3) Final outcome LS means graph

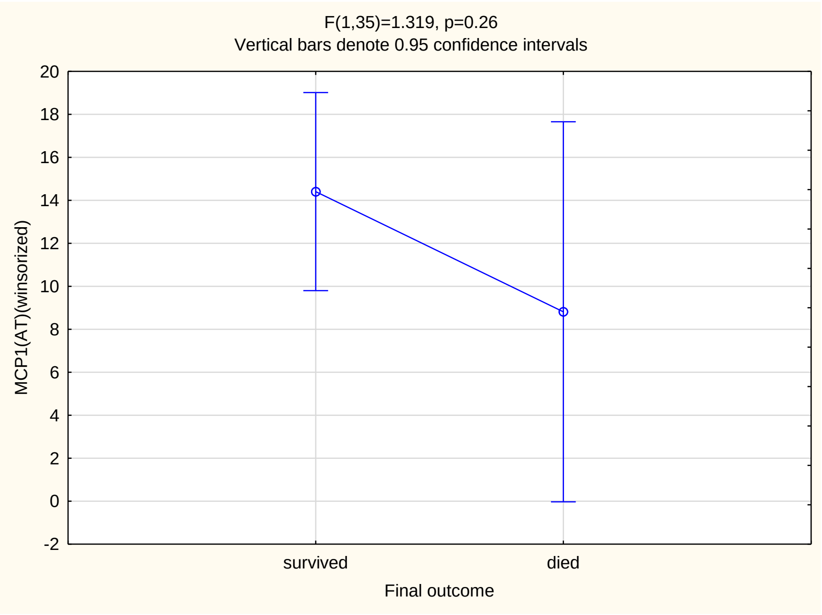

2.10) IL6(AT)(winsorized) with Obesity

2.10.1) ANOVA

	ANOVA type: III			
	Sum Sq	DF	F value	p value
(Intercept)	29.81	1	20.05	<0.01
Obesity	0.92	1	0.62	0.44
Final outcome	2.07	1	1.39	0.25
Residuals	52.02	35		

2.10.2) Levene's test

	Levene's test for homogeneity of variance	
	F test	p-value
Obesity	F(1, 36)=0.46	0.5
Final outcome	F(1, 36)=2.13	0.15

2.10.3) Final outcome LS means graph

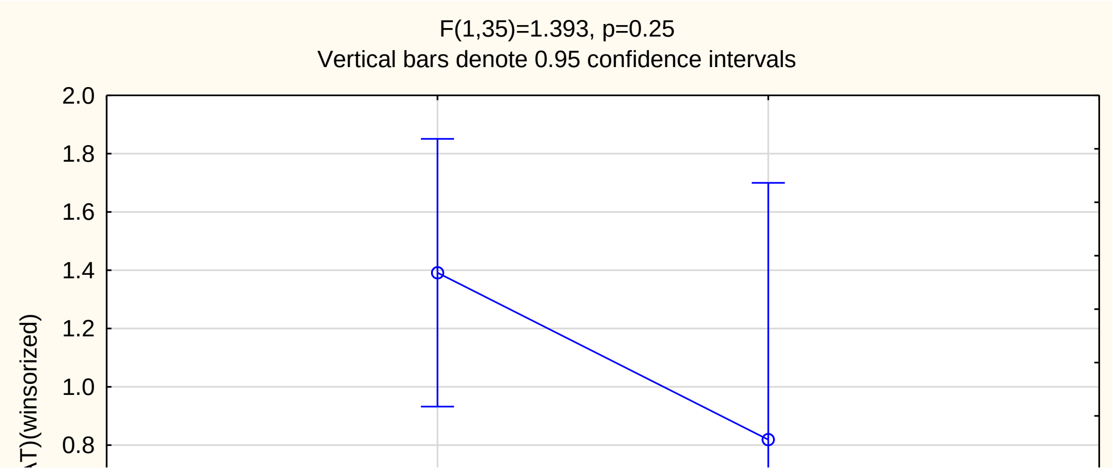

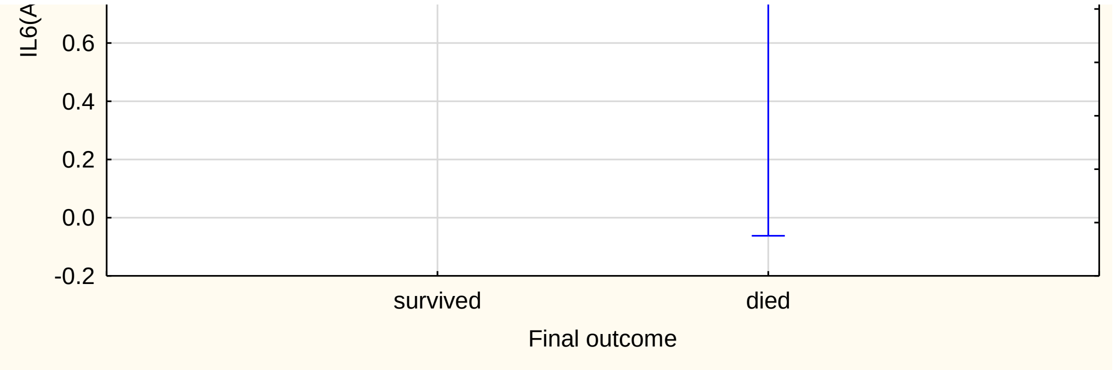

2.11) IL17 (AT)(winsorized) with Obesity

2.11.1) ANOVA

	ANOVA type: III			
	Sum Sq	DF	F value	p value
(Intercept)	6.75	1	30.04	<0.01
Obesity	0.19	1	0.84	0.37
Final outcome	0.37	1	1.64	0.21
Residuals	7.86	35		

2.11.2) Levene's test

	Levene's test for homogeneity of variance	
	F test	p-value
Obesity	F(1, 36)=0.45	0.51
Final outcome	F(1, 36)=1.61	0.21

2.11.3) Final outcome LS means graph

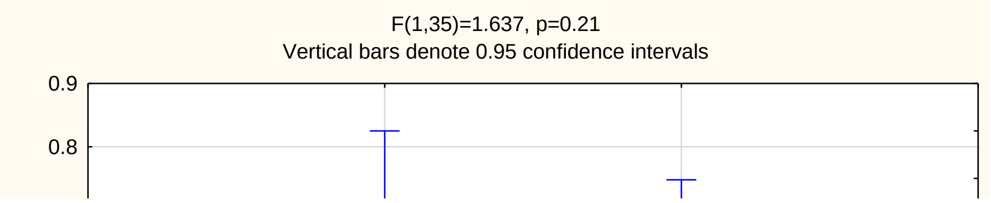

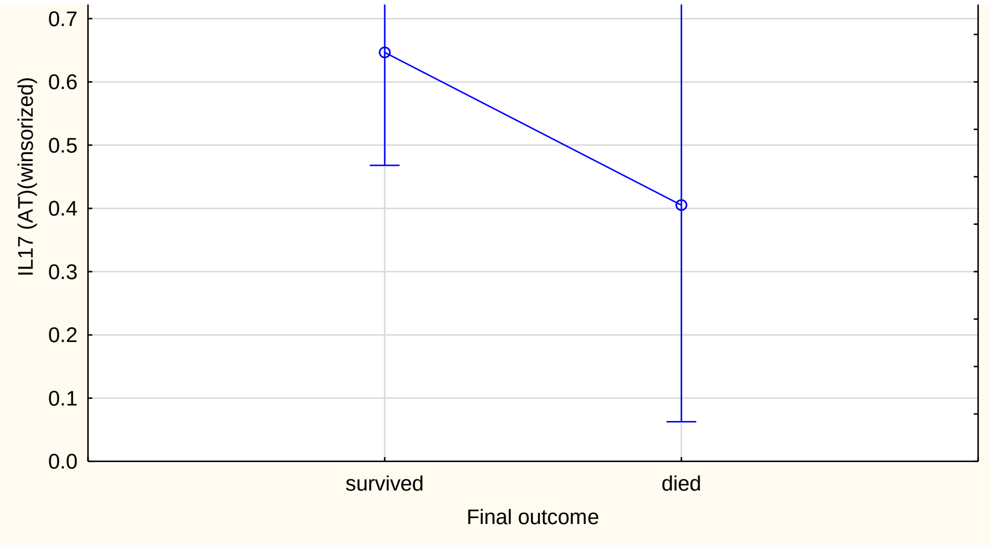

2.12) IL1b(BL)(winsorized) with Obesity

2.12.1) ANOVA

	ANOVA type: III			
	Sum Sq	DF	F value	p value
(Intercept)	692.09	1	2392.37	<0.01
Obesity	1.37	1	4.73	0.04
Final outcome	0.02	1	0.07	0.8
Residuals	10.13	35		

2.12.2) Levene's test

	Levene's test for homogeneity of variance	
	F test	p-value
Obesity	F(1, 36)=3.42	0.07
Final outcome	F(1, 36)=2.31	0.14

2.12.3) Final outcome LS means graph

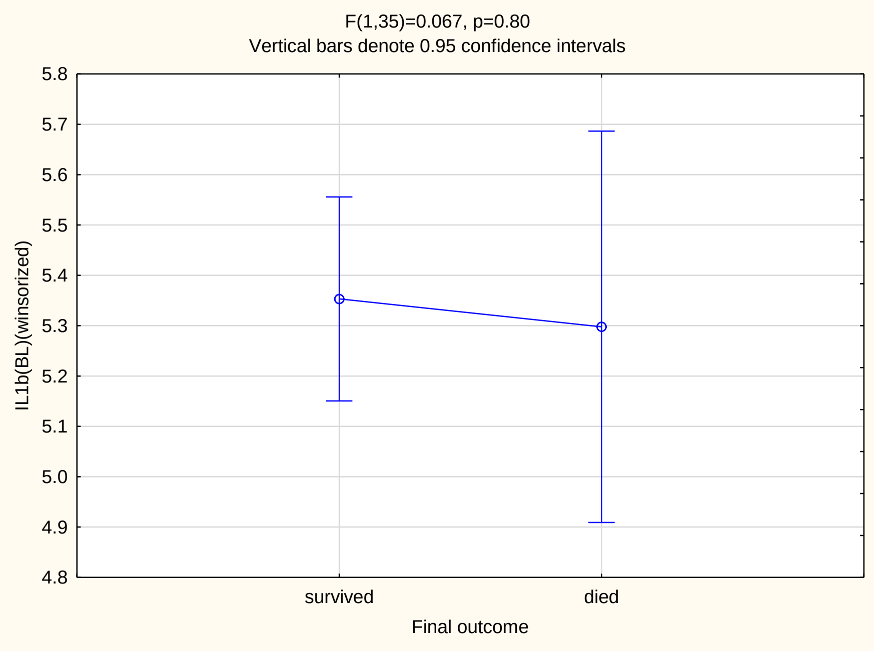

2.13) IL17(BL)(winsorized) with Obesity

2.13.1) ANOVA

	ANOVA type: III			
	Sum Sq	DF	F value	p value
(Intercept)	174.08	1	23.62	<0.01
Obesity	7.88	1	1.07	0.31
Final outcome	0.2	1	0.03	0.87
Residuals	257.99	35		

2.13.2) Levene's test

	Levene's test for homogeneity of variance	
	F test	p-value
Obesity	F(1, 36)=0.76	0.39
Final outcome	F(1, 36)=0.07	0.8

2.13.3) Final outcome LS means graph

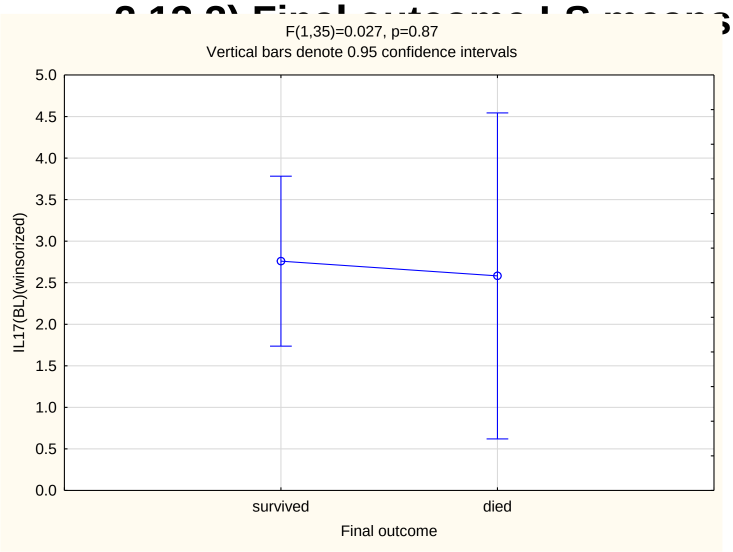

2.14) IL1b(FI AT)(winsorized) with Obesity

2.14.1) ANOVA

	ANOVA type: III			
	Sum Sq	DF	F value	p value
(Intercept)	4.67	1	17.9	<0.01
Obesity	0.45	1	1.72	0.2
Final outcome	0.89	1	3.41	0.07
Residuals	9.14	35		

2.14.2) Levene's test

	Levene's test for homogeneity of variance	
	F test	p-value
Obesity	F(1, 36)=1.51	0.23
Final outcome	F(1, 36)=4.68	0.04

2.14.3) Final outcome LS means graph

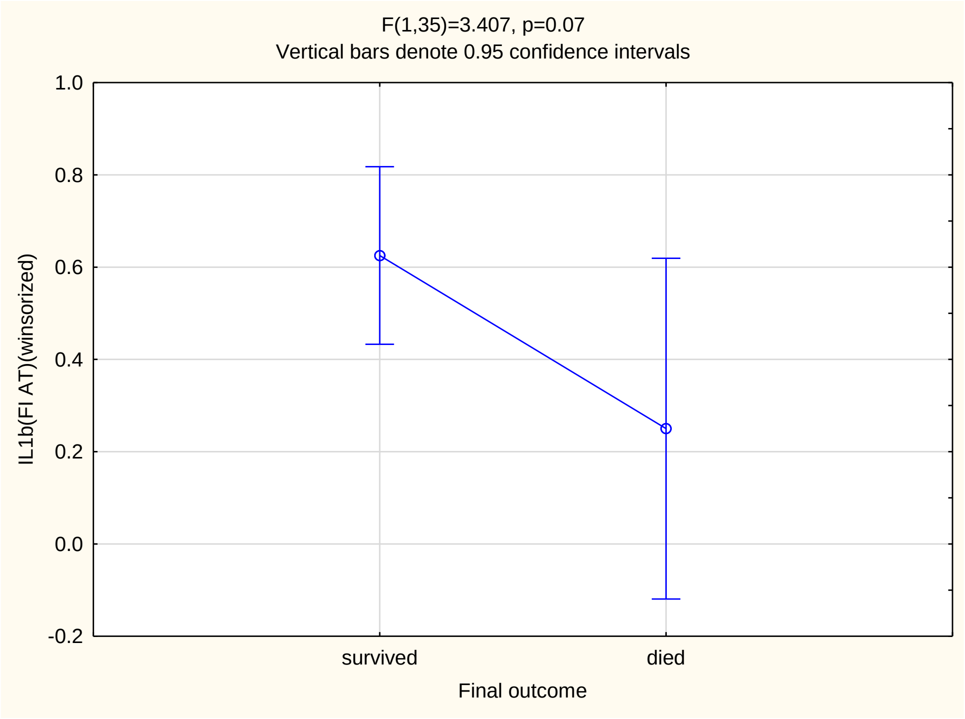

2.15) IL2 (AT) with HIV

2.15.1) ANOVA

	ANOVA type: III			
	Sum Sq	DF	F value	p value
(Intercept)	133078.38	1	19.03	<0.01
HIV	241.05	1	0.03	0.85
Final outcome	12078.39	1	1.73	0.2
Residuals	244761.14	35		

2.15.2) Levene's test

	Levene's test for homogeneity of variance	
	F test	p-value
HIV	F(1, 36)=0.35	0.56
Final outcome	F(1, 36)=3.05	0.09

2.15.3) Final outcome LS means graph

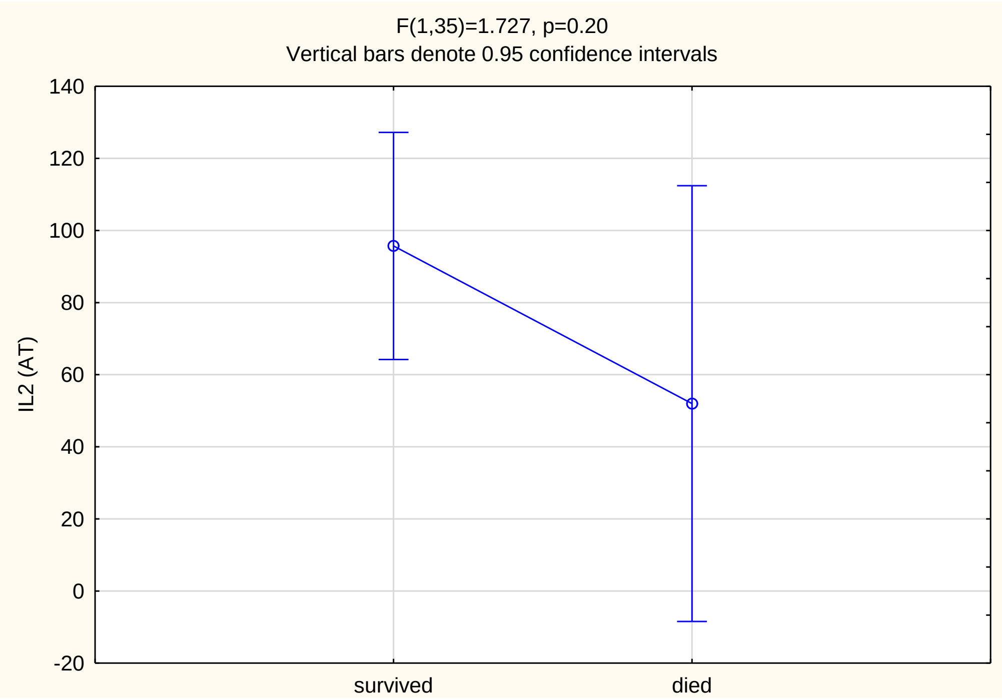

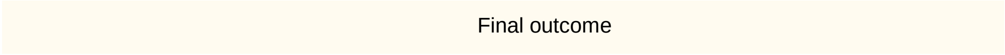

2.16) IL10(AT) with HIV

2.16.1) ANOVA

	ANOVA type: III			
	Sum Sq	DF	F value	p value
(Intercept)	17.74	1	24.54	<0.01
HIV	0.01	1	0.02	0.9
Final outcome	0.89	1	1.23	0.27
Residuals	25.3	35		

2.16.2) Levene's test

	Levene's test for homogeneity of variance	
	F test	p-value
HIV	F(1, 36)=0.70	0.41
Final outcome	F(1, 36)=2.08	0.16

2.16.3) Final outcome LS means graph

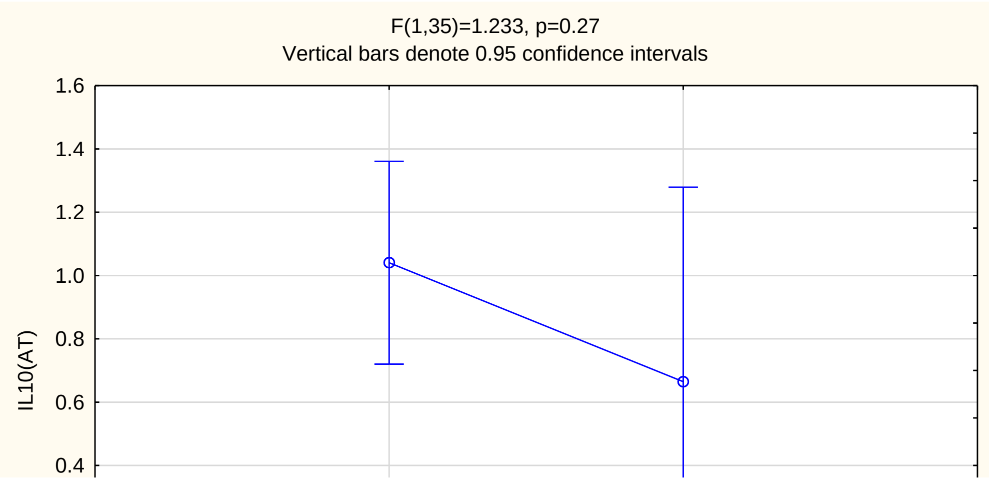

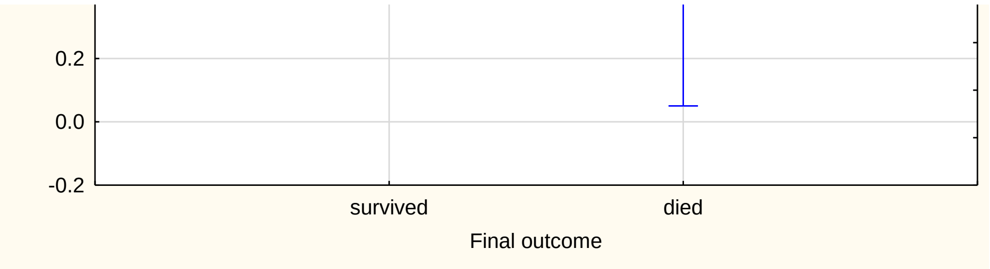

2.17) MCP1(BL) with HIV

2.17.1) ANOVA

	ANOVA type: III			
	Sum Sq	DF	F value	p value
(Intercept)	6461836.53	1	39.45	<0.01
HIV	166728.84	1	1.02	0.32
Final outcome	45133.3	1	0.28	0.6
Residuals	5732268.96	35		

2.17.2) Levene's test

	Levene's test for homogeneity of variance	
	F test	p-value
HIV	F(1, 36)=2.74	0.11
Final outcome	F(1, 36)=0.44	0.51

2.17.3) Final outcome LS means graph

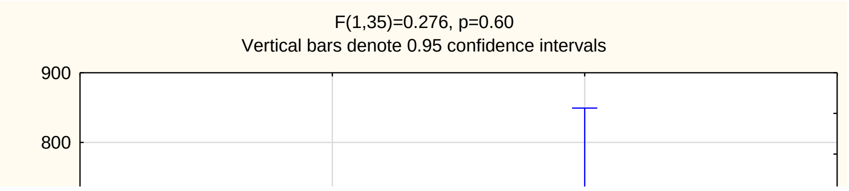

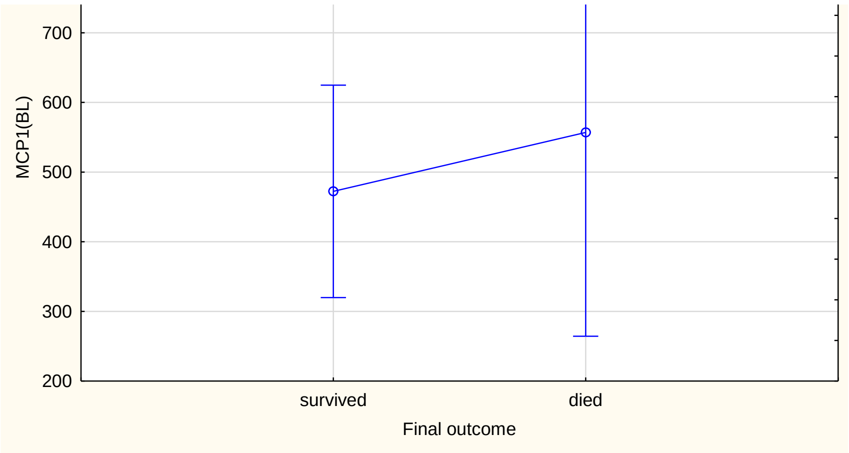

2.18) IL6(BL) with HIV

2.18.1) ANOVA

	ANOVA type: III			
	Sum Sq	DF	F value	p value
(Intercept)	5058.82	1	34.66	<0.01
HIV	26.09	1	0.18	0.68
Final outcome	177.87	1	1.22	0.28
Residuals	5108.17	35		

2.18.2) Levene's test

	Levene's test for homogeneity of variance	
	F test	p-value
HIV	F(1, 36)=1.62	0.21
Final outcome	F(1, 36)=0.60	0.44

2.18.3) Final outcome LS means graph

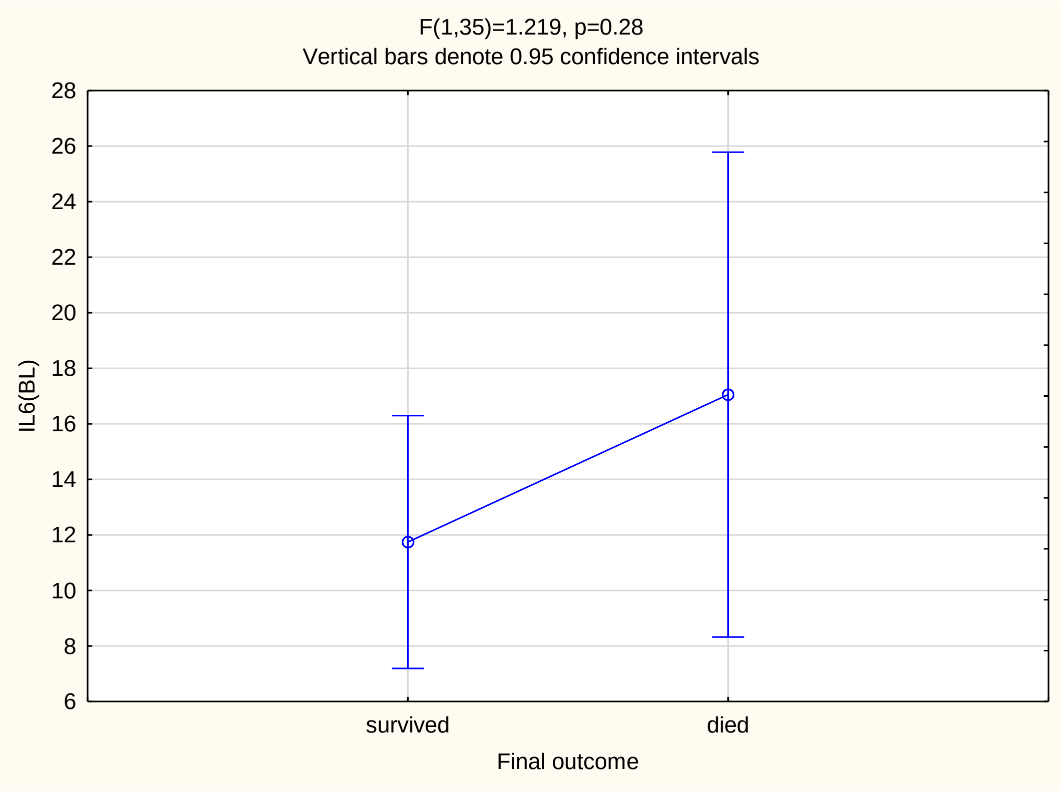

2.19) IL2(BL) with HIV

2.19.1) ANOVA

	ANOVA type: III			
	Sum Sq	DF	F value	p value
(Intercept)	492829.69	1	269.47	<0.01
HIV	5718.37	1	3.127	0.09
Final outcome	10.75	1	0.006	0.94
Residuals	64011.01	35		

2.19.2) Levene's test

	Levene's test for homogeneity of variance	
	F test	p-value
HIV	F(1, 36)=0.37	0.54
Final outcome	F(1, 36)=0.79	0.38

2.19.3) Final outcome LS means graph

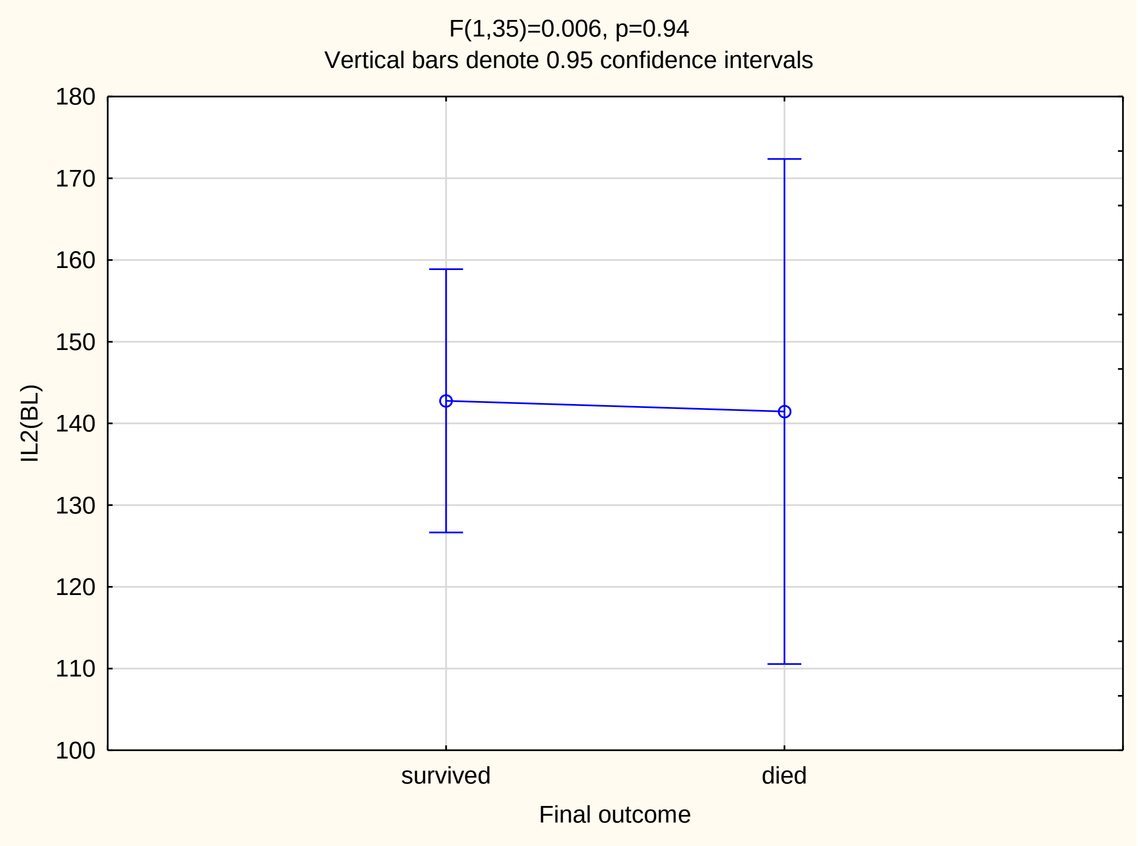

2.20) IL10(BL) with HIV

2.20.1) ANOVA

	ANOVA type: III			
	Sum Sq	DF	F value	p value

(Intercept)	875.18	1	69.76	<0.01
HIV	1.44	1	0.12	0.74
Final outcome	59.11	1	4.71	0.04
Residuals	439.07	35		

2.20.2) Levene's test

	Levene's test for homogeneity of variance	
	F test	p-value
HIV	F(1, 36)=1.02	0.32
Final outcome	F(1, 36)=8.59	<0.01

2.20.3) Final outcome LS means graph

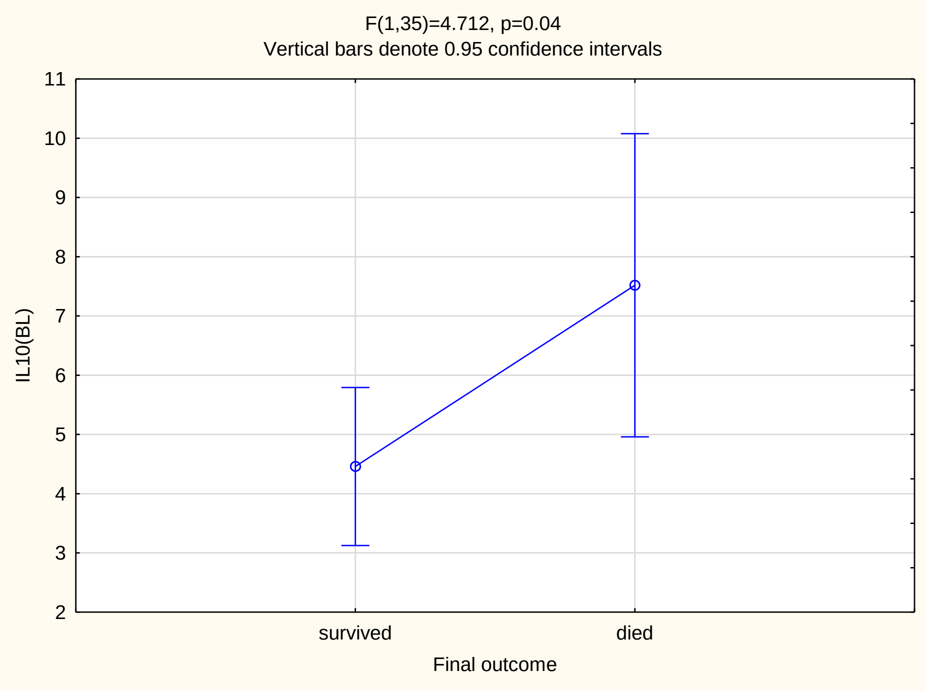

2.20.4) Welch tests

2.20.4.1) Final outcome adjusted means plot

2.21) TNFa(BL) with HIV

2.21.1) ANOVA

	ANOVA type: III			
	Sum Sq	DF	F value	p value
(Intercept)	1872.48	1	81.433	<0.01
HIV	40.37	1	1.756	0.19
Final outcome	0.09	1	0.004	0.95
Residuals	804.79	35		

2.21.2) Levene's test

	Levene's test for homogeneity of variance	
	F test	p-value
HIV	F(1, 36)=4.06	0.05
Final outcome	F(1, 36)=0.58	0.45

2.21.3) Final outcome LS means graph

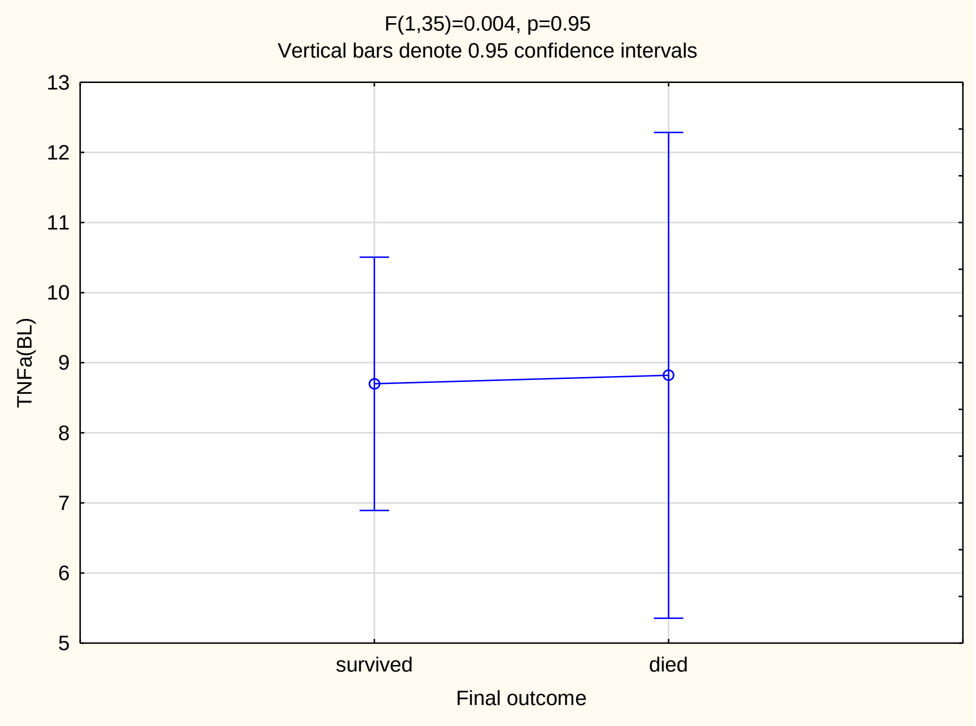

2.22) TNFa(AT)(winsorized) with HIV

2.22.1) ANOVA

	ANOVA type: III			
	Sum Sq	DF	F value	p value
(Intercept)	2.341	1	21.58	<0.01
HIV	0.003	1	0.03	0.87
Final outcome	0.203	1	1.87	0.18
Residuals	3.798	35		

2.22.2) Levene's test

	Levene's test for homogeneity of variance	
	F test	p-value
HIV	F(1, 36)=3.10	0.09
Final outcome	F(1, 36)=4.89	0.03

2.22.3) Final outcome LS means graph

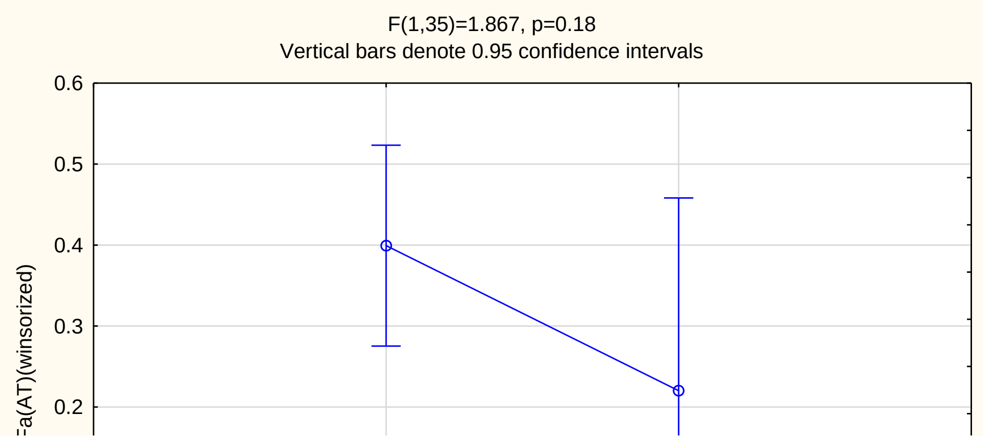

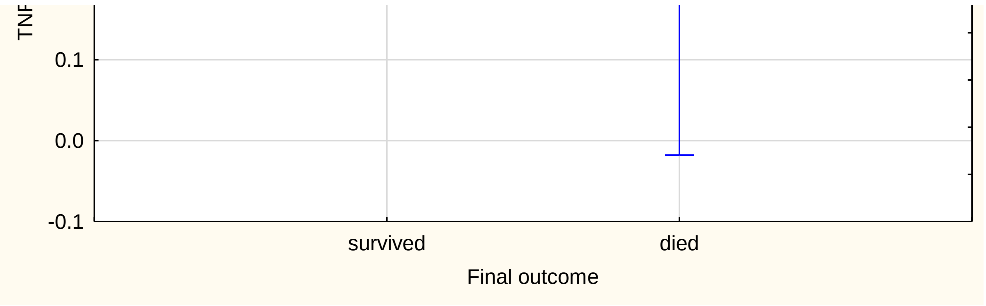

2.23) MCP1(AT)(winsorized) with HIV

2.23.1) ANOVA

	ANOVA type: III			
	Sum Sq	DF	F value	p value
(Intercept)	3493.99	1	23.06	<0.01
HIV	2.18	1	0.01	0.91
Final outcome	191.92	1	1.27	0.27
Residuals	5304.16	35		

2.23.2) Levene's test

	Levene's test for homogeneity of variance	
	F test	p-value
HIV	F(1, 36)=0.58	0.45
Final outcome	F(1, 36)=0.93	0.34

2.23.3) Final outcome LS means graph

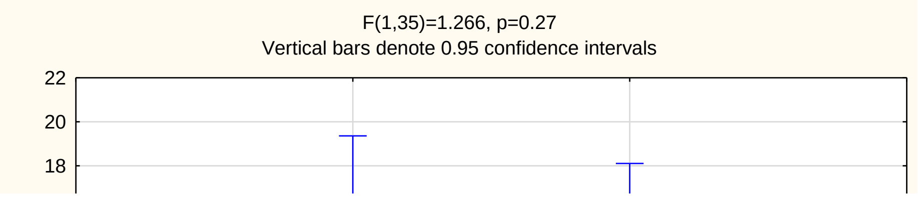

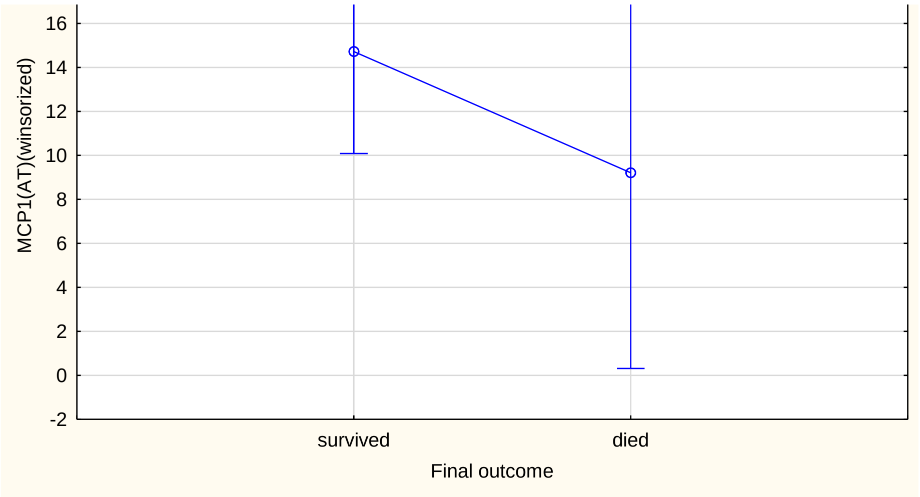

2.24) IL6(AT)(winsorized) with HIV

2.24.1) ANOVA

	ANOVA type: III			
	Sum Sq	DF	F value	p value
(Intercept)	32.13	1	21.25	<0.01
HIV	0.03	1	0.02	0.89
Final outcome	2	1	1.33	0.26
Residuals	52.92	35		

2.24.2) Levene's test

	Levene's test for homogeneity of variance	
	F test	p-value
HIV	F(1, 36)=0.38	0.54
Final outcome	F(1, 36)=2.13	0.15

2.24.3) Final outcome LS means graph

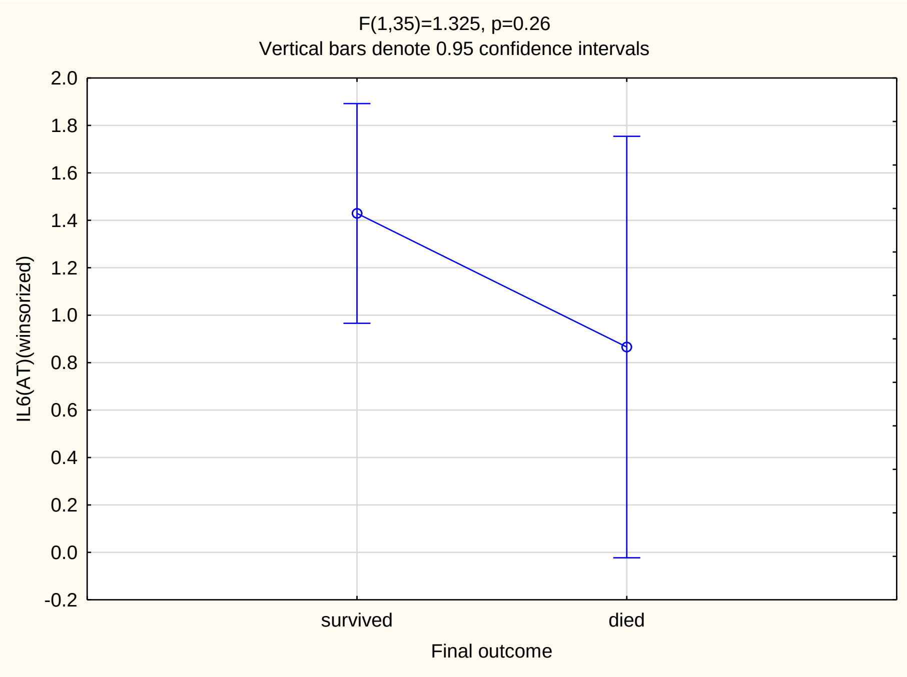

2.25) IL17 (AT)(winsorized) with HIV

2.25.1) ANOVA

	ANOVA type: III			
	Sum Sq	DF	F value	p value
(Intercept)	6.98	1	30.49	<0.01
HIV	0.04	1	0.17	0.69
Final outcome	0.36	1	1.58	0.22
Residuals	8.01	35		

2.25.2) Levene's test

	Levene's test for homogeneity of variance	
	F test	p-value
HIV	F(1, 36)=4.88	0.03
Final outcome	F(1, 36)=1.61	0.21

2.25.3) Final outcome LS means graph

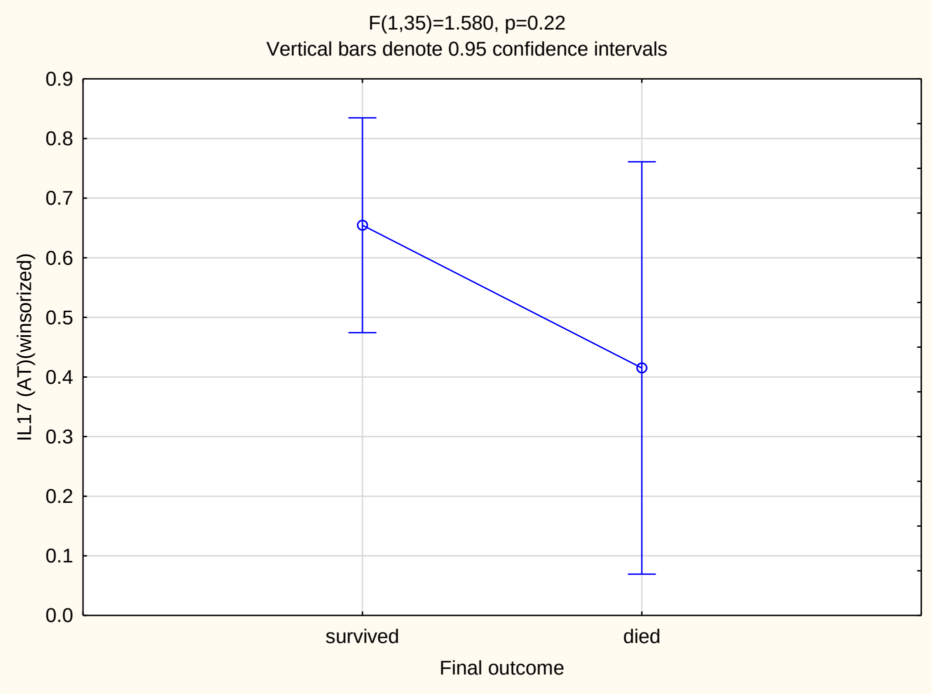

2.26) IL1b(BL)(winsorized) with HIV

2.26.1) ANOVA

	ANOVA type: III			
	Sum Sq	DF	F value	p value
(Intercept)	707.68	1	2189.96	<0.01
HIV	0.18	1	0.57	0.46
Final outcome	0.01	1	0.03	0.85
Residuals	11.31	35		

2.26.2) Levene's test

	Levene's test for homogeneity of variance	
	F test	p-value
HIV	F(1, 36)=0.33	0.57
Final outcome	F(1, 36)=2.31	0.14

2.26.3) Final outcome LS means graph

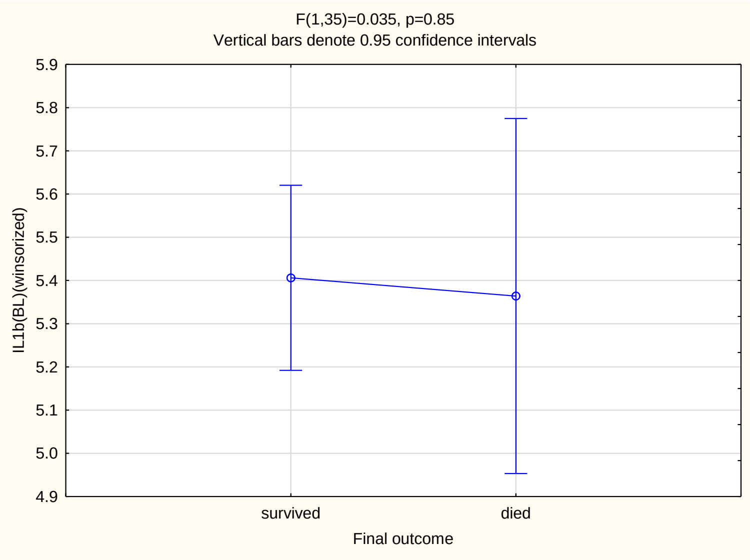

2.27) IL17(BL)(winsorized) with HIV

2.27.1) ANOVA

	ANOVA type: III			
	Sum Sq	DF	F value	p value
(Intercept)	192.73	1	25.45	<0.01
HIV	0.86	1	0.11	0.74
Final outcome	0.13	1	0.02	0.89
Residuals	265.01	35		

2.27.2) Levene's test

	Levene's test for homogeneity of variance	
	F test	p-value
HIV	F(1, 36)=0.16	0.69
Final outcome	F(1, 36)=0.07	0.8

2.27.3) Final outcome LS means graph

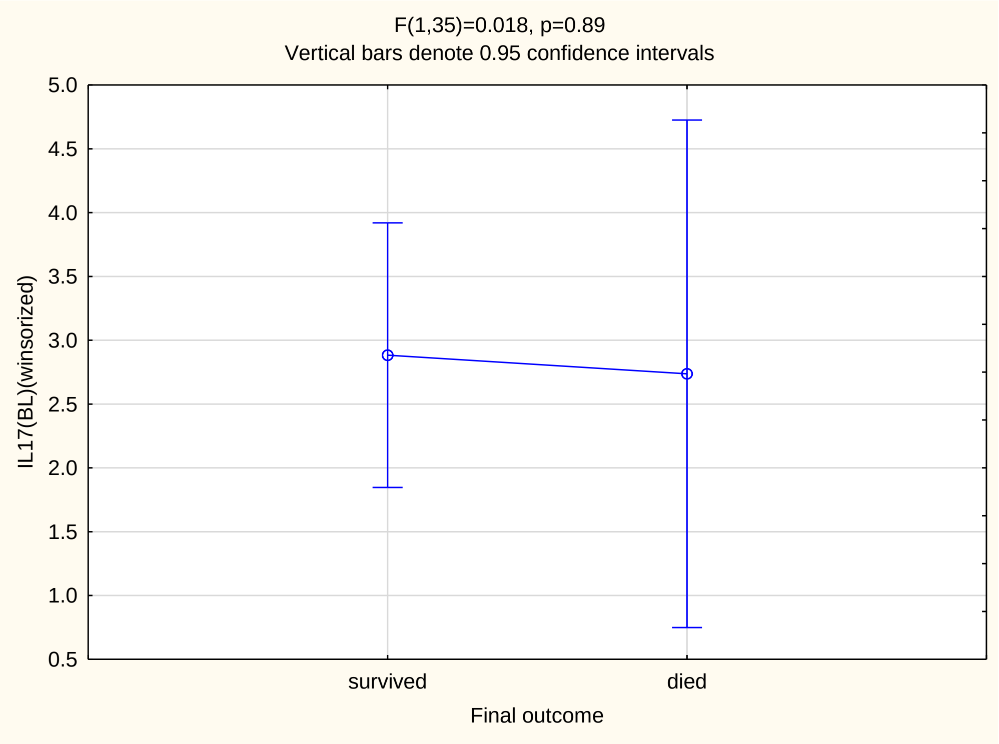

2.28) IL1b(FI AT)(winsorized) with HIV

2.28.1) ANOVA

	ANOVA type: III			
	Sum Sq	DF	F value	p value
(Intercept)	5.1	1	18.65	<0.01
HIV	0.02	1	0.08	0.77
Final outcome	0.87	1	3.18	0.08
Residuals	9.56	35		

2.28.2) Levene's test

	Levene's test for homogeneity of variance	
	F test	p-value
HIV	F(1, 36)=1.31	0.26
Final outcome	F(1, 36)=4.68	0.04

2.28.3) Final outcome LS means graph

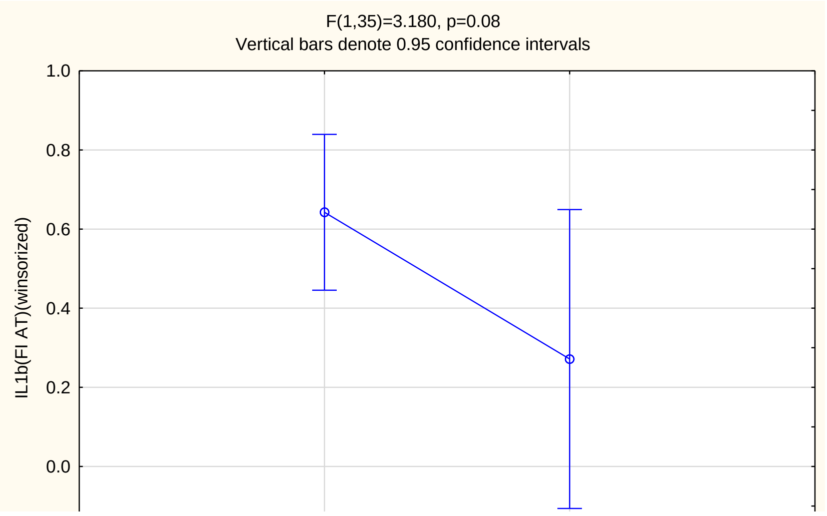

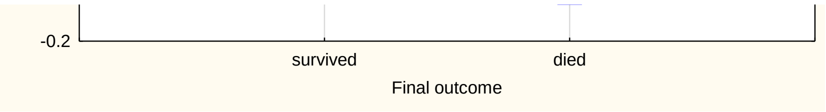

2.29) IL2 (AT) with severity

2.29.1) ANOVA

	ANOVA type: III			
	Sum Sq	DF	F value	p value
(Intercept)	126938.81	1	18.61	<0.01
severity	6282.08	1	0.92	0.34
Final outcome	6078.35	1	0.89	0.35
Residuals	238720.11	35		

2.29.2) Levene's test

	Levene's test for homogeneity of variance	
	F test	p-value
severity	F(1, 36)=3.38	0.07
Final outcome	F(1, 36)=3.05	0.09

2.29.3) Final outcome LS means graph

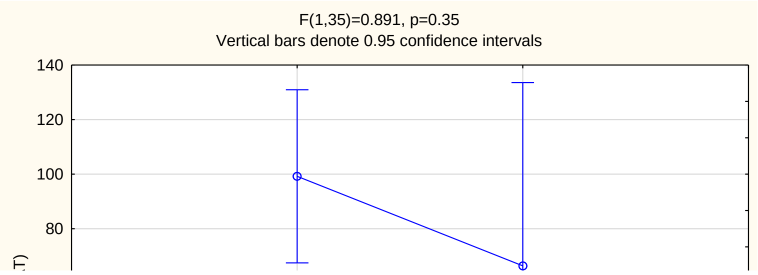

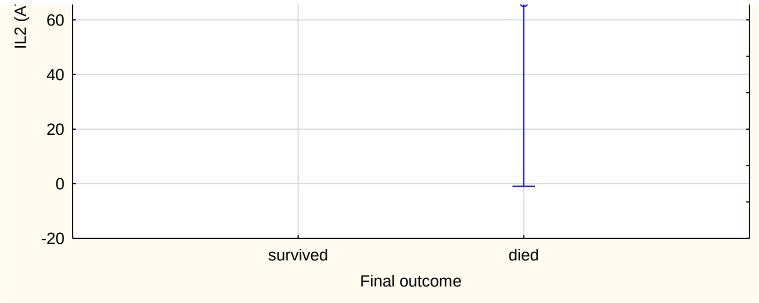

2.30) IL10(AT) with severity

2.30.1) ANOVA

	ANOVA type: III			
	Sum Sq	DF	F value	p value
(Intercept)	17.13	1	24.54	<0.01
severity	0.89	1	1.27	0.27
Final outcome	0.34	1	0.49	0.49
Residuals	24.43	35		

2.30.2) Levene's test

	Levene's test for homogeneity of variance	
	F test	p-value
severity	F(1, 36)=4.84	0.03
Final outcome	F(1, 36)=2.08	0.16

2.30.3) Final outcome LS means graph

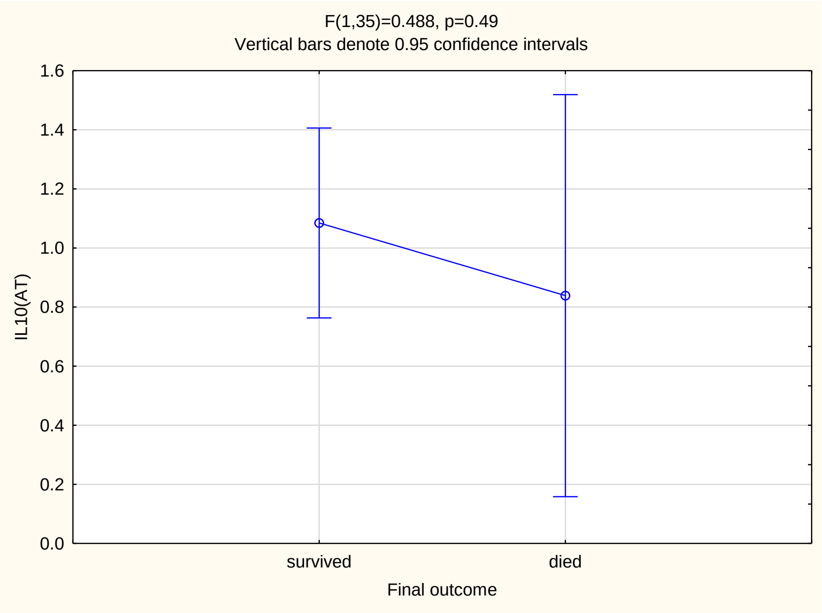

2.31) MCP1(BL) with severity

2.31.1) ANOVA

	ANOVA type: III			
	Sum Sq	DF	F value	p value
(Intercept)	3898228.64	1	23.59	<0.01
severity	114633.74	1	0.69	0.41
Final outcome	6557.74	1	0.04	0.84
Residuals	5784364.06	35		

2.31.2) Levene's test

	Levene's test for homogeneity of variance	
	F test	p-value
severity	F(1, 36)=0.03	0.87
Final outcome	F(1, 36)=0.44	0.51

2.31.3) Final outcome LS means graph

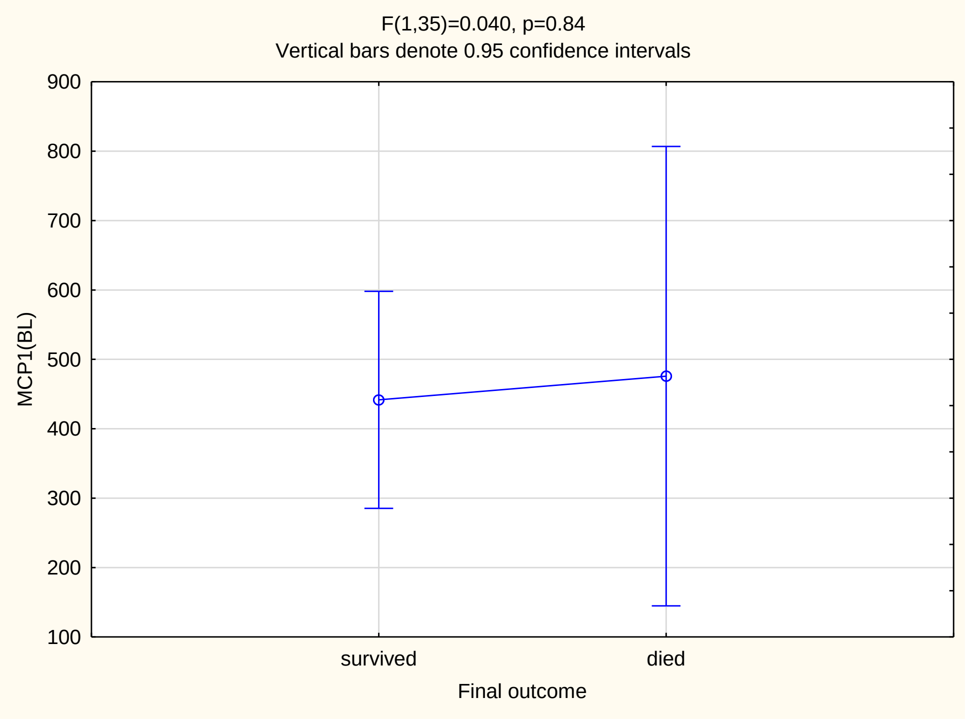

2.32) IL6(BL) with severity

2.32.1) ANOVA

	ANOVA type: III			
	Sum Sq	DF	F value	p value

(Intercept)	2452.82	1	18.55	<0.01
severity	506.76	1	3.83	0.06
Final outcome	25.74	1	0.19	0.66
Residuals	4627.5	35		

2.32.2) Levene's test

	Levene's test for homogeneity of variance	
	F test	p-value
severity	F(1, 36)=5.15	0.03
Final outcome	F(1, 36)=0.60	0.44

2.32.3) Final outcome LS means graph

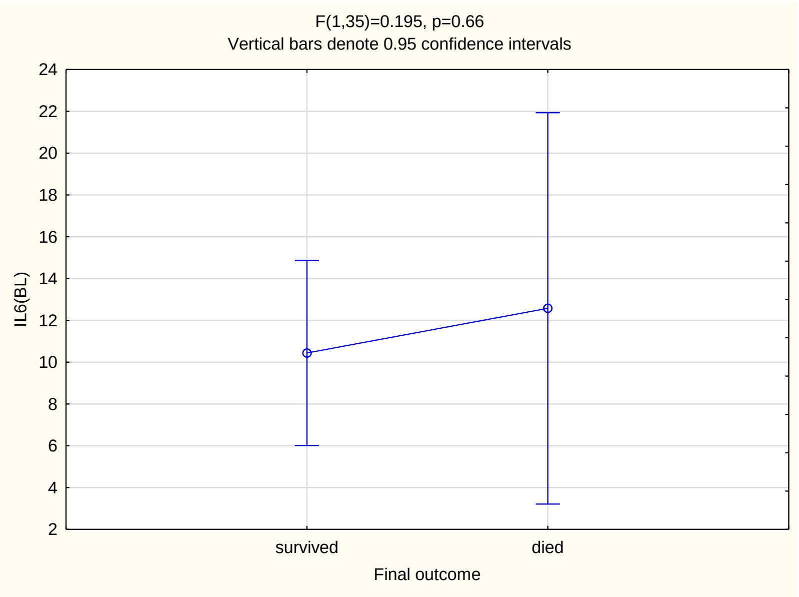

2.33) IL2(BL) with severity

2.33.1) ANOVA

	ANOVA type: III			
	Sum Sq	DF	F value	p value
(Intercept)	409003.52	1	208.06	<0.01
severity	925.2	1	0.47	0.5
Final outcome	70.87	1	0.04	0.85
Residuals	68804.18	35		

2.33.2) Levene's test

	Levene's test for homogeneity of variance	
	F test	p-value
severity	F(1, 36)=0.00	0.96
Final outcome	F(1, 36)=0.79	0.38

2.33.3) Final outcome LS means graph

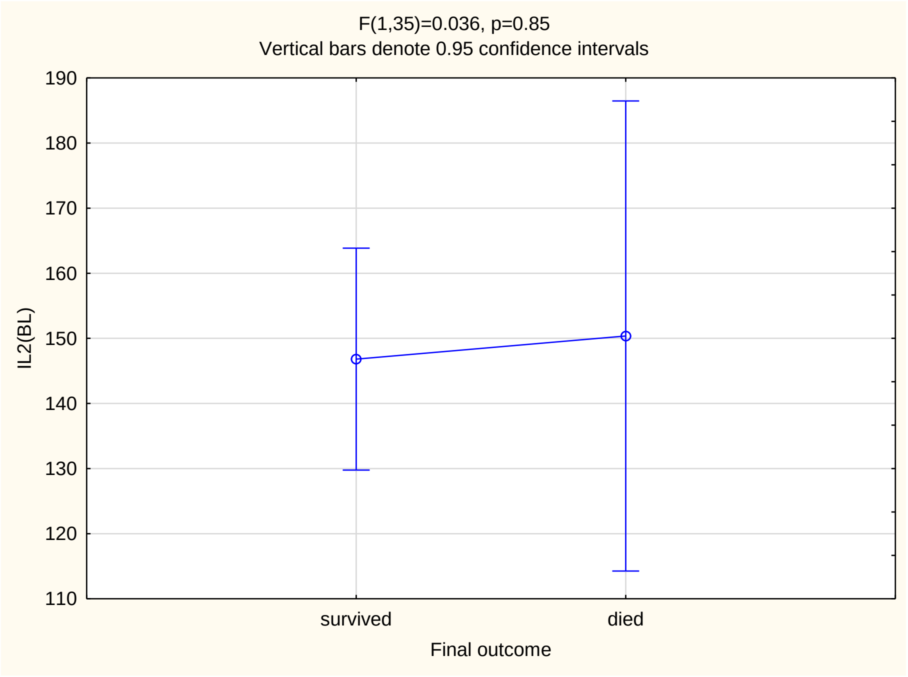

2.34) IL10(BL) with severity

2.34.1) ANOVA

	ANOVA type: III			
	Sum Sq	DF	F value	p value
(Intercept)	600.64	1	48.2	<0.01
severity	4.33	1	0.35	0.56
Final outcome	42.91	1	3.44	0.07
Residuals	436.18	35		

2.34.2) Levene's test

	Levene's test for homogeneity of variance	
	F test	p-value
severity	F(1, 36)=3.84	0.06
Final outcome	F(1, 36)=8.59	<0.01

2.34.3) Final outcome LS means graph

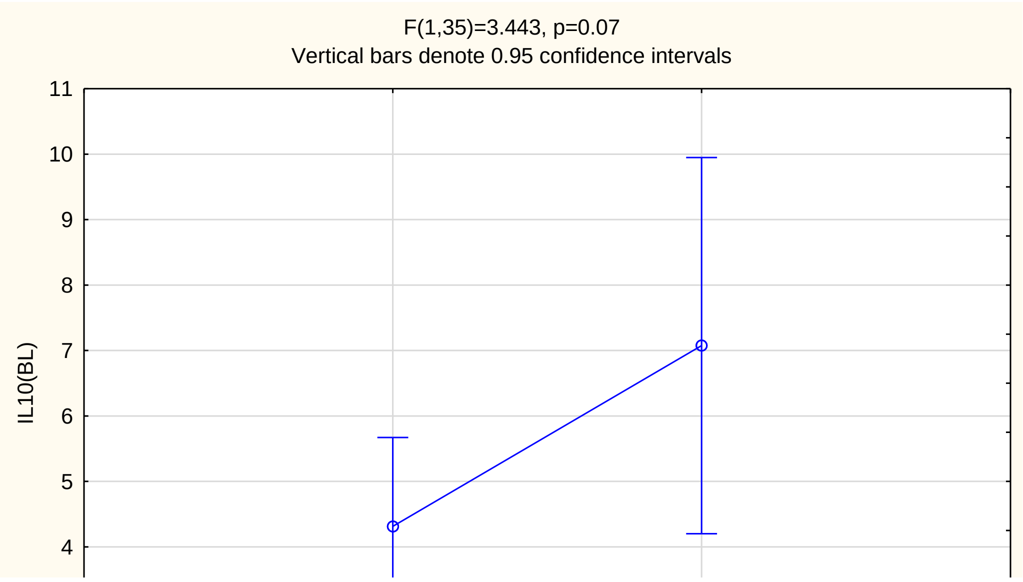

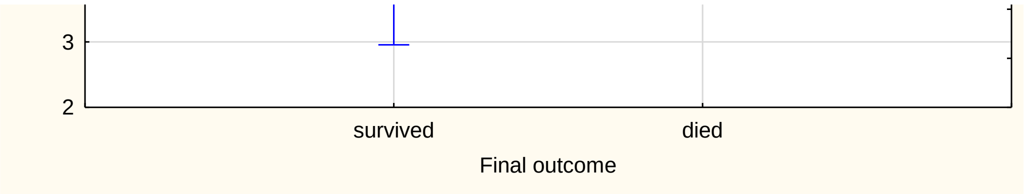

2.34.4) Welch tests

2.34.4.1) Final outcome adjusted means plot

2.35) TNFa(BL) with severity

2.35.1) ANOVA

	ANOVA type: III			
	Sum Sq	DF	F value	p value
(Intercept)	1525.92	1	63.22	<0.01
severity	0.43	1	0.02	0.89
Final outcome	0.39	1	0.02	0.9
Residuals	844.73	35		

2.35.2) Levene's test

	Levene's test for homogeneity of variance	
	F test	p-value
severity	F(1, 36)=0.02	0.89
Final outcome	F(1, 36)=0.58	0.45

2.35.3) Final outcome LS means graph

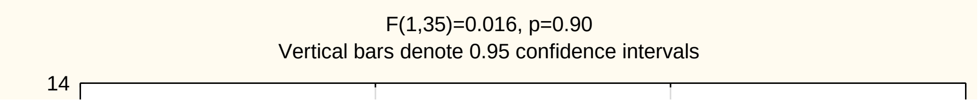

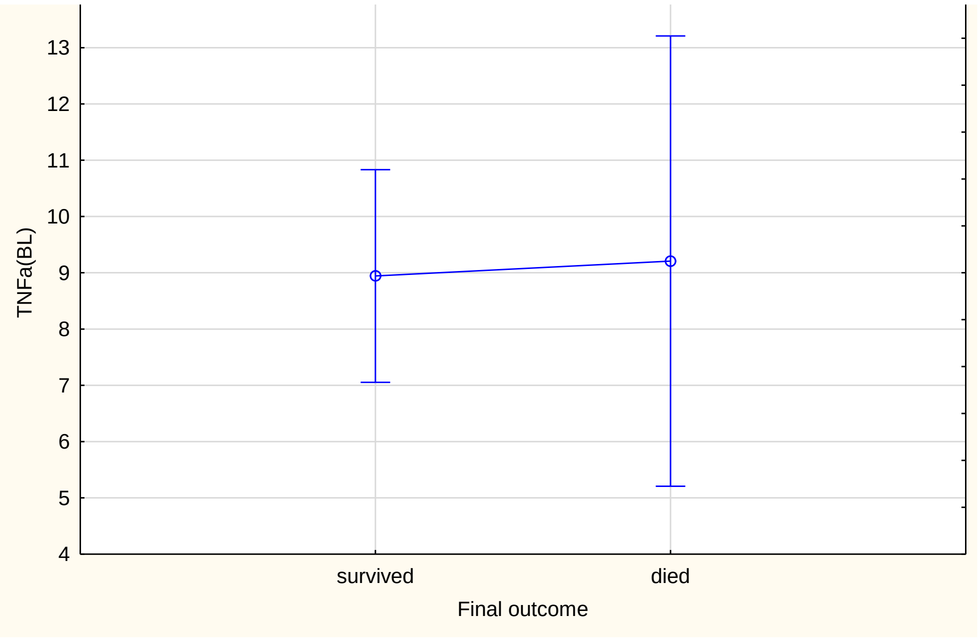

2.36) TNFa(AT)(winsorized) with severity

2.36.1) ANOVA

	ANOVA type: III			
	Sum Sq	DF	F value	p value
(Intercept)	2.05	1	19.05	<0.01
severity	0.03	1	0.29	0.6
Final outcome	0.13	1	1.24	0.27
Residuals	3.77	35		

2.36.2) Levene's test

	Levene's test for homogeneity of variance	
	F test	p-value
severity	F(1, 36)=1.09	0.3
Final outcome	F(1, 36)=4.89	0.03

2.36.3) Final outcome LS means graph

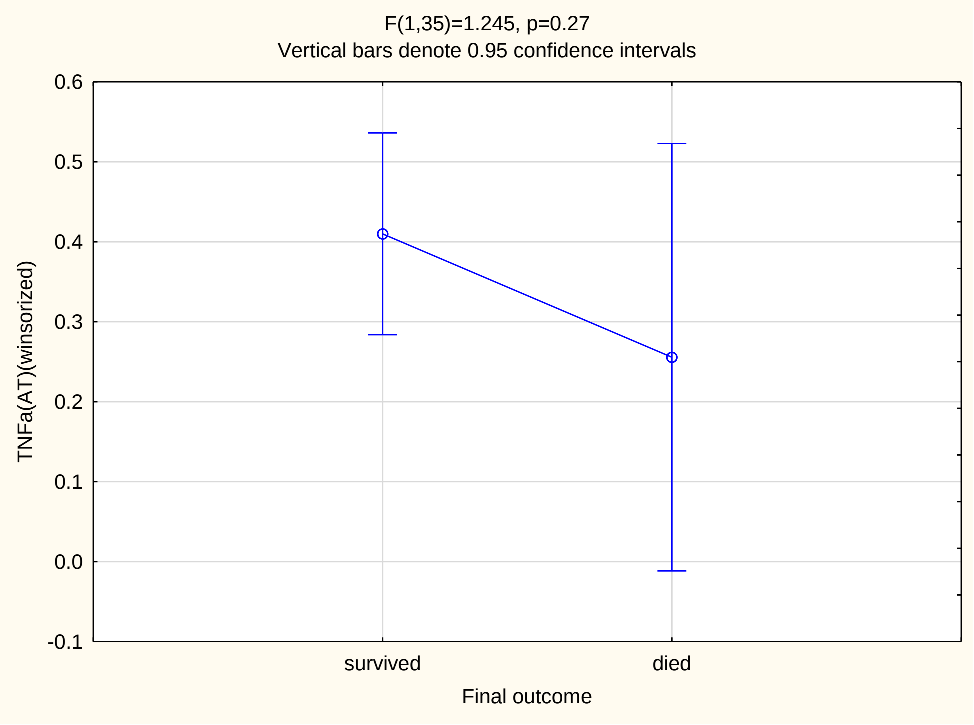

2.37) MCP1(AT)(winsorized) with severity

2.37.1) ANOVA

	ANOVA type: III			
	Sum Sq	DF	F value	p value
(Intercept)	2816.04	1	18.62	<0.01
severity	12.16	1	0.08	0.78
Final outcome	143.09	1	0.95	0.34
Residuals	5294.17	35		

2.37.2) Levene's test

	Levene's test for homogeneity of variance	
	F test	p-value
severity	F(1, 36)=0.48	0.49
Final outcome	F(1, 36)=0.93	0.34

2.37.3) Final outcome LS means graph

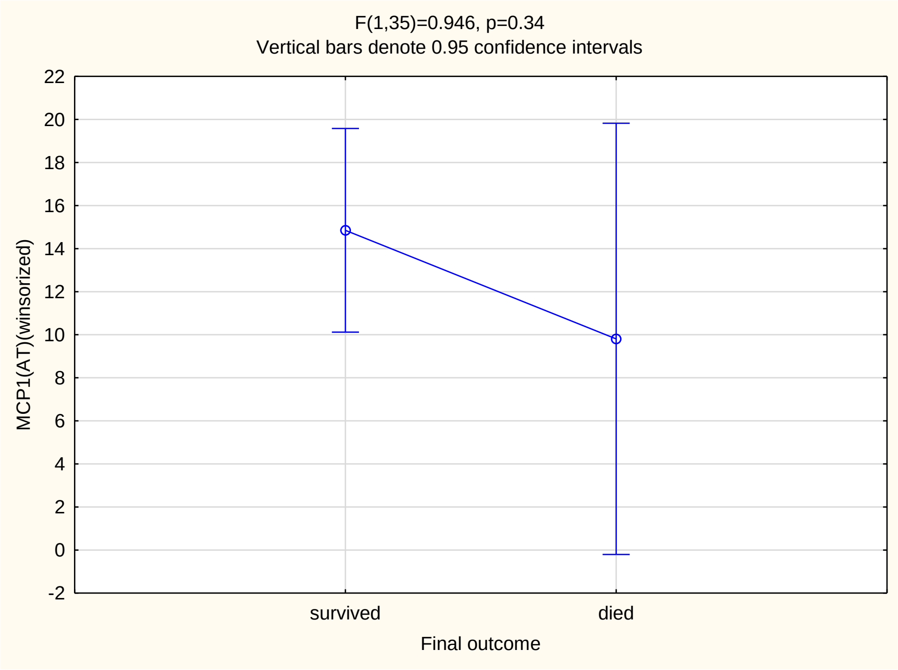

2.38) IL6(AT)(winsorized) with severity

2.38.1) ANOVA

	ANOVA type: III			
	Sum Sq	DF	F value	p value
(Intercept)	28.09	1	18.77	<0.01
severity	0.57	1	0.38	0.54
Final outcome	1.19	1	0.8	0.38
Residuals	52.38	35		

2.38.2) Levene's test

	Levene's test for homogeneity of variance	
	F test	p-value
severity	F(1, 36)=1.87	0.18
Final outcome	F(1, 36)=2.13	0.15

2.38.3) Final outcome LS means graph

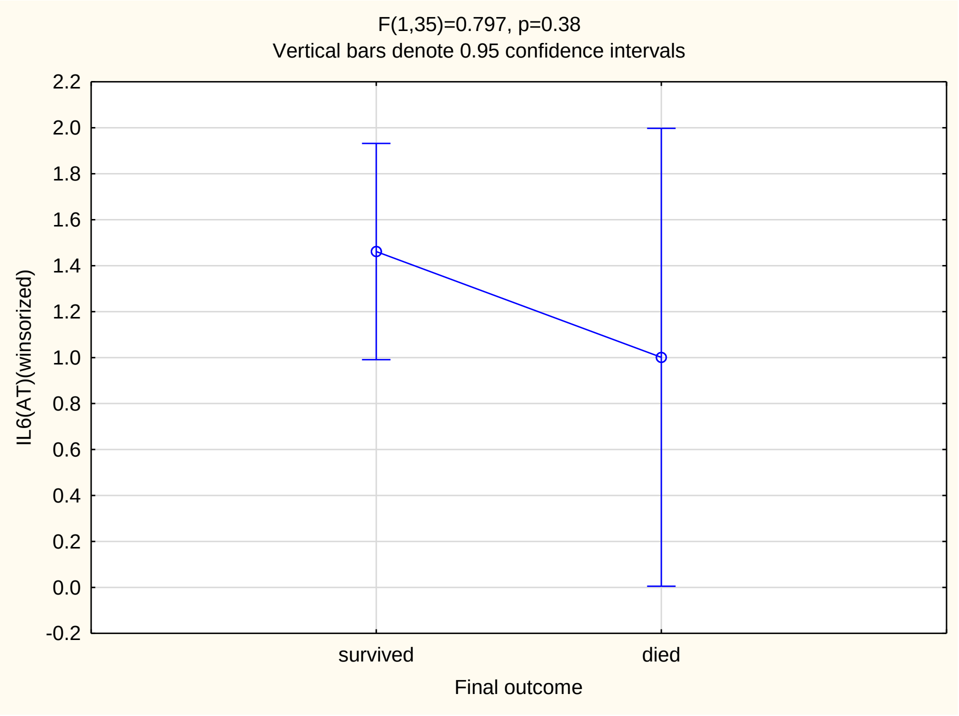

2.39) IL17 (AT)(winsorized) with severity

2.39.1) ANOVA

	ANOVA type: III			
	Sum Sq	DF	F value	p value
(Intercept)	6.21	1	27.31	<0.01
severity	0.09	1	0.42	0.52
Final outcome	0.21	1	0.94	0.34
Residuals	7.96	35		

2.39.2) Levene's test

	Levene's test for homogeneity of variance	
	F test	p-value
severity	F(1, 36)=2.44	0.13
Final outcome	F(1, 36)=1.61	0.21

2.39.3) Final outcome LS means graph

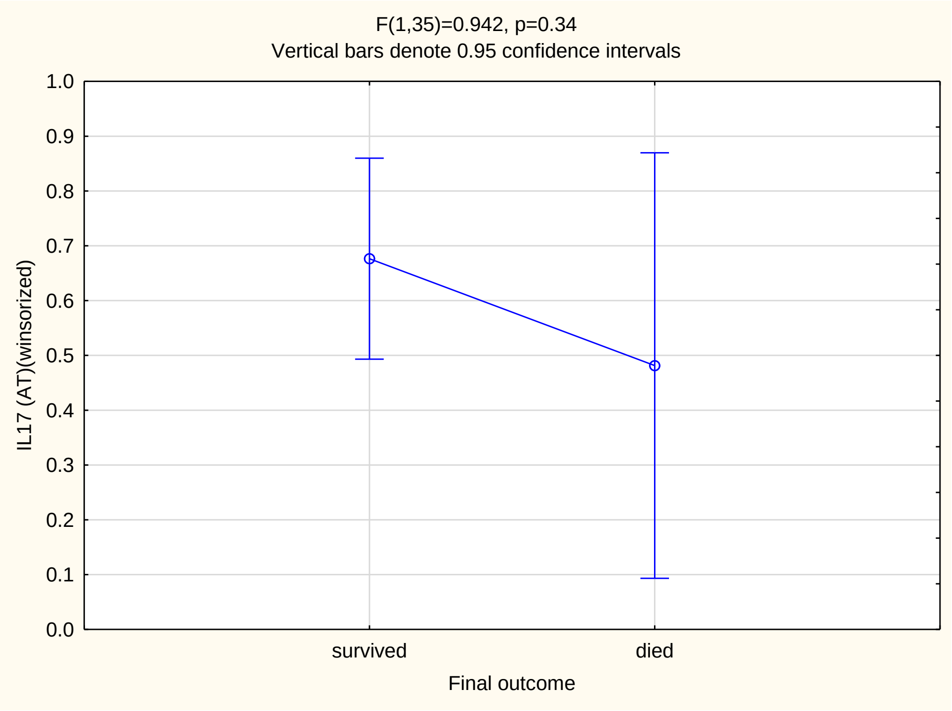

2.40) IL1b(BL)(winsorized) with severity

2.40.1) ANOVA

	ANOVA type: III			
	Sum Sq	DF	F value	p value
(Intercept)	547.15	1	1710.48	<0.01
severity	0.297	1	0.93	0.34
Final outcome	0.005	1	0.02	0.9
Residuals	11.196	35		

2.40.2) Levene's test

	Levene's test for homogeneity of variance	
	F test	p-value
severity	F(1, 36)=0.54	0.47
Final outcome	F(1, 36)=2.31	0.14

2.40.3) Final outcome LS means graph

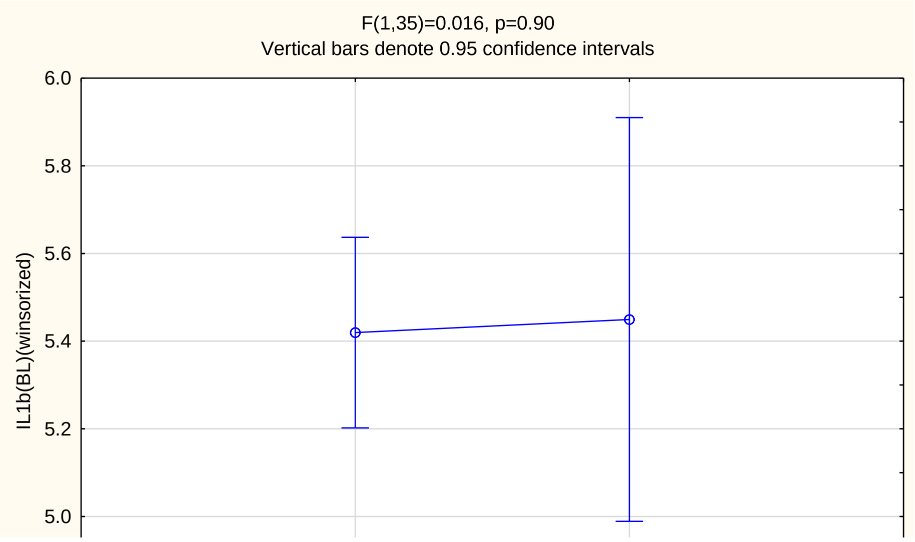

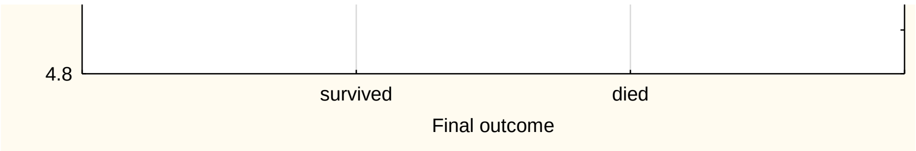

2.41) IL17(BL)(winsorized) with severity

2.41.1) ANOVA

	ANOVA type: III			
	Sum Sq	DF	F value	p value
(Intercept)	139.26	1	18.34	<0.01
severity	0.08	1	0.01	0.92
Final outcome	0.21	1	0.03	0.87
Residuals	265.79	35		

2.41.2) Levene's test

	Levene's test for homogeneity of variance	
	F test	p-value
severity	F(1, 36)=0.01	0.93
Final outcome	F(1, 36)=0.07	0.8

2.41.3) Final outcome LS means graph

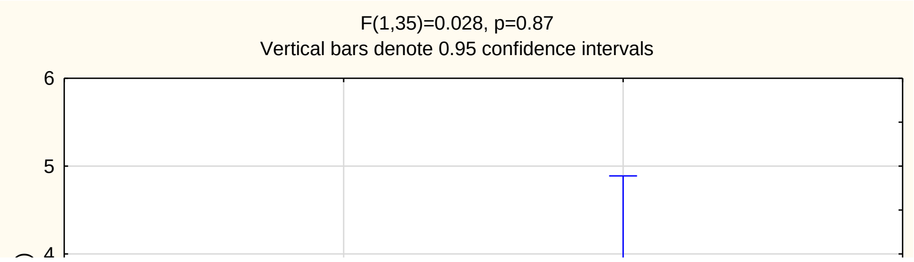

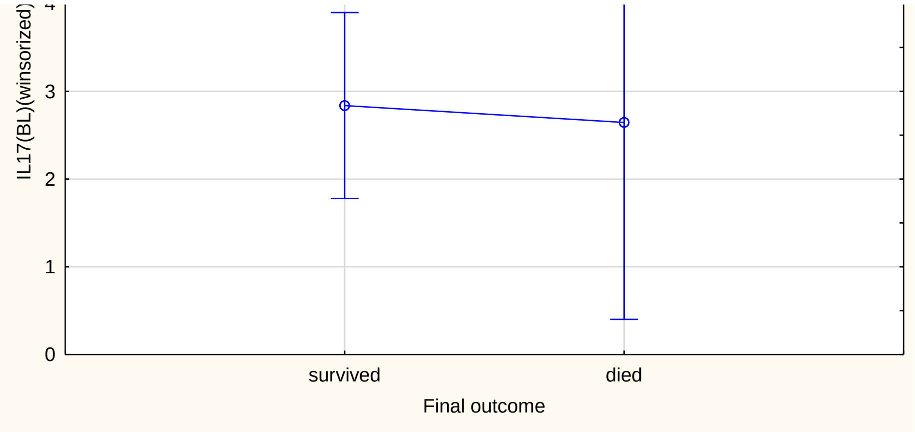

2.42) IL1b(FI AT)(winsorized) with severity

2.42.1) ANOVA

	ANOVA type: III			
	Sum Sq	DF	F value	p value
(Intercept)	5.02	1	18.8	<0.01
severity	0.23	1	0.88	0.36
Final outcome	0.52	1	1.93	0.17
Residuals	9.35	35		

2.42.2) Levene's test

	Levene's test for homogeneity of variance	
	F test	p-value
severity	F(1, 36)=3.47	0.07
Final outcome	F(1, 36)=4.68	0.04

2.42.3) Final outcome LS means graph

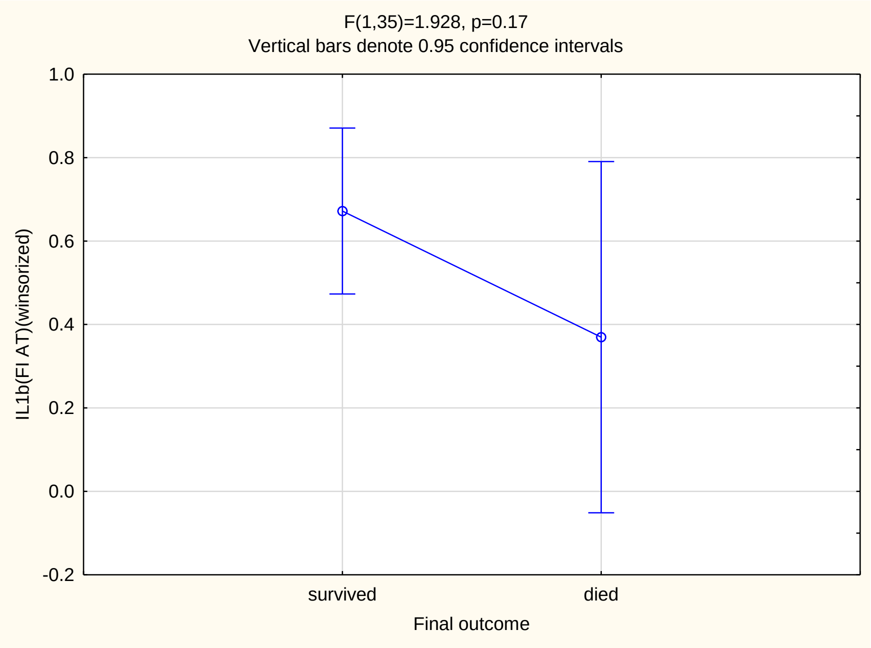

2.43) IL2 (AT) with Sex

2.43.1) ANOVA

	ANOVA type: III			
	Sum Sq	DF	F value	p value
(Intercept)	83192.73	1	12.04	<0.01
Sex	3244.64	1	0.47	0.5
Final outcome	13680.94	1	1.98	0.17
Residuals	241757.55	35		

2.43.2) Levene's test

	Levene's test for homogeneity of variance	
	F test	p-value
Sex	F(1, 36)=0.00	1
Final outcome	F(1, 36)=3.05	0.09

2.43.3) Final outcome LS means graph

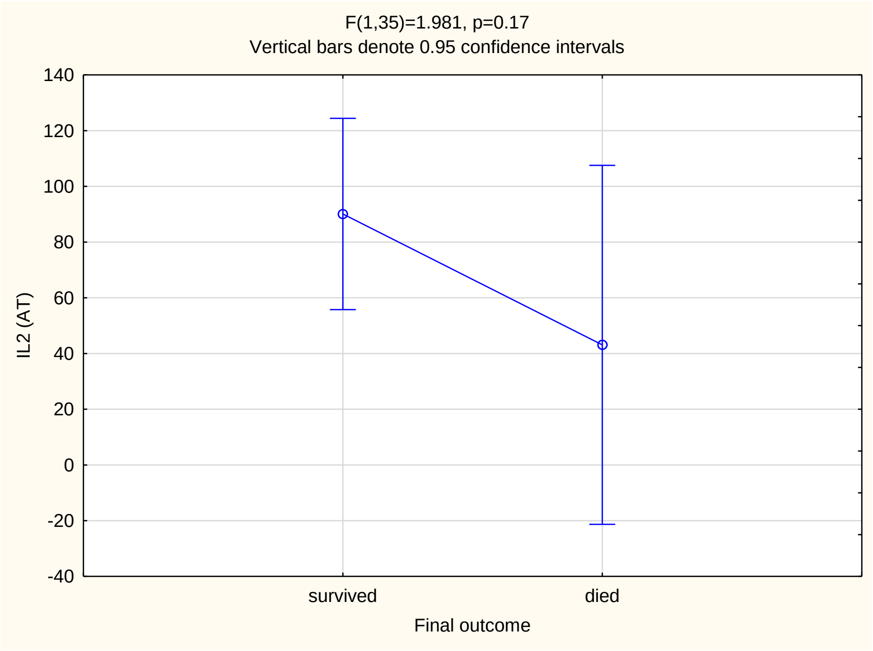

2.44) IL10(AT) with Sex

2.44.1) ANOVA

	ANOVA type: III			
	Sum Sq	DF	F value	p value

(Intercept)	11.74	1	16.38	<0.01
Sex	0.24	1	0.34	0.56
Final outcome	1.01	1	1.41	0.24
Residuals	25.07	35		

2.44.2) Levene's test

	Levene's test for homogeneity of variance	
	F test	p-value
Sex	F(1, 36)=0.09	0.76
Final outcome	F(1, 36)=2.08	0.16

2.44.3) Final outcome LS means graph

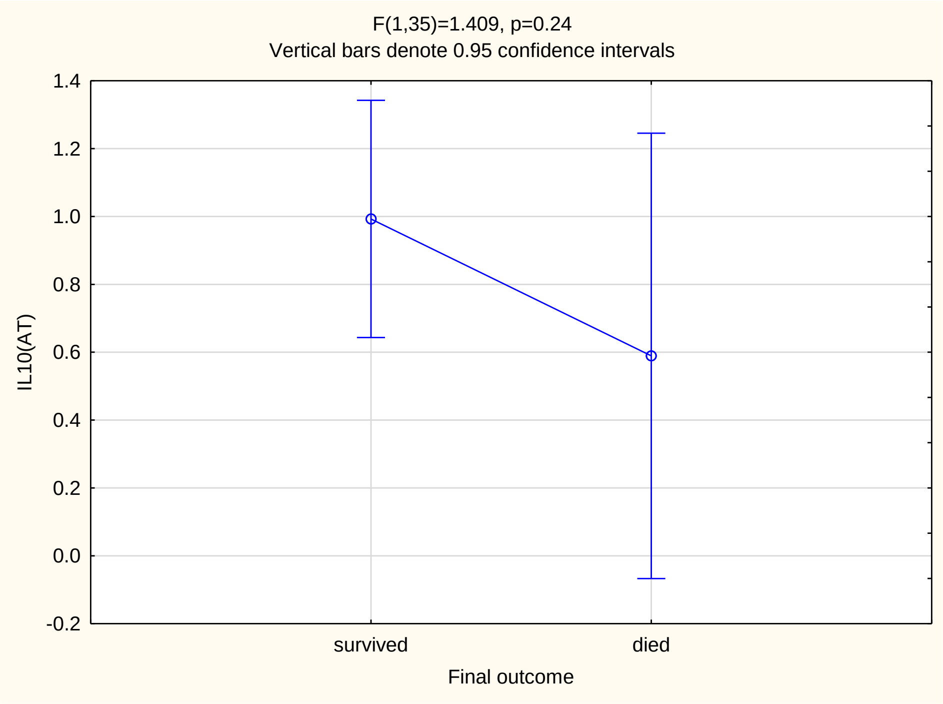

2.45) MCP1(BL) with Sex

2.45.1) ANOVA

	ANOVA type: III			
	Sum Sq	DF	F value	p value
(Intercept)	4448800.62	1	26.45	<0.01
Sex	11115.68	1	0.07	0.8
Final outcome	35254.83	1	0.21	0.65
Residuals	5887882.12	35		

2.45.2) Levene's test

	Levene's test for homogeneity of variance	
	F test	p-value
Sex	F(1, 36)=0.85	0.36
Final outcome	F(1, 36)=0.44	0.51

2.45.3) Final outcome LS means graph

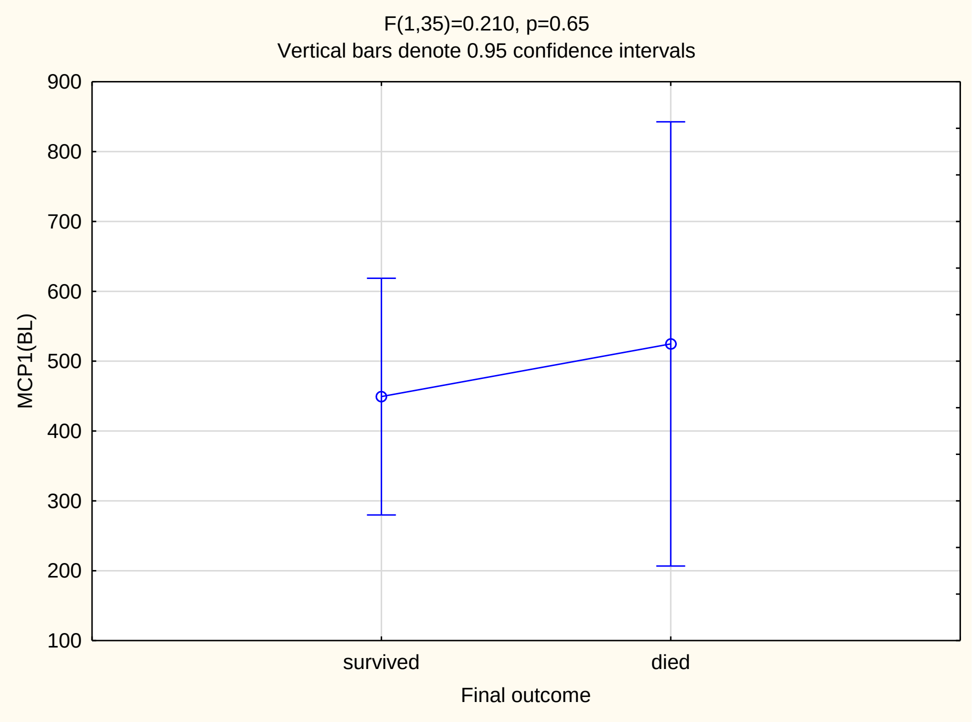

2.46) IL6(BL) with Sex

2.46.1) ANOVA

	ANOVA type: III			
	Sum Sq	DF	F value	p value
(Intercept)	3501.42	1	23.97	<0.01
Sex	21.66	1	0.15	0.7
Final outcome	155.71	1	1.07	0.31
Residuals	5112.6	35		

2.46.2) Levene's test

	Levene's test for homogeneity of variance	
	F test	p-value
Sex	F(1, 36)=0.01	0.9
Final outcome	F(1, 36)=0.60	0.44

2.46.3) Final outcome LS means graph

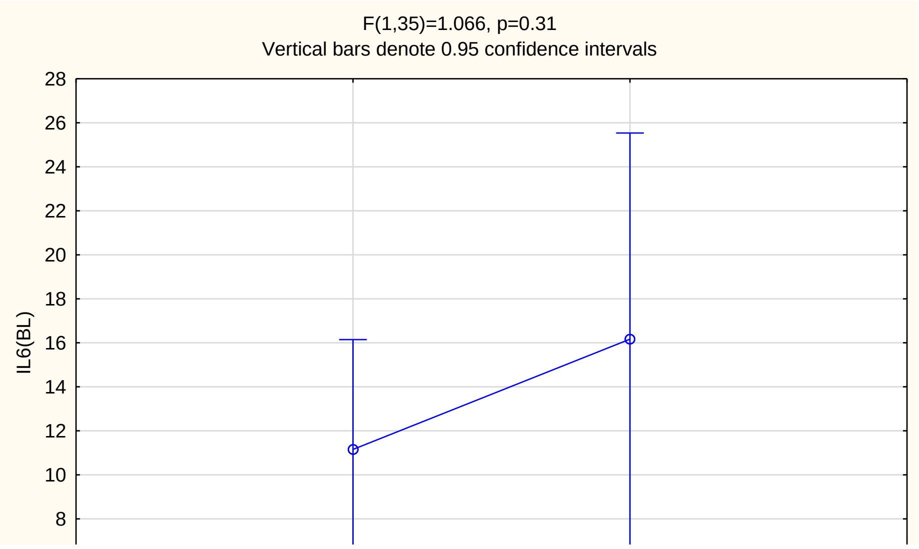

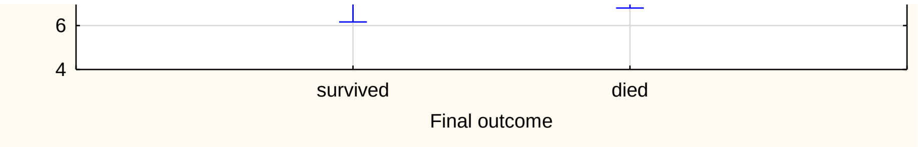

2.47) IL2(BL) with Sex

2.47.1) ANOVA

	ANOVA type: III			
	Sum Sq	DF	F value	p value
(Intercept)	392922.49	1	197.233	<0.01
Sex	3.17	1	0.002	0.97
Final outcome	3.72	1	0.002	0.97
Residuals	69726.21	35		

2.47.2) Levene's test

	Levene's test for homogeneity of variance	
	F test	p-value
Sex	F(1, 36)=0.22	0.64
Final outcome	F(1, 36)=0.79	0.38

2.47.3) Final outcome LS means graph

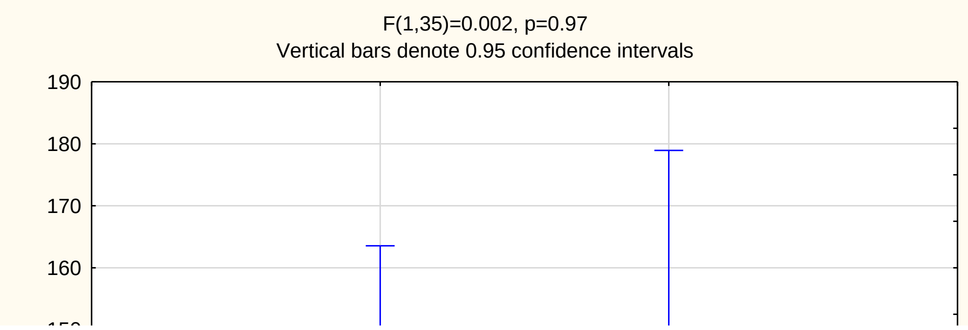

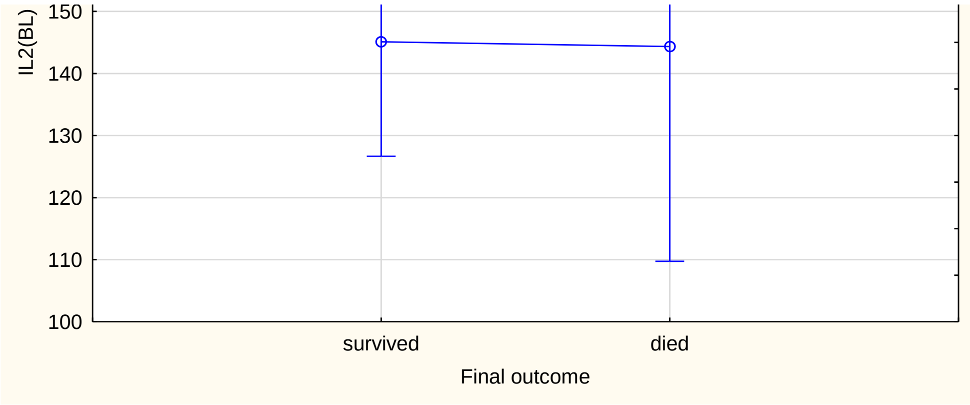

2.48) IL10(BL) with Sex

2.48.1) ANOVA

	ANOVA type: III			
	Sum Sq	DF	F value	p value
(Intercept)	499.85	1	44.18	<0.01
Sex	44.54	1	3.94	0.06
Final outcome	44.72	1	3.95	0.05
Residuals	395.97	35		

2.48.2) Levene's test

	Levene's test for homogeneity of variance	
	F test	p-value
Sex	F(1, 36)=5.18	0.03
Final outcome	F(1, 36)=8.59	<0.01

2.48.3) Final outcome LS means graph

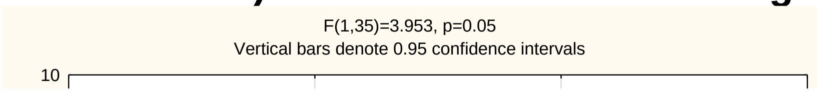

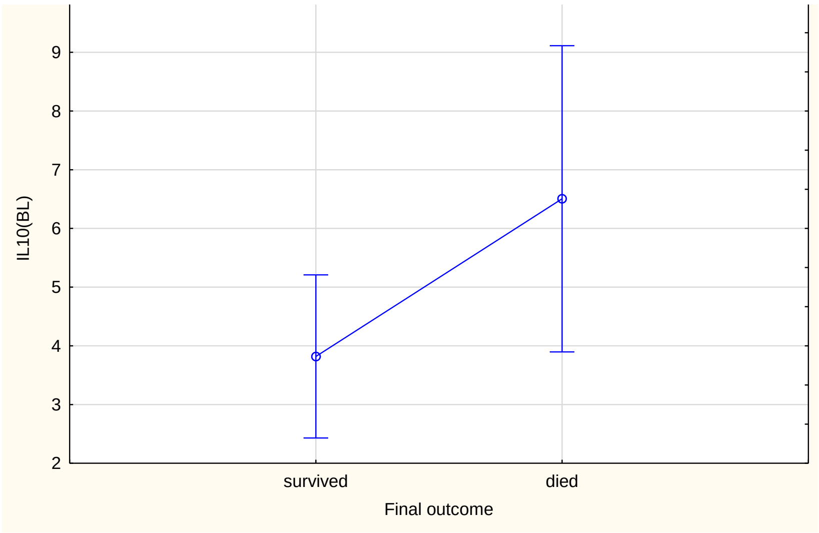

2.48.4) Welch tests

2.48.4.1) Final outcome adjusted means plot

2.49) TNFa(BL) with Sex

2.49.1) ANOVA

	ANOVA type: III			
	Sum Sq	DF	F value	p value
(Intercept)	1179.45	1	54.17	<0.01
Sex	83.06	1	3.81	0.06
Final outcome	0.65	1	0.03	0.86
Residuals	762.1	35		

2.49.2) Levene's test

	Levene's test for homogeneity of variance	
	F test	p-value
Sex	F(1, 36)=3.44	0.07
Final outcome	F(1, 36)=0.58	0.45

2.49.3) Final outcome LS means graph

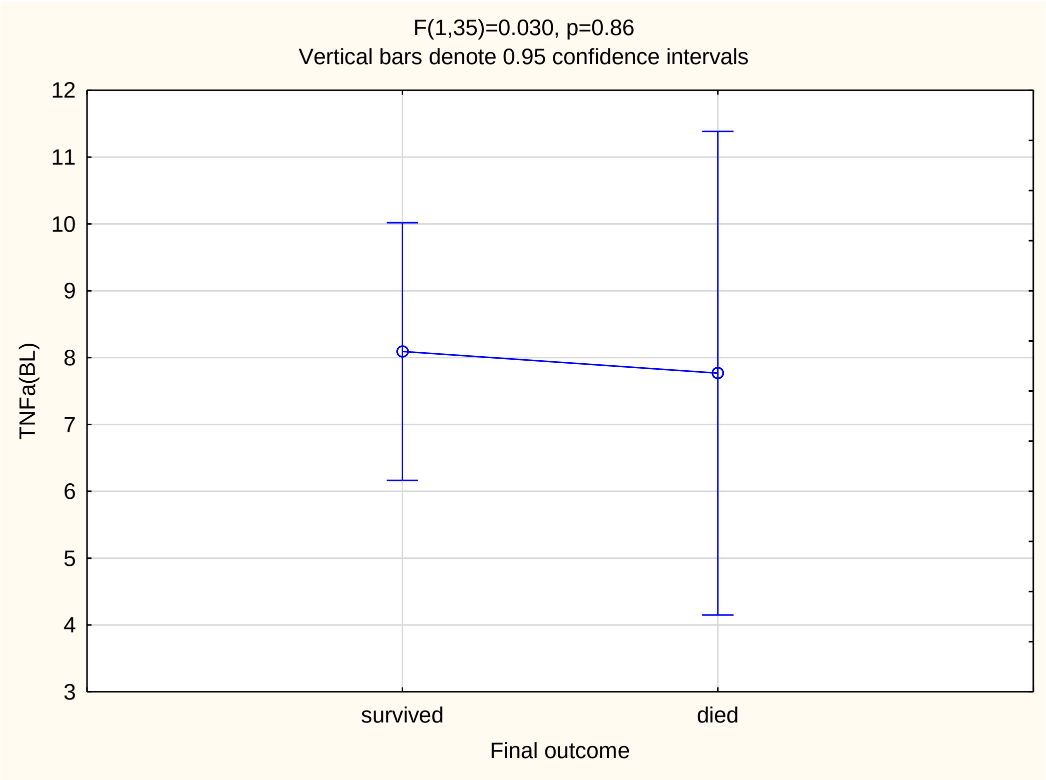

2.50) TNFa(AT)(winsorized) with Sex

2.50.1) ANOVA

	ANOVA type: III

	Sum Sq	DF	F value	p value
(Intercept)	1.56	1	14.52	<0.01
Sex	0.04	1	0.37	0.55
Final outcome	0.22	1	2.07	0.16
Residuals	3.76	35		

2.50.2) Levene's test

	Levene's test for homogeneity of variance	
	F test	p-value
Sex	F(1, 36)=0.33	0.57
Final outcome	F(1, 36)=4.89	0.03

2.50.3) Final outcome LS means graph

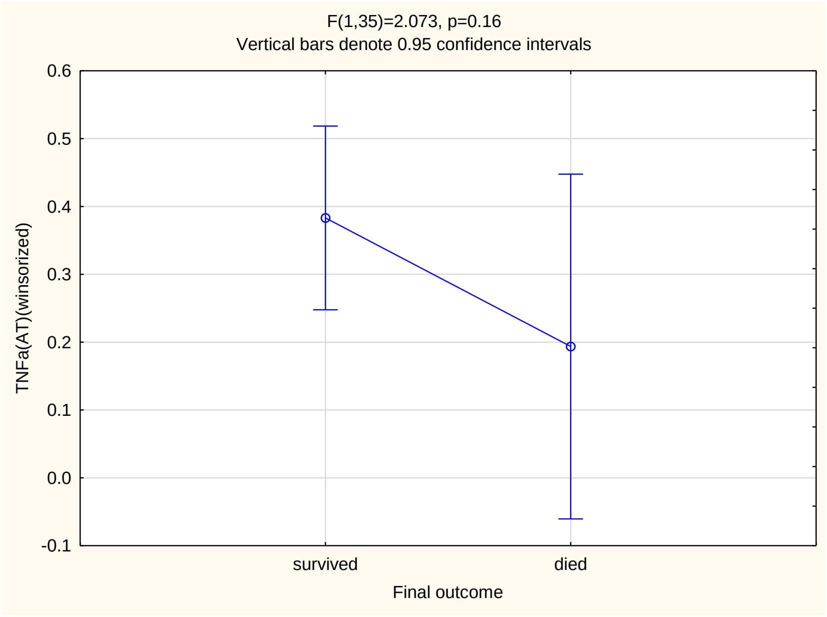

2.51) MCP1(AT)(winsorized) with Sex

2.51.1) ANOVA

	ANOVA type: III			
	Sum Sq	DF	F value	p value
(Intercept)	2686.2	1	17.719	<0.01
Sex	0.23	1	0.001	0.97
Final outcome	187.53	1	1.237	0.27
Residuals	5306.11	35		

2.51.2) Levene's test

	Levene's test for homogeneity of variance	
	F test	p-value
Sex	F(1, 36)=0.47	0.5
Final outcome	F(1, 36)=0.93	0.34

2.51.3) Final outcome LS means graph

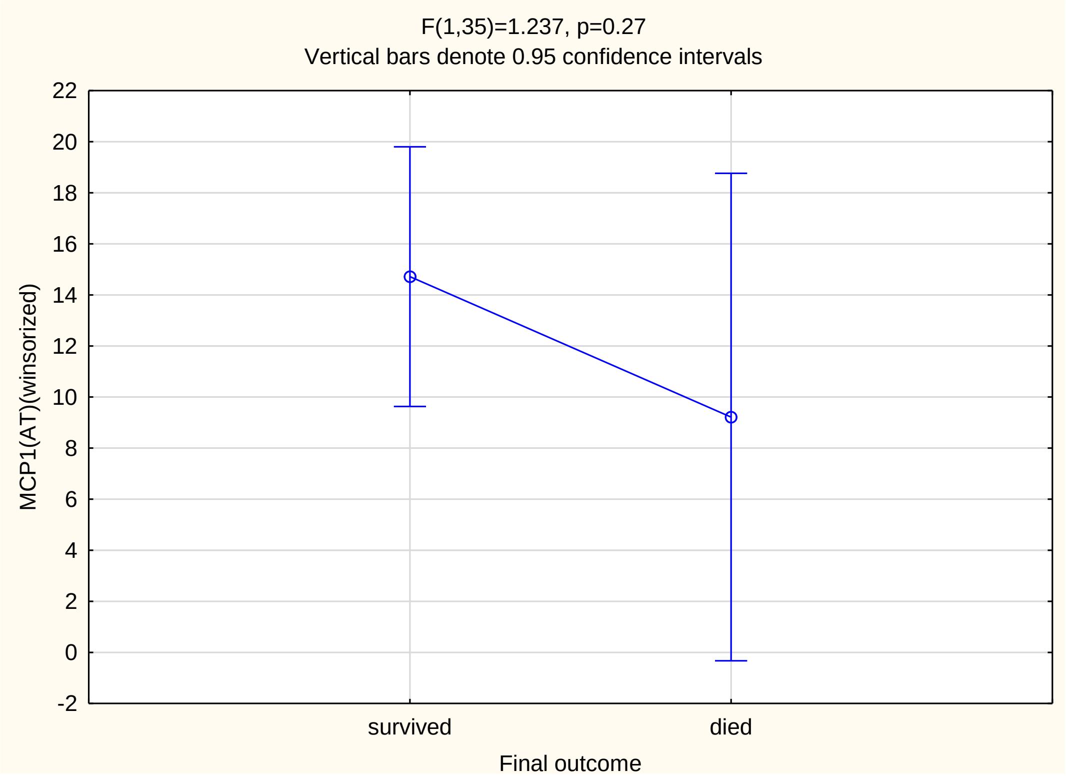

2.52) IL6(AT)(winsorized) with Sex

2.52.1) ANOVA

	ANOVA type: III			
	Sum Sq	DF	F value	p value
(Intercept)	23.03	1	15.25	<0.01
Sex	0.08	1	0.05	0.82
Final outcome	2.09	1	1.38	0.25
Residuals	52.87	35		

2.52.2) Levene's test

	Levene's test for homogeneity of variance	
	F test	p-value
Sex	F(1, 36)=0.74	0.4
Final outcome	F(1, 36)=2.13	0.15

2.52.3) Final outcome LS means graph

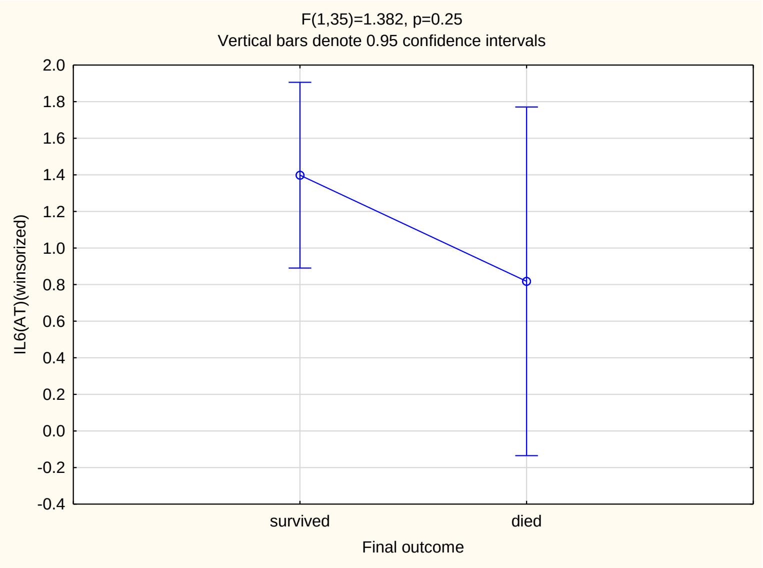

2.53) IL17 (AT)(winsorized) with Sex

2.53.1) ANOVA

	ANOVA type: III			
	Sum Sq	DF	F value	p value
(Intercept)	4.77	1	20.99	<0.01
Sex	0.11	1	0.46	0.5
Final outcome	0.4	1	1.78	0.19
Residuals	7.95	35		

2.53.2) Levene's test

	Levene's test for homogeneity of variance	
	F test	p-value
Sex	F(1, 36)=0.03	0.85
Final outcome	F(1, 36)=1.61	0.21

2.53.3) Final outcome LS means graph

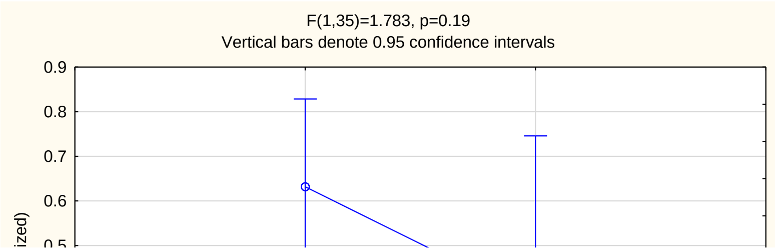

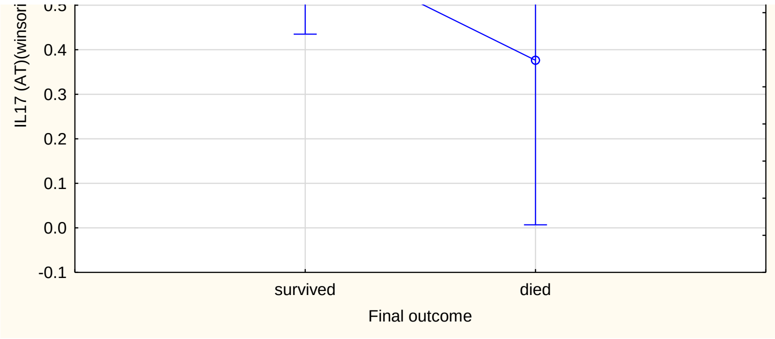

2.54) IL1b(BL)(winsorized) with Sex

2.54.1) ANOVA

	ANOVA type: III			
	Sum Sq	DF	F value	p value
(Intercept)	513.93	1	1769.64	<0.01
Sex	1.33	1	4.57	0.04
Final outcome	0.07	1	0.25	0.62
Residuals	10.16	35		

2.54.2) Levene's test

	Levene's test for homogeneity of variance	
	F test	p-value
Sex	F(1, 36)=0.41	0.53
Final outcome	F(1, 36)=2.31	0.14

2.54.3) Final outcome LS means graph

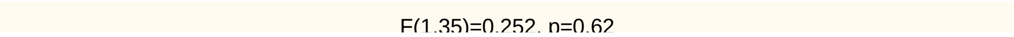

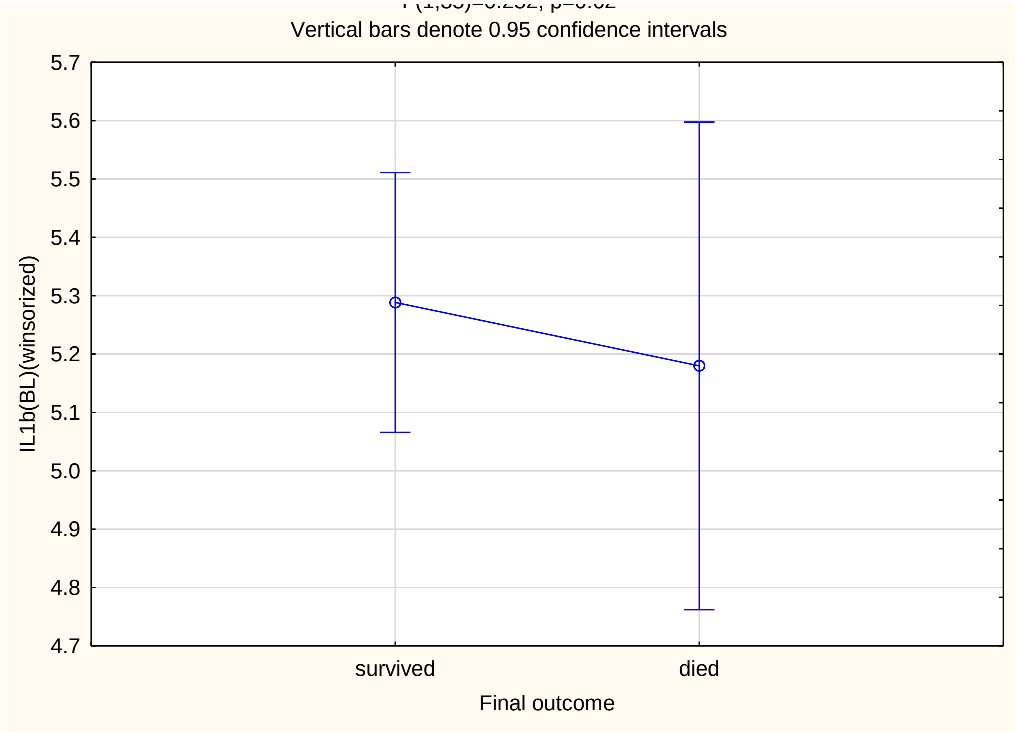

2.55) IL17(BL)(winsorized) with Sex

2.55.1) ANOVA

	ANOVA type: III			
	Sum Sq	DF	F value	p value
(Intercept)	124.55	1	16.58	<0.01
Sex	2.88	1	0.38	0.54
Final outcome	0.38	1	0.05	0.82
Residuals	262.98	35		

2.55.2) Levene's test

	Levene's test for homogeneity of variance

	F test	p-value
Sex	F(1, 36)=0.37	0.55
Final outcome	F(1, 36)=0.07	0.8

2.55.3) Final outcome LS means graph

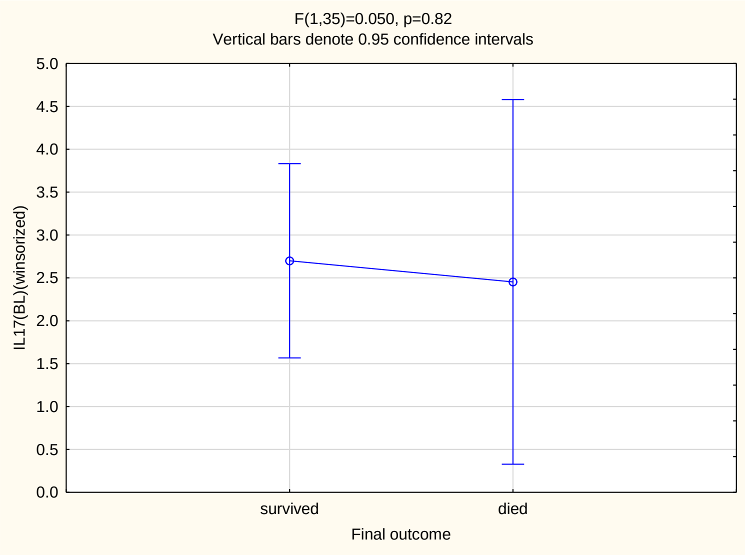

2.56) IL1b(FI AT)(winsorized) with Sex

2.56.1) ANOVA

	ANOVA type: III			
	Sum Sq	DF	F value	p value
(Intercept)	3.44	1	12.68	<0.01

Sex	0.09	1	0.32	0.58
Final outcome	0.92	1	3.4	0.07
Residuals	9.5	35		

2.56.2) Levene's test

	Levene's test for homogeneity of variance	
	F test	p-value
Sex	F(1, 36)=0.02	0.88
Final outcome	F(1, 36)=4.68	0.04

2.56.3) Final outcome LS means graph

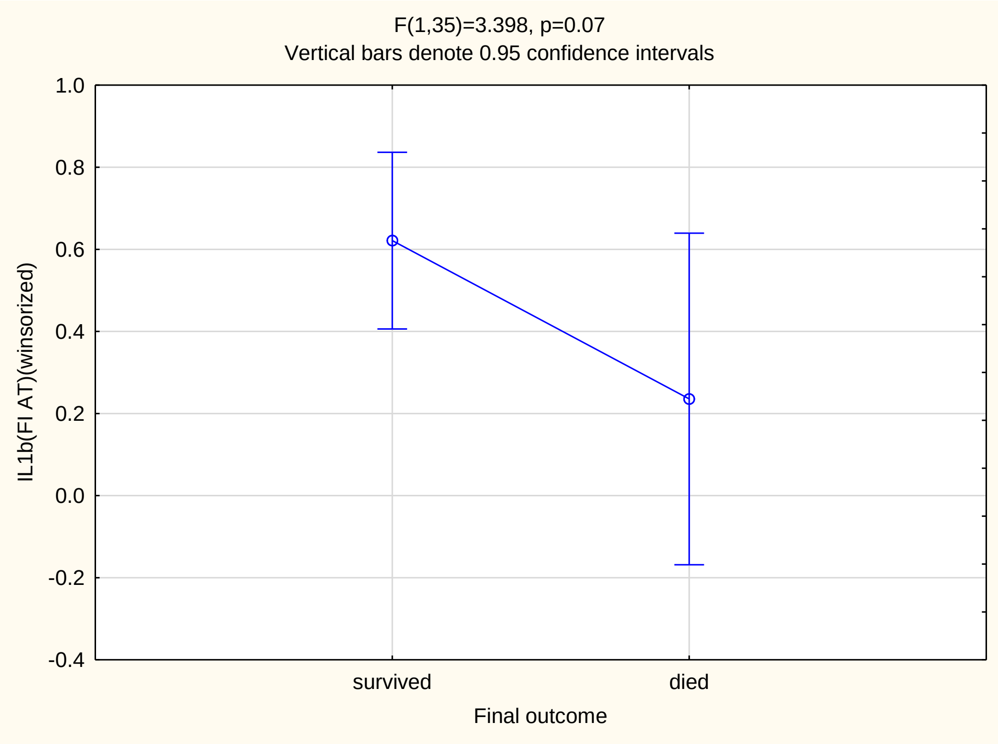

2.57) IL2 (AT) with Hypertension

2.57.1) ANOVA

	ANOVA type: III			
	Sum Sq	DF	F value	p value
(Intercept)	104499.25	1	15.96	<0.01
Hypertension	15848.91	1	2.42	0.13
Final outcome	16436.06	1	2.51	0.12
Residuals	229153.28	35		

2.57.2) Levene's test

	Levene's test for homogeneity of variance	
	F test	p-value
Hypertension	F(1, 36)=0.47	0.5
Final outcome	F(1, 36)=3.05	0.09

2.57.3) Final outcome LS means graph

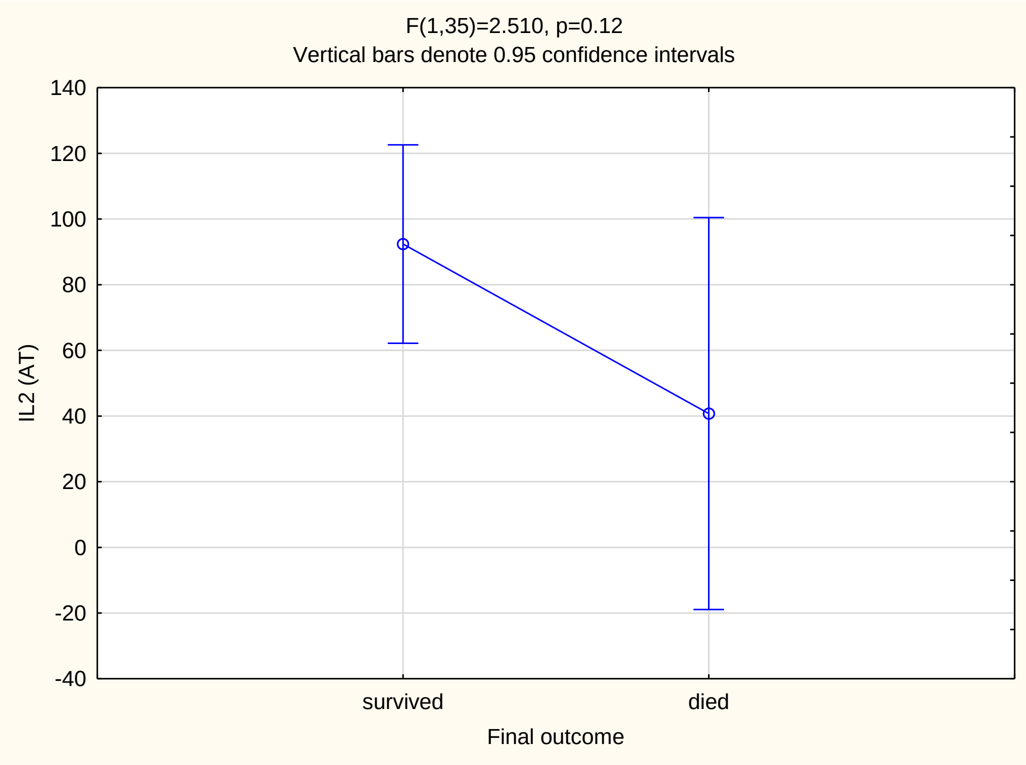

2.58) IL10(AT) with Hypertension

2.58.1) ANOVA

	ANOVA type: III			
	Sum Sq	DF	F value	p value
(Intercept)	14.48	1	21.28	<0.01
Hypertension	1.5	1	2.2	0.15
Final outcome	1.26	1	1.85	0.18
Residuals	23.82	35		

2.58.2) Levene's test

	Levene's test for homogeneity of variance	
	F test	p-value
Hypertension	F(1, 36)=0.36	0.55
Final outcome	F(1, 36)=2.08	0.16

2.58.3) Final outcome LS means graph

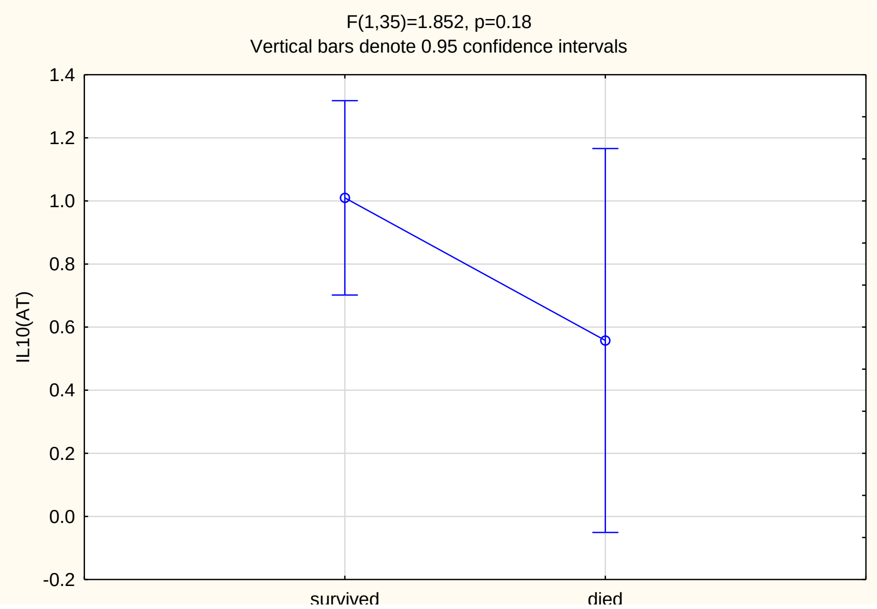

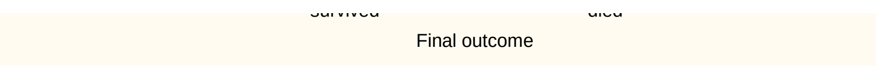

2.59) MCP1(BL) with Hypertension

2.59.1) ANOVA

	ANOVA type: III			
	Sum Sq	DF	F value	p value
(Intercept)	5804686.56	1	34.46	<0.01
Hypertension	3543.69	1	0.02	0.89
Final outcome	37046.57	1	0.22	0.64
Residuals	5895454.12	35		

2.59.2) Levene's test

	Levene's test for homogeneity of variance	
	F test	p-value
Hypertension	F(1, 36)=1.77	0.19
Final outcome	F(1, 36)=0.44	0.51

2.59.3) Final outcome LS means graph

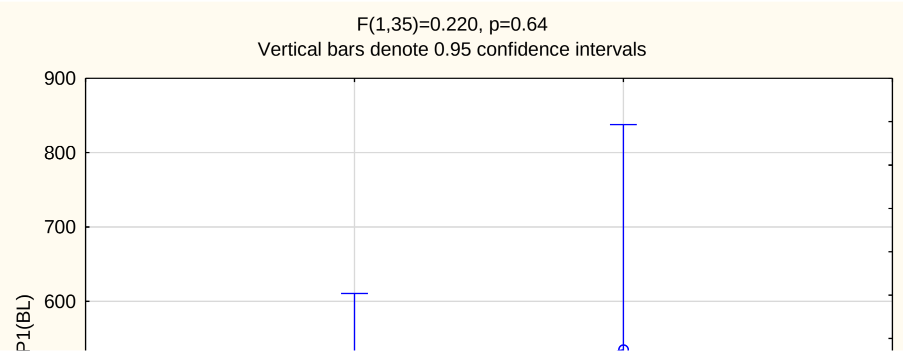

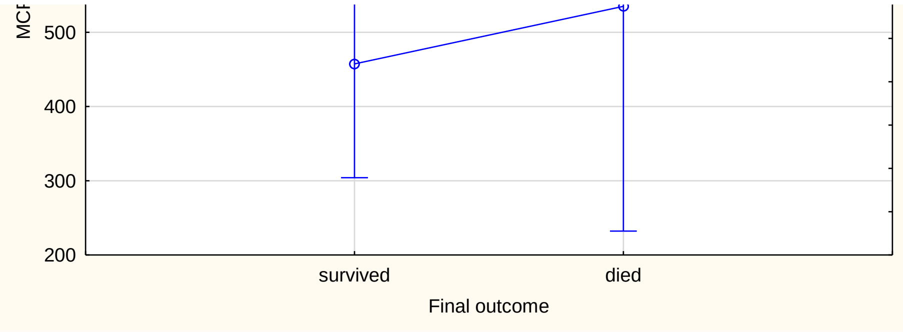

2.60) IL6(BL) with Hypertension

2.60.1) ANOVA

	ANOVA type: III			
	Sum Sq	DF	F value	p value
(Intercept)	4714.31	1	32.15	<0.01
Hypertension	1.6	1	0.01	0.92
Final outcome	166	1	1.13	0.29
Residuals	5132.66	35		

2.60.2) Levene's test

	Levene's test for homogeneity of variance	
	F test	p-value
Hypertension	F(1, 36)=0.54	0.47
Final outcome	F(1, 36)=0.60	0.44

2.60.3) Final outcome LS means graph

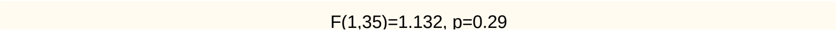

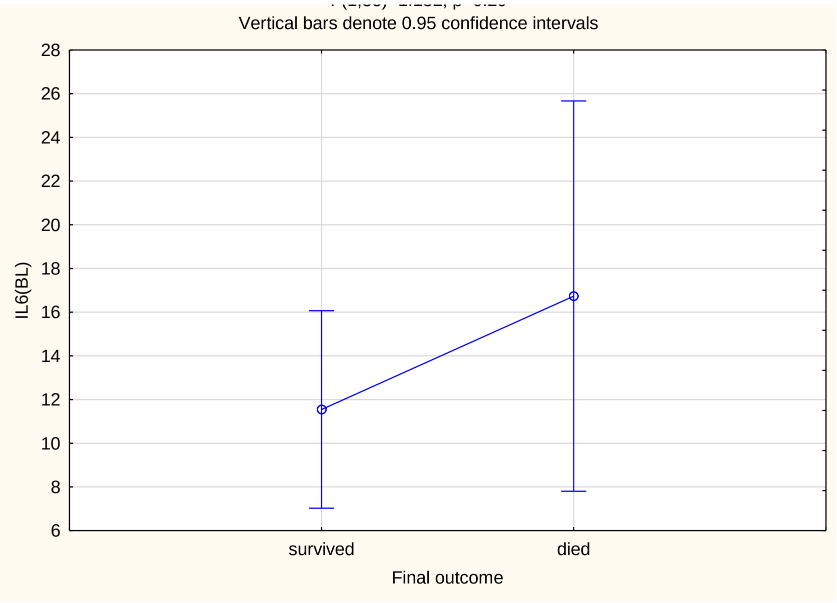

2.61) IL2(BL) with Hypertension

2.61.1) ANOVA

	ANOVA type: III			
	Sum Sq	DF	F value	p value
(Intercept)	498532.22	1	250.51785	<0.01
Hypertension	79.14	1	0.03977	0.84
Final outcome	0.1	1	0.00005	0.99
Residuals	69650.24	35		

2.61.2) Levene's test

	Levene's test for homogeneity of variance	
	F test	p-value
Hypertension	F(1, 36)=0.40	0.53

Final outcome	F(1, 36)=0.79	0.38

2.61.3) Final outcome LS means graph

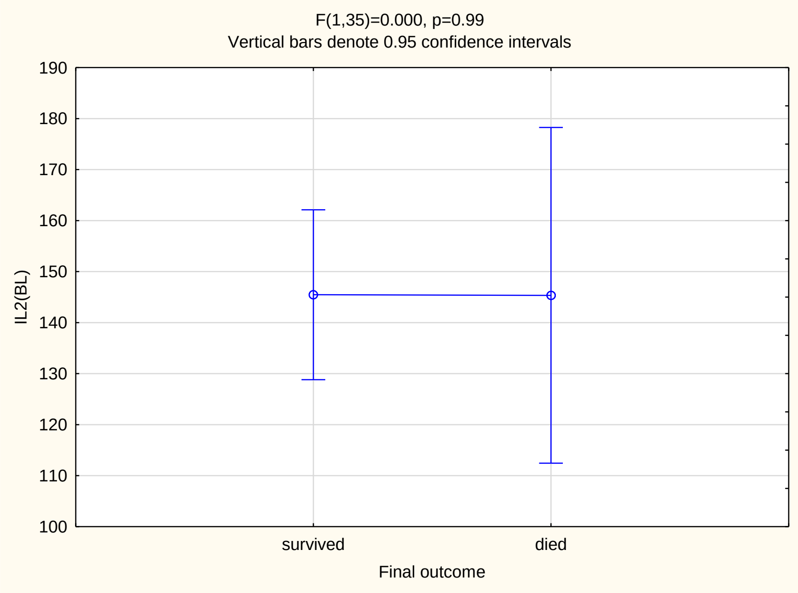

2.62) IL10(BL) with Hypertension

2.62.1) ANOVA

	ANOVA type: III			
	Sum Sq	DF	F value	p value
(Intercept)	889.58	1	72.99	<0.01
Hypertension	13.95	1	1.14	0.29
Final outcome	66.36	1	5.44	0.03

Residuals	426.56	35		

2.62.2) Levene's test

	Levene's test for homogeneity of variance	
	F test	p-value
Hypertension	F(1, 36)=0.05	0.82
Final outcome	F(1, 36)=8.59	<0.01

2.62.3) Final outcome LS means graph

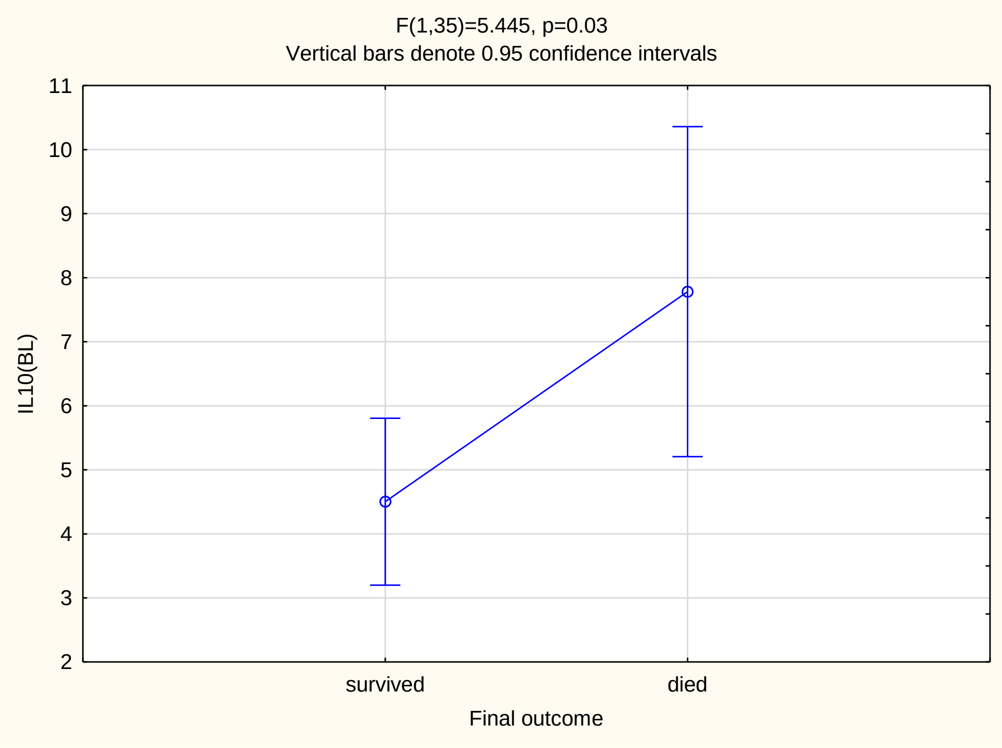

2.62.4) Welch tests

2.62.4.1) Final outcome adjusted means plot

2.63) TNFa(BL) with Hypertension

2.63.1) ANOVA

	ANOVA type: III			
	Sum Sq	DF	F value	p value
(Intercept)	1693.41	1	79.16	<0.01
Hypertension	96.48	1	4.51	0.04
Final outcome	1.15	1	0.05	0.82
Residuals	748.69	35		

2.63.2) Levene's test

	Levene's test for homogeneity of variance	
	F test	p-value
Hypertension	F(1, 36)=6.52	0.02
Final outcome	F(1, 36)=0.58	0.45

2.63.3) Final outcome LS means graph

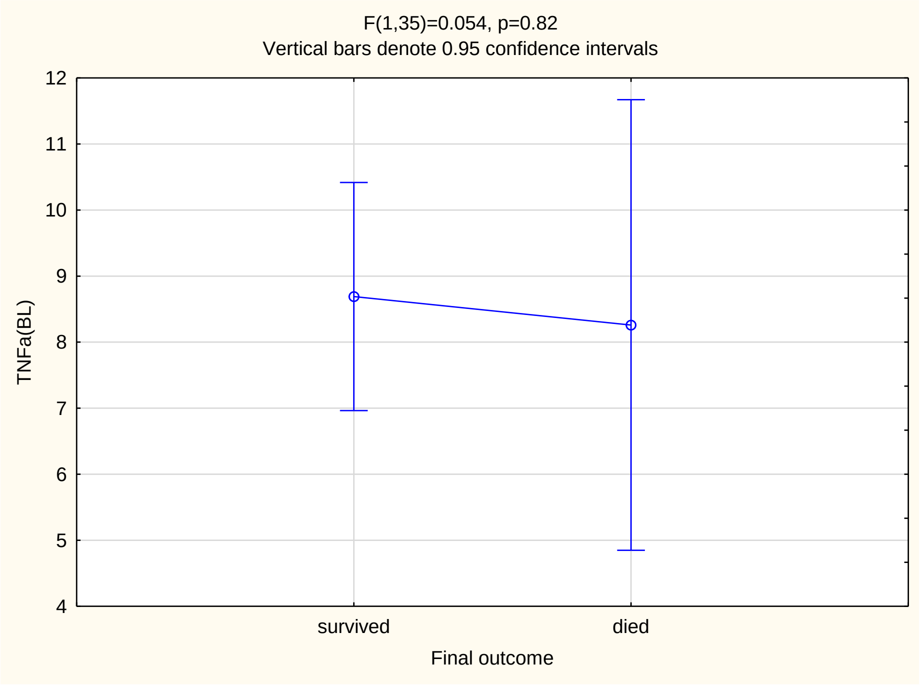

2.64) TNFa(AT)(winsorized) with Hypertension

2.64.1) ANOVA

	ANOVA type: III			
	Sum Sq	DF	F value	p value
(Intercept)	1.9	1	18.85	<0.01
Hypertension	0.28	1	2.77	0.1
Final outcome	0.28	1	2.74	0.11
Residuals	3.52	35		

2.64.2) Levene's test

	Levene's test for homogeneity of variance	
	F test	p-value
Hypertension	F(1, 36)=0.37	0.55
Final outcome	F(1, 36)=4.89	0.03

2.64.3) Final outcome LS means graph

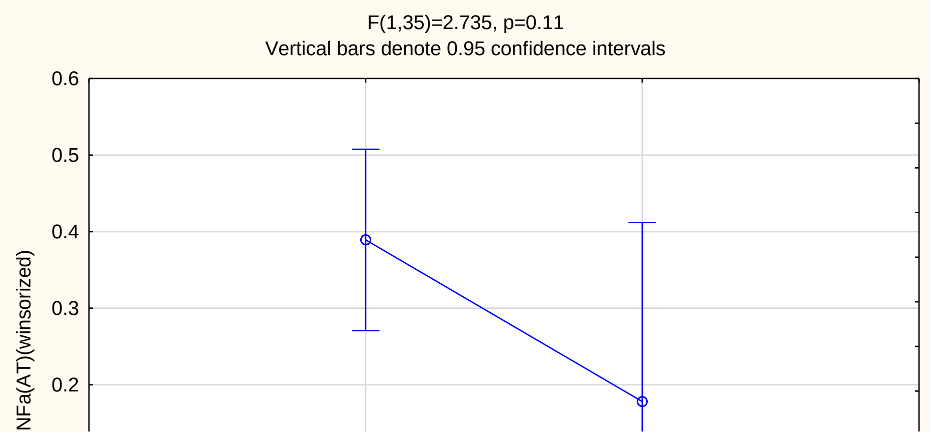

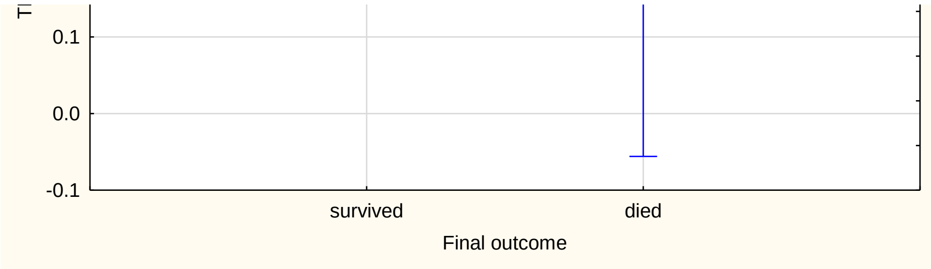

2.65) MCP1(AT)(winsorized) with Hypertension

2.65.1) ANOVA

	ANOVA type: III			
	Sum Sq	DF	F value	p value
(Intercept)	2788	1	19.81	<0.01
Hypertension	380.1	1	2.7	0.11
Final outcome	279.07	1	1.98	0.17
Residuals	4926.23	35		

2.65.2) Levene's test

	Levene's test for homogeneity of variance	
	F test	p-value
Hypertension	F(1, 36)=0.33	0.57
Final outcome	F(1, 36)=0.93	0.34

2.65.3) Final outcome LS means graph

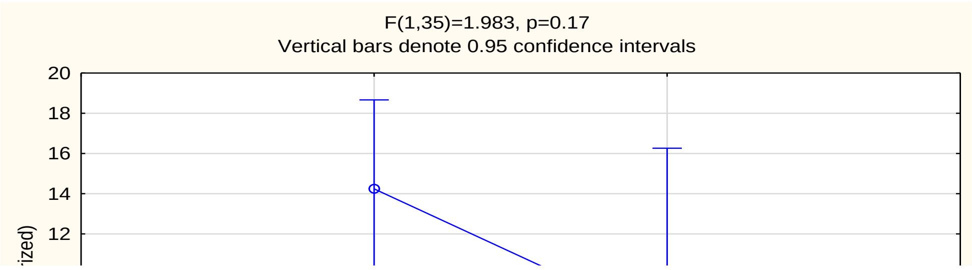

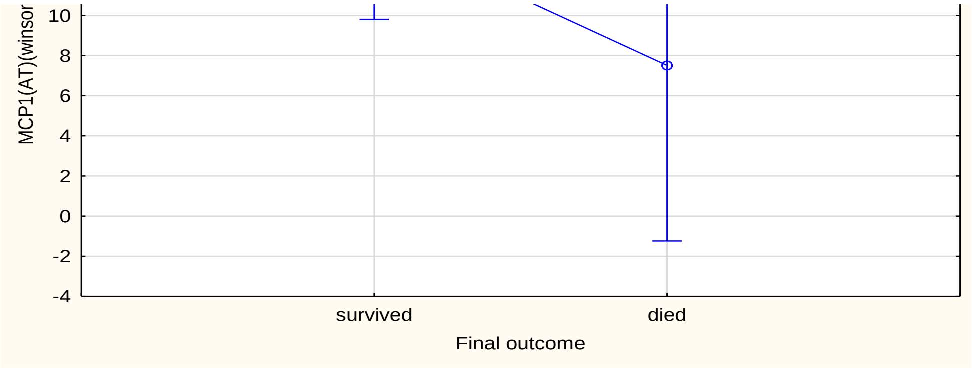

2.66) IL6(AT)(winsorized) with Hypertension

2.66.1) ANOVA

	ANOVA type: III			
	Sum Sq	DF	F value	p value
(Intercept)	26.44	1	18.3	<0.01
Hypertension	2.39	1	1.66	0.21
Final outcome	2.69	1	1.86	0.18
Residuals	50.55	35		

2.66.2) Levene's test

	Levene's test for homogeneity of variance	
	F test	p-value
Hypertension	F(1, 36)=0.00	0.95
Final outcome	F(1, 36)=2.13	0.15

2.66.3) Final outcome LS means graph

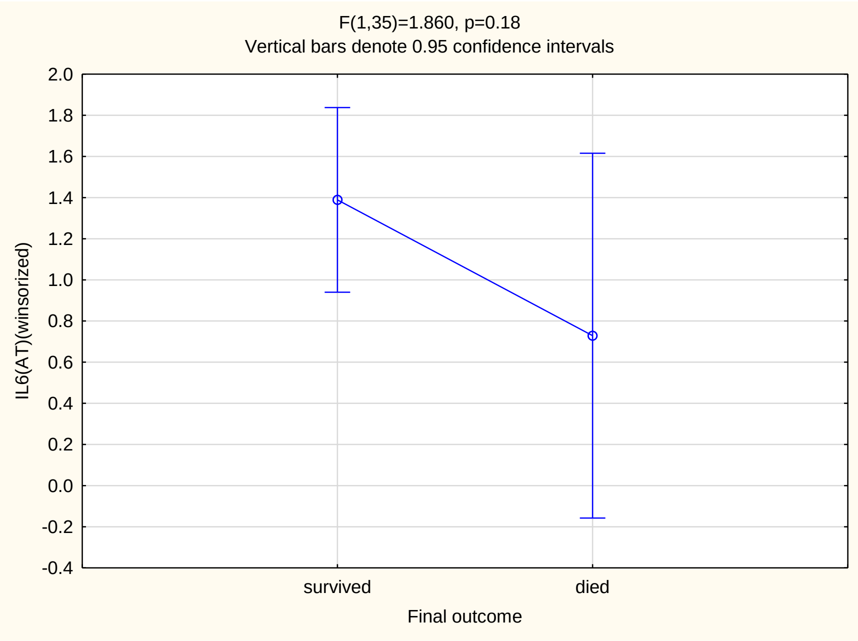

2.67) IL17 (AT)(winsorized) with Hypertension

2.67.1) ANOVA

	ANOVA type: III			
	Sum Sq	DF	F value	p value
(Intercept)	6.12	1	27.91	<0.01
Hypertension	0.38	1	1.72	0.2
Final outcome	0.47	1	2.14	0.15
Residuals	7.67	35		

2.67.2) Levene's test

	Levene's test for homogeneity of variance	
	F test	p-value
Hypertension	F(1, 36)=0.46	0.5
Final outcome	F(1, 36)=1.61	0.21

2.67.3) Final outcome LS means graph

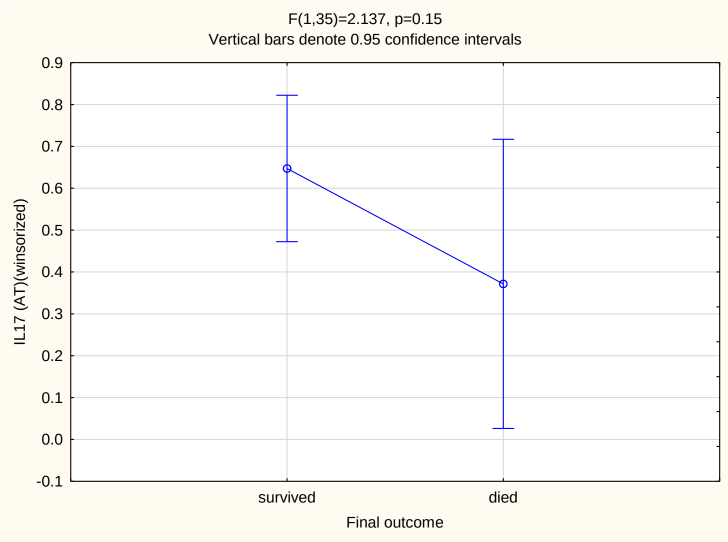

2.68) IL1b(BL)(winsorized) with Hypertension

2.68.1) ANOVA

	ANOVA type: III			
	Sum Sq	DF	F value	p value

(Intercept)	683.623	1	2097.19	<0.01
Hypertension	0.084	1	0.26	0.62
Final outcome	0.005	1	0.01	0.9
Residuals	11.409	35		

2.68.2) Levene's test

	Levene's test for homogeneity of variance	
	F test	p-value
Hypertension	F(1, 36)=0.04	0.84
Final outcome	F(1, 36)=2.31	0.14

2.68.3) Final outcome LS means graph

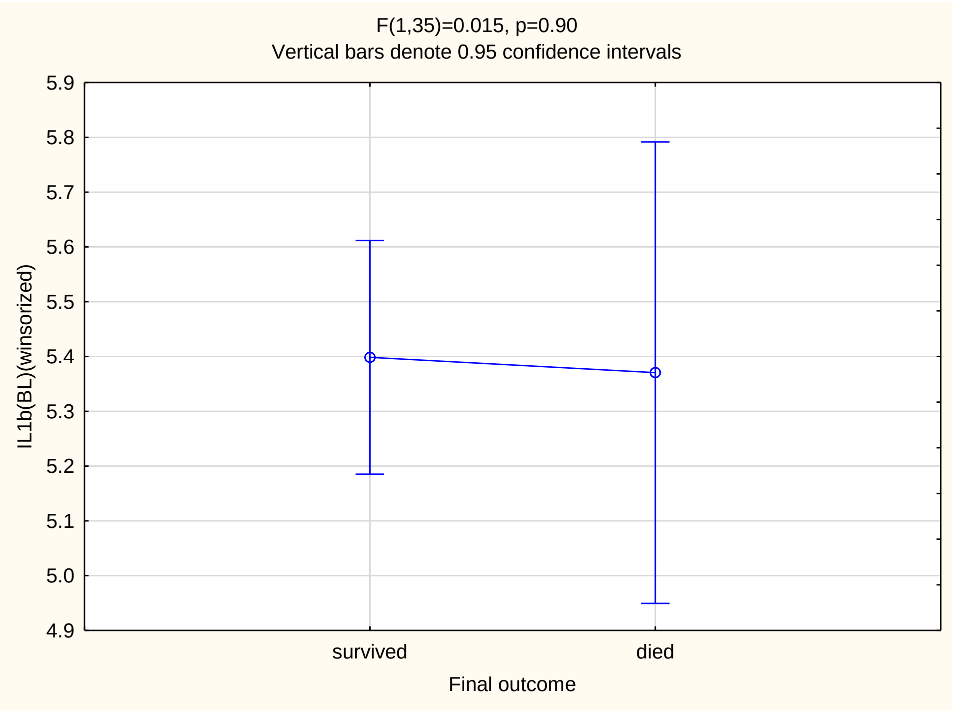

2.69) IL17(BL)(winsorized) with Hypertension

2.69.1) ANOVA

	ANOVA type: III			
	Sum Sq	DF	F value	p value
(Intercept)	194.33	1	25.894	<0.01
Hypertension	3.2	1	0.427	0.52
Final outcome	0.01	1	0.002	0.97
Residuals	262.67	35		

2.69.2) Levene's test

	Levene's test for homogeneity of variance	
	F test	p-value
Hypertension	F(1, 36)=0.46	0.5
Final outcome	F(1, 36)=0.07	0.8

2.69.3) Final outcome LS means graph

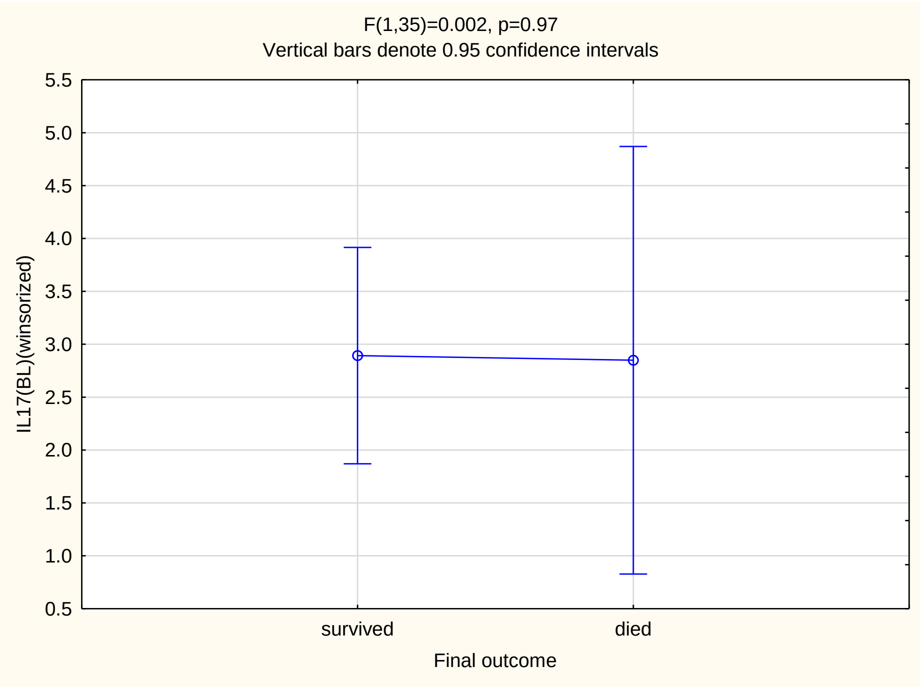

2.70) IL1b(FI AT)(winsorized) with Hypertension

2.70.1) ANOVA

	ANOVA type: III			
	Sum Sq	DF	F value	p value
(Intercept)	3.94	1	16.11	<0.01
Hypertension	1.03	1	4.21	0.05
Final outcome	1.15	1	4.71	0.04
Residuals	8.56	35		

2.70.2) Levene's test

	Levene's test for homogeneity of variance	
	F test	p-value
Hypertension	F(1, 36)=0.52	0.48
Final outcome	F(1, 36)=4.68	0.04

2.70.3) Final outcome LS means graph

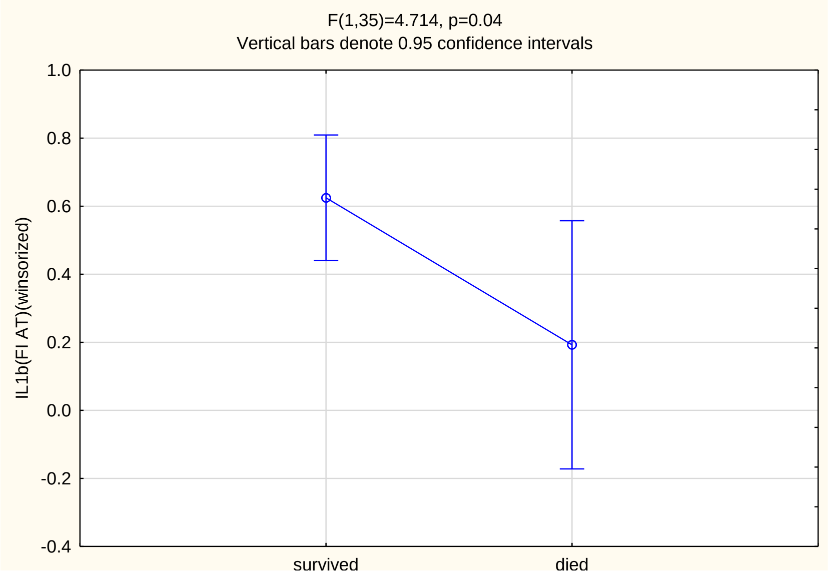

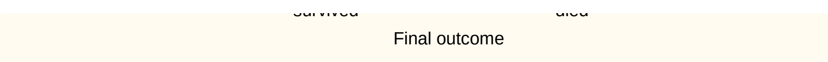

2.71) IL2 (AT) with Diabetes

2.71.1) ANOVA

	ANOVA type: III			
	Sum Sq	DF	F value	p value
(Intercept)	132875.88	1	19.21	<0.01
Diabetes	2955.54	1	0.43	0.52
Final outcome	13555.35	1	1.96	0.17
Residuals	242046.65	35		

2.71.2) Levene's test

	Levene's test for homogeneity of variance	
	F test	p-value
Diabetes	F(1, 36)=0.29	0.59
Final outcome	F(1, 36)=3.05	0.09

2.71.3) Final outcome LS means graph

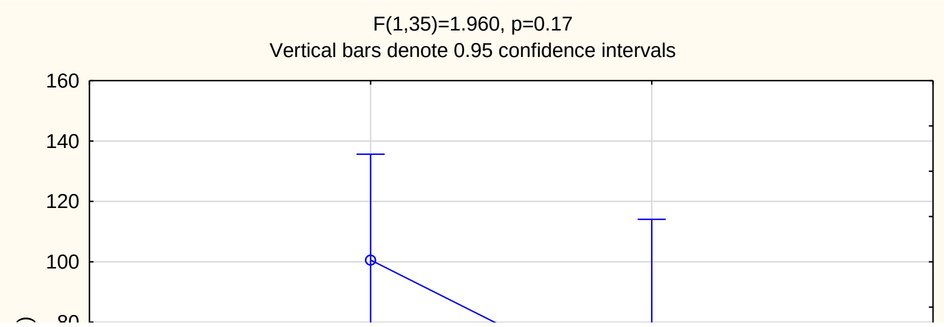

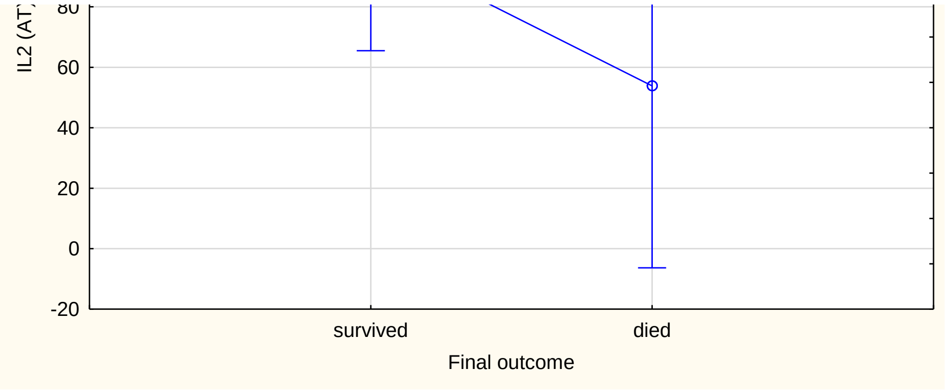

2.72) IL10(AT) with Diabetes

2.72.1) ANOVA

	ANOVA type: III			
	Sum Sq	DF	F value	p value
(Intercept)	16.72	1	23.17	<0.01
Diabetes	0.06	1	0.08	0.78
Final outcome	0.94	1	1.3	0.26
Residuals	25.26	35		

2.72.2) Levene's test

	Levene's test for homogeneity of variance	
	F test	p-value
Diabetes	F(1, 36)=0.16	0.69
Final outcome	F(1, 36)=2.08	0.16

2.72.3) Final outcome LS means graph

F(1,35)=1.305, p=0.26
Vertical bars denote 0.95 confidence intervals

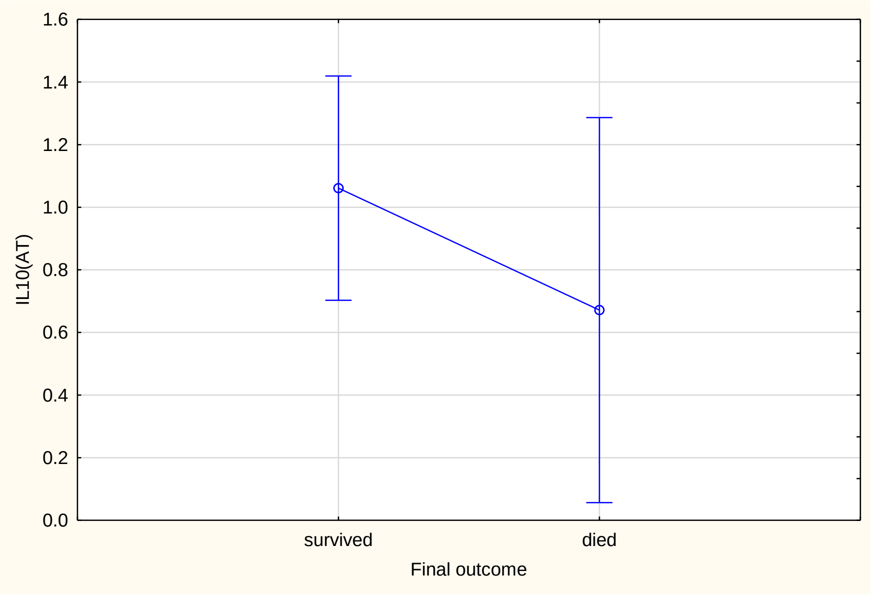

2.73) MCP1(BL) with Diabetes

2.73.1) ANOVA

	ANOVA type: III			
	Sum Sq	DF	F value	p value
(Intercept)	5875364.04	1	35.09	<0.01
Diabetes	37902.16	1	0.23	0.64
Final outcome	31215.72	1	0.19	0.67
Residuals	5861095.65	35		

2.73.2) Levene's test

	Levene's test for homogeneity of variance	
	F test	p-value

Diabetes	F(1, 36)=1.90	0.18
Final outcome	F(1, 36)=0.44	0.51

2.73.3) Final outcome LS means graph

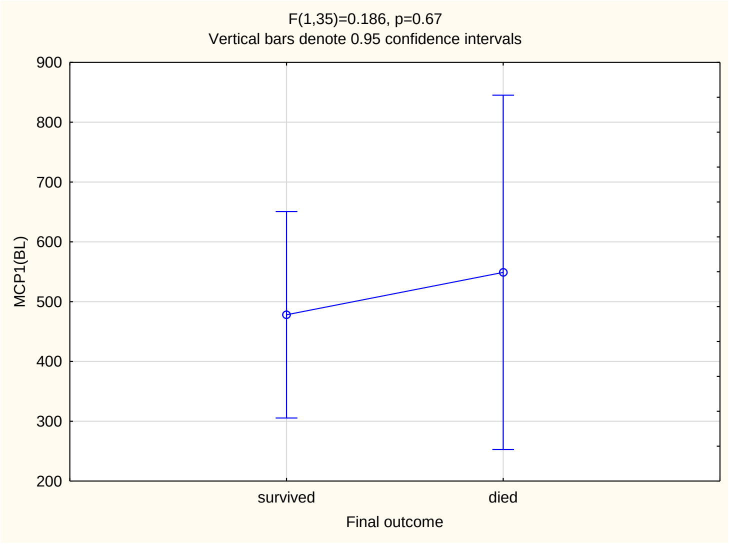

2.74) IL6(BL) with Diabetes

2.74.1) ANOVA

	ANOVA type: III			
	Sum Sq	DF	F value	p value
(Intercept)	5284.04	1	38.01	<0.01
Diabetes	268.43	1	1.93	0.17

Final outcome	120.34	1	0.87	0.36
Residuals	4865.83	35		

2.74.2) Levene's test

	Levene's test for homogeneity of variance	
	F test	p-value
Diabetes	F(1, 36)=8.51	<0.01
Final outcome	F(1, 36)=0.60	0.44

2.74.3) Final outcome LS means graph

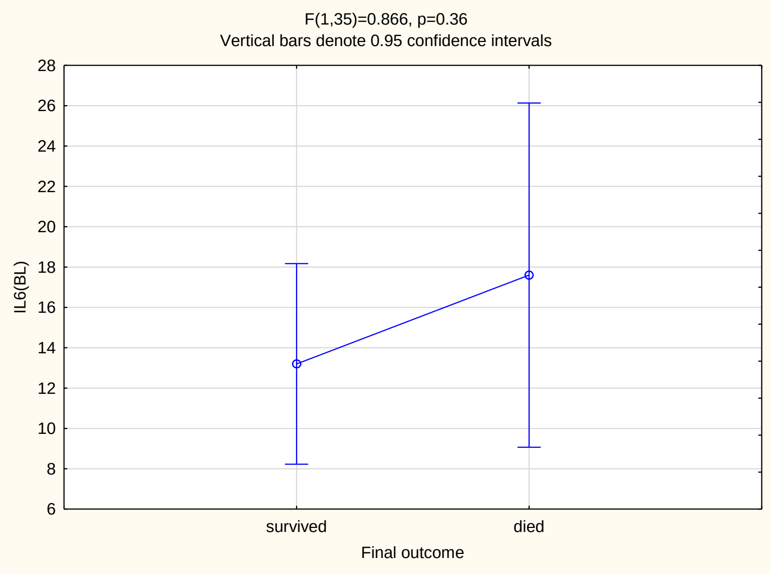

2.75) IL2(BL) with Diabetes

2.75.1) ANOVA

	ANOVA type: III			
	Sum Sq	DF	F value	p value
(Intercept)	522092.22	1	319.34	<0.01
Diabetes	12506.8	1	7.65	<0.01
Final outcome	267.5	1	0.16	0.69
Residuals	57222.57	35		

2.75.2) Levene's test

	Levene's test for homogeneity of variance	
	F test	p-value
Diabetes	F(1, 36)=2.96	0.09
Final outcome	F(1, 36)=0.79	0.38

2.75.3) Final outcome LS means graph

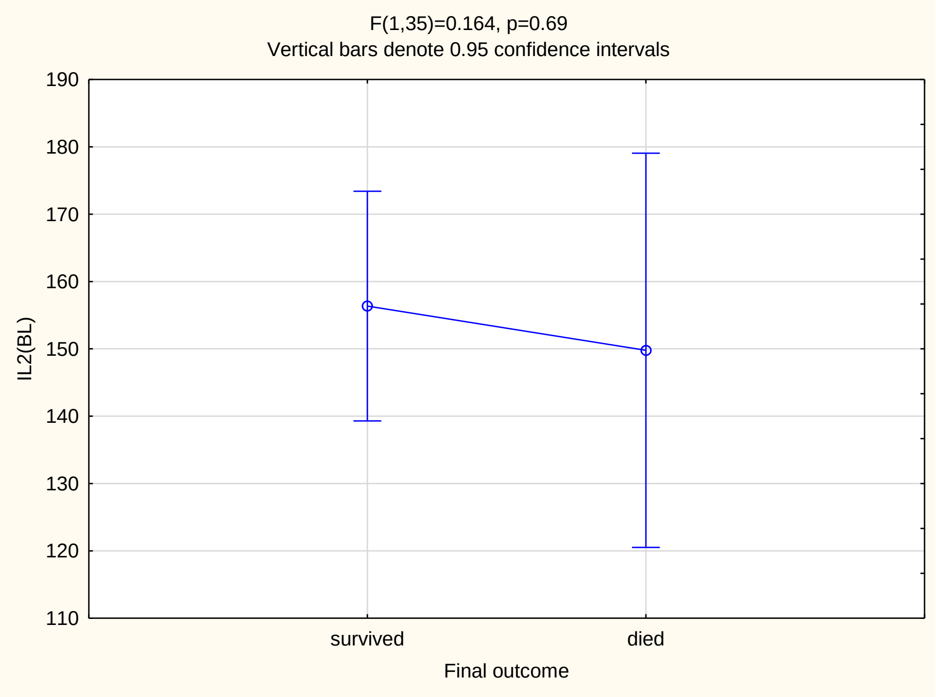

2.76) IL10(BL) with Diabetes

2.76.1) ANOVA

	ANOVA type: III			
	Sum Sq	DF	F value	p value
(Intercept)	787.195013	1	62.5452564	<0.01
Diabetes	0.000001	1	0.0000001	1
Final outcome	57.742818	1	4.5878585	0.04
Residuals	440.510233	35		

2.76.2) Levene's test

	Levene's test for homogeneity of variance	
	F test	p-value
Diabetes	F(1, 36)=0.08	0.78
Final outcome	F(1, 36)=8.59	<0.01

2.76.3) Final outcome LS means graph

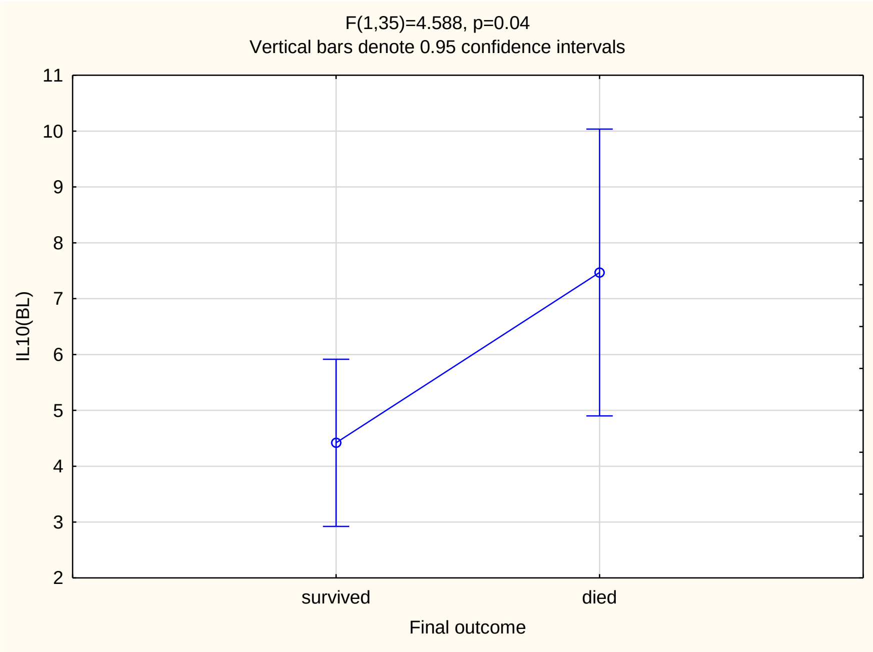

2.76.4) Welch tests

2.76.4.1) Final outcome adjusted means plot

2.77) TNFa(BL) with Diabetes

2.77.1) ANOVA

	ANOVA type: III			
	Sum Sq	DF	F value	p value
(Intercept)	2012.6	1	88.42	<0.01
Diabetes	48.54	1	2.13	0.15
Final outcome	0.23	1	0.01	0.92
Residuals	796.62	35		

2.77.2) Levene's test

	Levene's test for homogeneity of variance	
	F test	p-value
Diabetes	F(1, 36)=2.90	0.1
Final outcome	F(1, 36)=0.58	0.45

2.77.3) Final outcome LS means graph

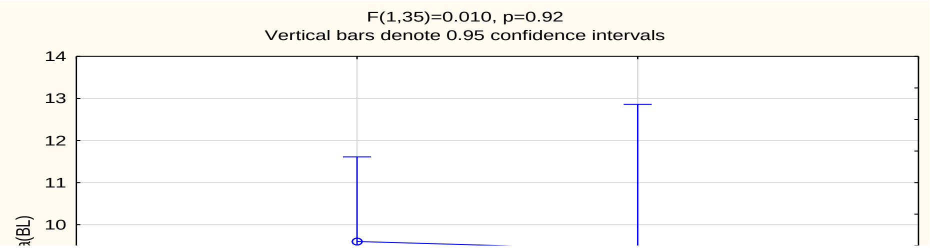

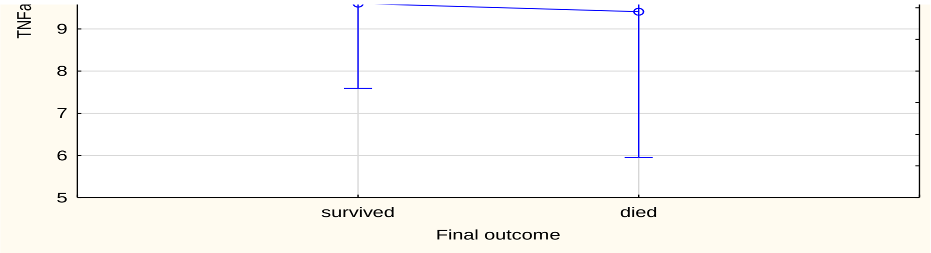

2.78) TNFa(AT)(winsorized) with Diabetes

2.78.1) ANOVA

	ANOVA type: III			
	Sum Sq	DF	F value	p value
(Intercept)	2.28	1	21.09	<0.01
Diabetes	0.01	1	0.12	0.73
Final outcome	0.21	1	1.96	0.17
Residuals	3.79	35		

2.78.2) Levene's test

	Levene's test for homogeneity of variance	
	F test	p-value
Diabetes	F(1, 36)=0.80	0.38
Final outcome	F(1, 36)=4.89	0.03

2.78.3) Final outcome LS means graph

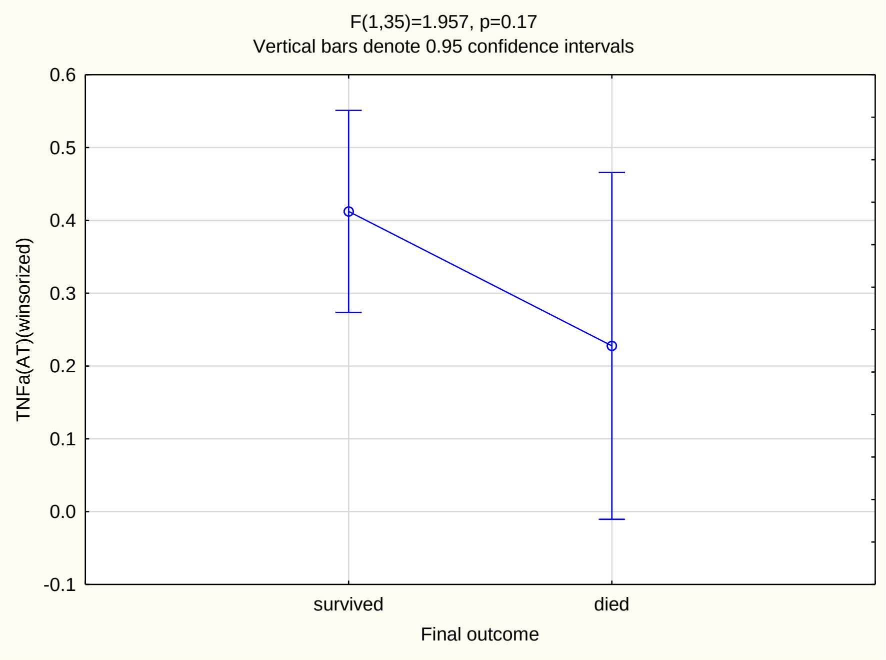

2.79) MCP1(AT)(winsorized) with Diabetes

2.79.1) ANOVA

	ANOVA type: III			
	Sum Sq	DF	F value	p value
(Intercept)	3851.98	1	26.87	<0.01
Diabetes	289.22	1	2.02	0.16
Final outcome	255.94	1	1.79	0.19
Residuals	5017.12	35		

2.79.2) Levene's test

	Levene's test for homogeneity of variance	
	F test	p-value
Diabetes	F(1, 36)=4.02	0.05
Final outcome	F(1, 36)=0.93	0.34

2.79.3) Final outcome LS means graph

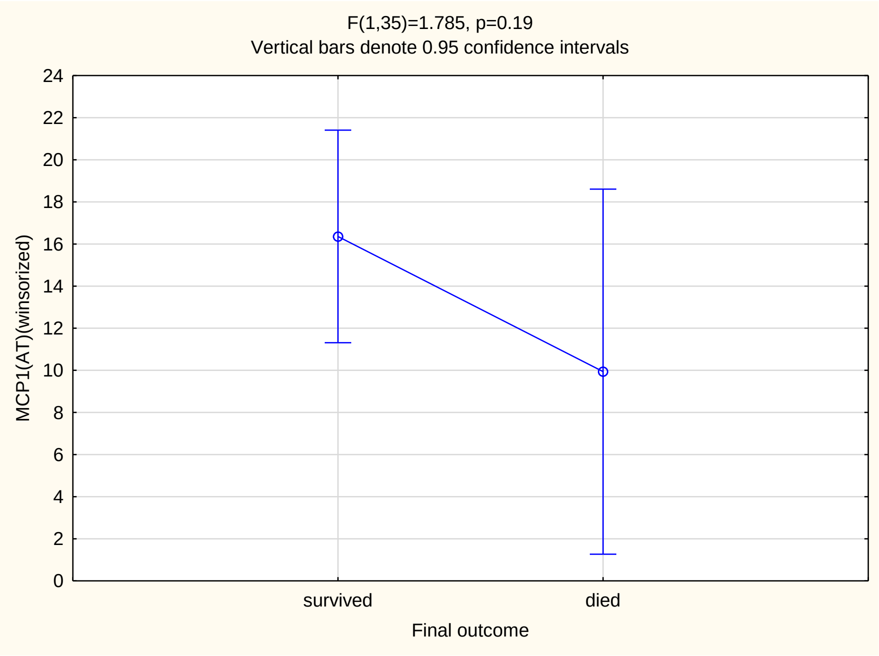

2.80) IL6(AT)(winsorized) with Diabetes

2.80.1) ANOVA

	ANOVA type: III			
	Sum Sq	DF	F value	p value

(Intercept)	32.49	1	21.82	<0.01
Diabetes	0.84	1	0.56	0.46
Final outcome	2.33	1	1.57	0.22
Residuals	52.11	35		

2.80.2) Levene's test

	Levene's test for homogeneity of variance	
	F test	p-value
Diabetes	F(1, 36)=1.17	0.29
Final outcome	F(1, 36)=2.13	0.15

2.80.3) Final outcome LS means graph

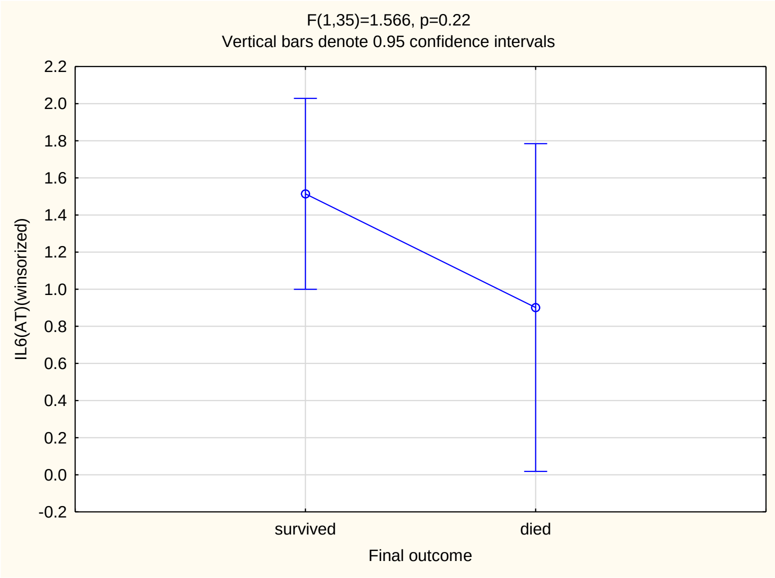

2.81) IL17 (AT)(winsorized) with Diabetes

2.81.1) ANOVA

	ANOVA type: III			
	Sum Sq	DF	F value	p value
(Intercept)	6.35	1	27.66	<0.01
Diabetes	0.01	1	0.05	0.82
Final outcome	0.33	1	1.45	0.24
Residuals	8.04	35		

2.81.2) Levene's test

	Levene's test for homogeneity of variance	
	F test	p-value
Diabetes	F(1, 36)=1.45	0.24
Final outcome	F(1, 36)=1.61	0.21

2.81.3) Final outcome LS means graph

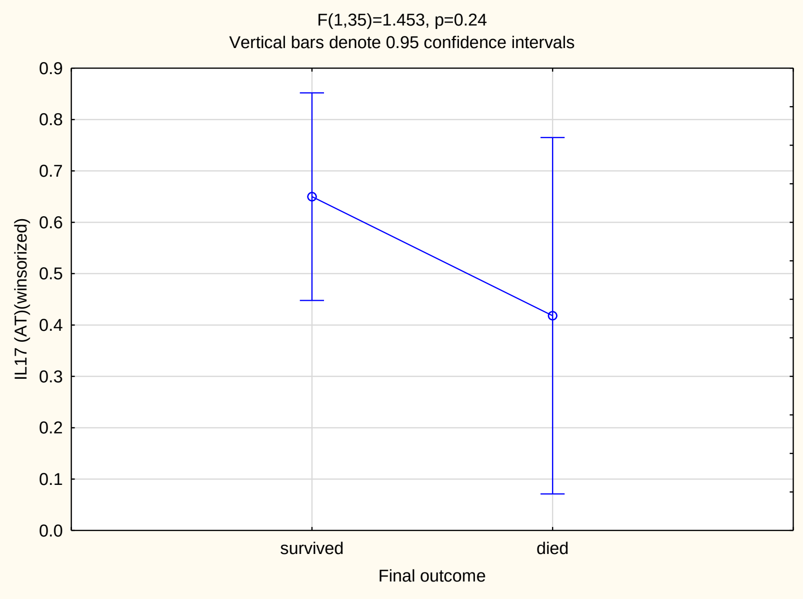

2.82) IL1b(BL)(winsorized) with Diabetes

2.82.1) ANOVA

	ANOVA type: III			
	Sum Sq	DF	F value	p value
(Intercept)	654.93	1	2088.73	<0.01
Diabetes	0.52	1	1.65	0.21
Final outcome	0.04	1	0.14	0.71
Residuals	10.97	35		

2.82.2) Levene's test

	Levene's test for homogeneity of variance	
	F test	p-value
Diabetes	F(1, 36)=0.56	0.46
Final outcome	F(1, 36)=2.31	0.14

2.82.3) Final outcome LS means graph

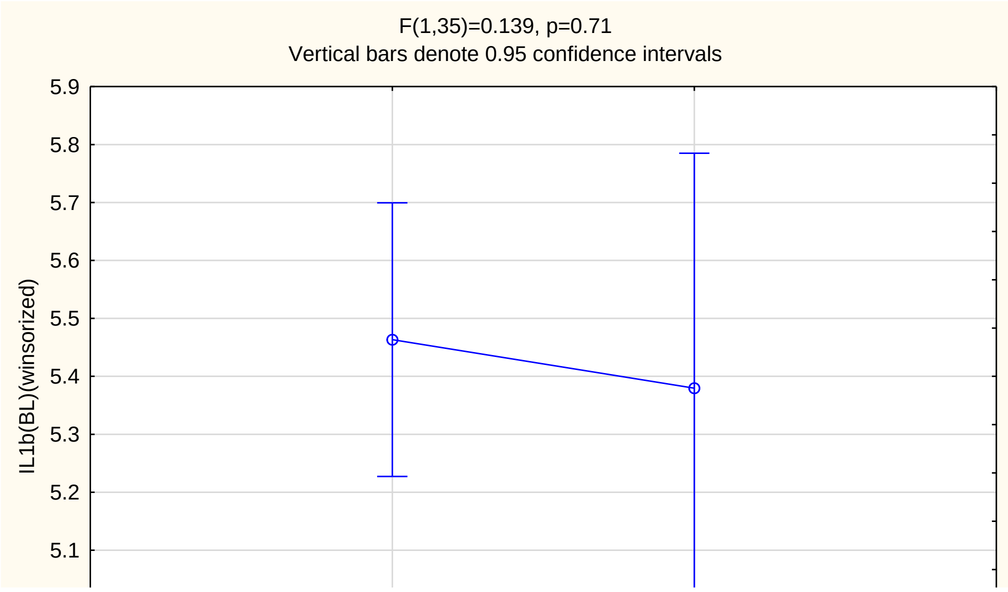

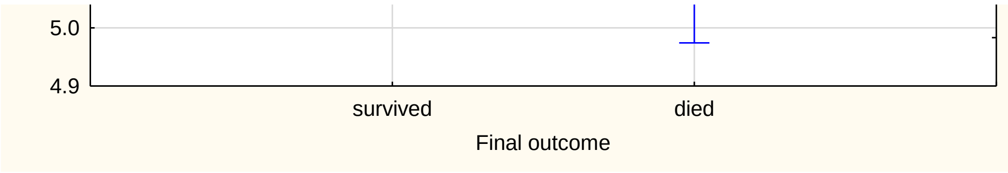

2.83) IL17(BL)(winsorized) with Diabetes

2.83.1) ANOVA

	ANOVA type: III			
	Sum Sq	DF	F value	p value
(Intercept)	175.29	1	23.09	<0.01
Diabetes	0.16	1	0.02	0.89
Final outcome	0.19	1	0.03	0.88
Residuals	265.71	35		

2.83.2) Levene's test

	Levene's test for homogeneity of variance	
	F test	p-value
Diabetes	F(1, 36)=0.39	0.54
Final outcome	F(1, 36)=0.07	0.8

2.83.3) Final outcome LS means graph

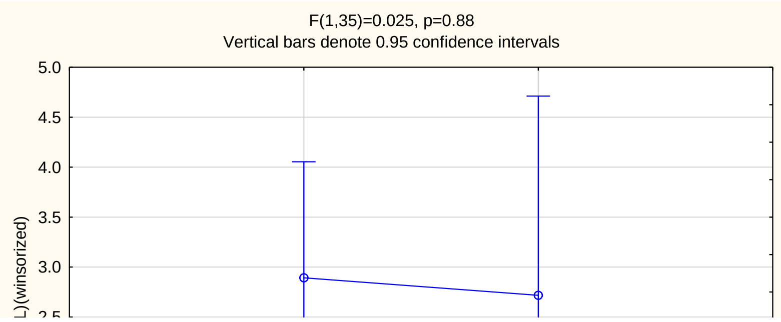

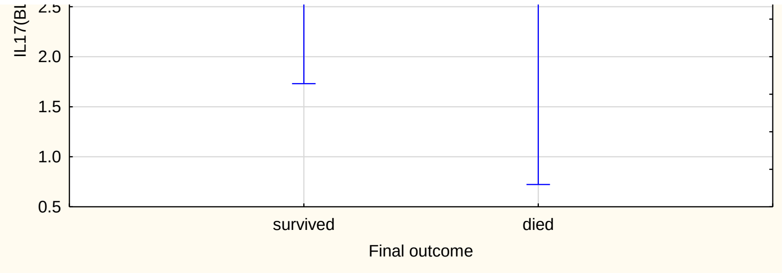

2.84) IL1b(FI AT)(winsorized) with Diabetes

2.84.1) ANOVA

	ANOVA type: III			
	Sum Sq	DF	F value	p value
(Intercept)	5.62	1	21.18	<0.01
Diabetes	0.3	1	1.12	0.3
Final outcome	0.99	1	3.71	0.06
Residuals	9.29	35		

2.84.2) Levene's test

	Levene's test for homogeneity of variance	
	F test	p-value
Diabetes	F(1, 36)=0.64	0.43
Final outcome	F(1, 36)=4.68	0.04

2.84.3) Final outcome LS means graph

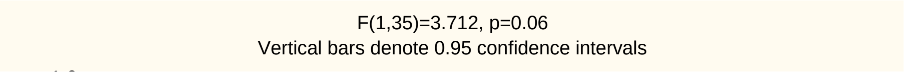

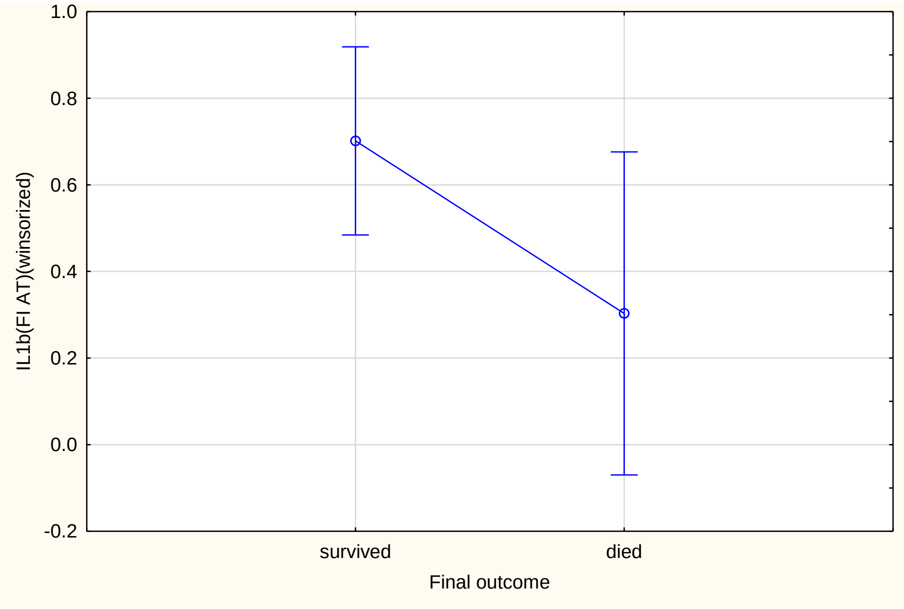

2.85) IL2 (AT) with Age

2.85.1) ANOVA

	ANOVA type: III			
	Sum Sq	DF	F value	p value
(Intercept)	28734.29	1	4.25	0.05
Age	8208.35	1	1.21	0.28
Final outcome	7505.69	1	1.11	0.3
Residuals	236793.84	35		

2.85.2) Levene's test

	Levene's test for homogeneity of variance	
	F test	p-value

Final outcome	F(1, 36)=3.05	0.09

2.85.3) Final outcome LS means graph

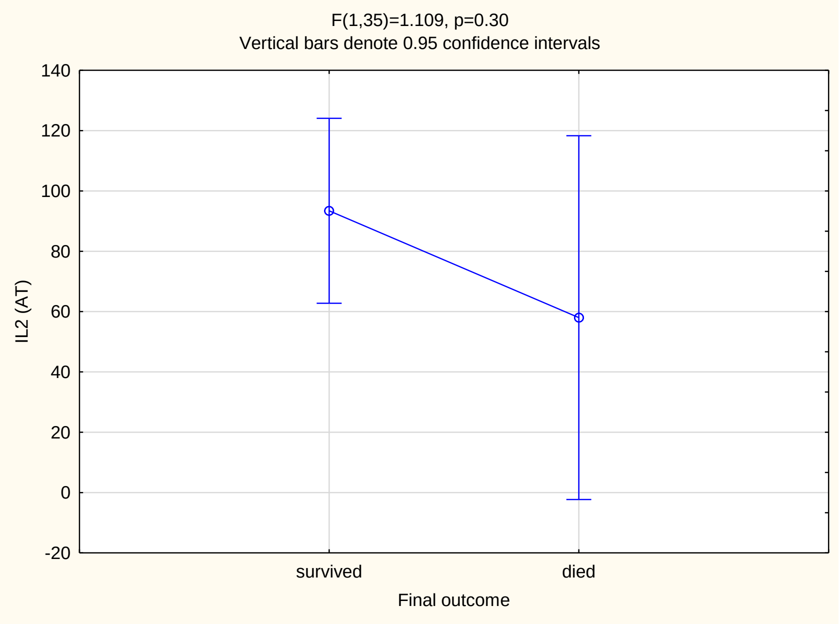

2.86) IL10(AT) with Age

2.86.1) ANOVA

	ANOVA type: III			
	Sum Sq	DF	F value	p value
(Intercept)	3.67	1	5.28	0.03
Age	1	1	1.44	0.24
Final outcome	0.48	1	0.69	0.41

Residuals	24.31	35		

2.86.2) Levene's test

	Levene's test for homogeneity of variance	
	F test	p-value
Final outcome	F(1, 36)=2.08	0.16

2.86.3) Final outcome LS means graph

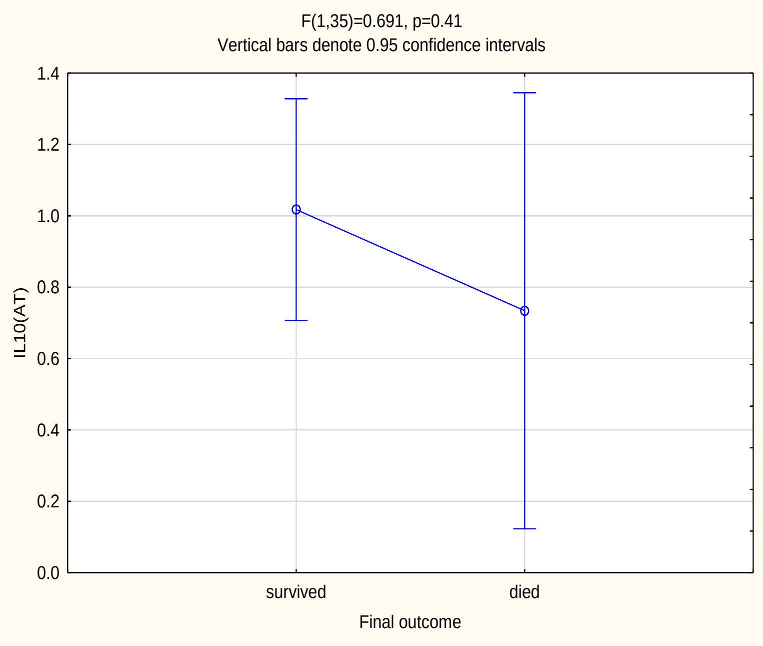

2.87) MCP1(BL) with Age

2.87.1) ANOVA

	ANOVA type: III			
	Sum Sq	DF	F value	p value
(Intercept)	211734.21	1	1.26	0.27
Age	9058.12	1	0.05	0.82
Final outcome	31271.8	1	0.19	0.67
Residuals	5889939.68	35		

2.87.2) Levene's test

	Levene's test for homogeneity of variance	
	F test	p-value
Final outcome	F(1, 36)=0.44	0.51

2.87.3) Final outcome LS means graph

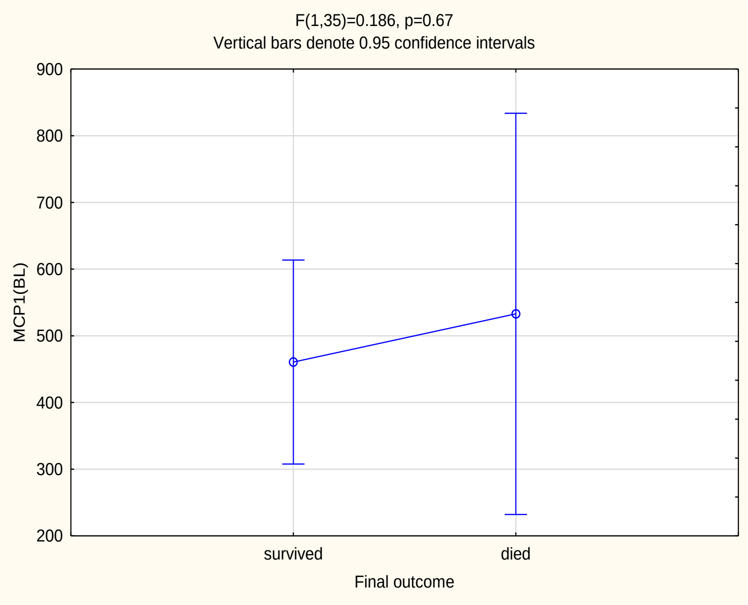

2.88) IL6(BL) with Age

2.88.1) ANOVA

	ANOVA type: III			
	Sum Sq	DF	F value	p value
(Intercept)	182.45	1	1.24	0.27
Age	5.21	1	0.04	0.85
Final outcome	152.82	1	1.04	0.31
Residuals	5129.05	35		

2.88.2) Levene's test

	Levene's test for homogeneity of variance	
	F test	p-value
Final outcome	F(1, 36)=0.60	0.44

2.88.3) Final outcome LS means graph

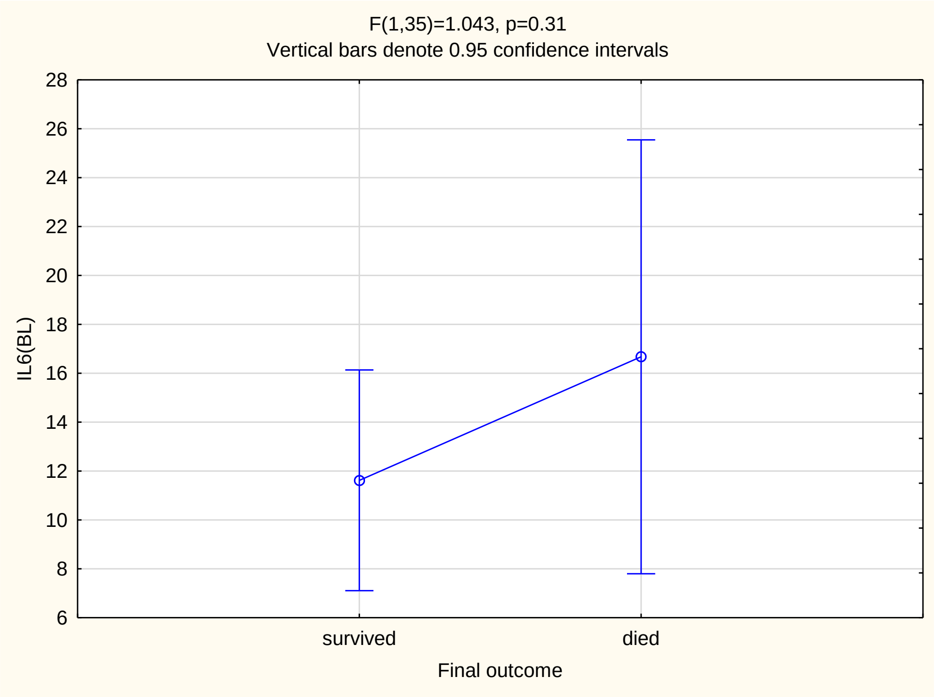

2.89) IL2(BL) with Age

2.89.1) ANOVA

	ANOVA type: III			
	Sum Sq	DF	F value	p value
(Intercept)	25477.9	1	12.7885	<0.01
Age	0.83	1	0.0004	0.98
Final outcome	3.48	1	0.0017	0.97
Residuals	69728.55	35		

2.89.2) Levene's test

	Levene's test for homogeneity of variance	
	F test	p-value
Final outcome	F(1, 36)=0.79	0.38

2.89.3) Final outcome LS means graph

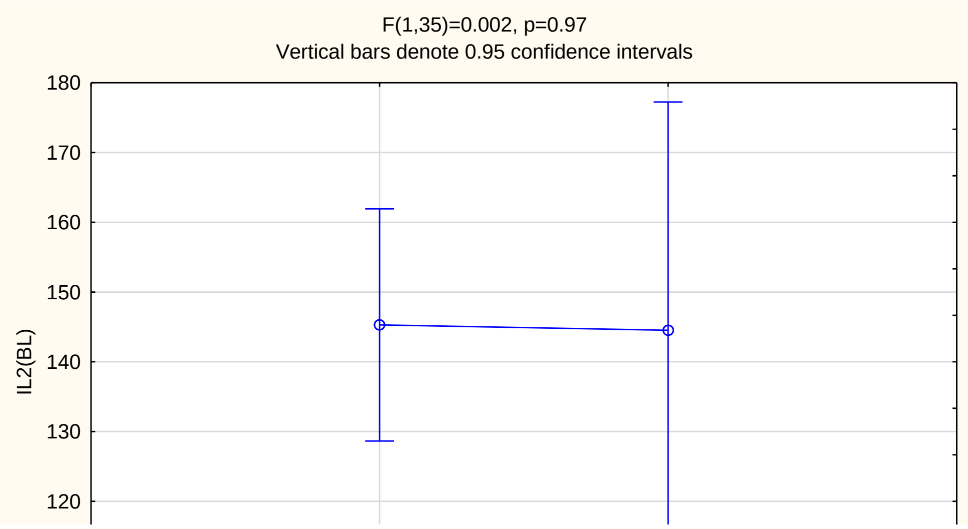

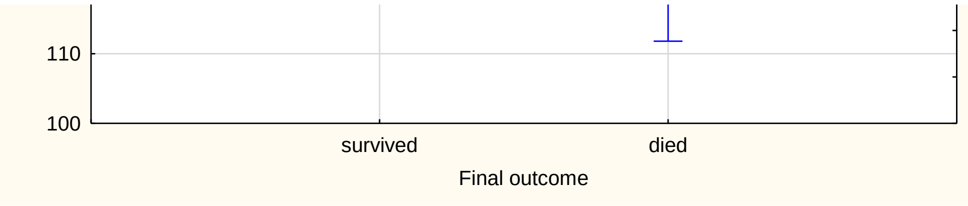

2.90) IL10(BL) with Age

2.90.1) ANOVA

	ANOVA type: III			
	Sum Sq	DF	F value	p value
(Intercept)	44.93	1	3.57	0.07
Age	0.02	1	0.001	0.97
Final outcome	56.11	1	4.458	0.04
Residuals	440.49	35		

2.90.2) Levene's test

	Levene's test for homogeneity of variance	
	F test	p-value
Final outcome	F(1, 36)=8.59	<0.01

2.90.3) Final outcome LS means graph

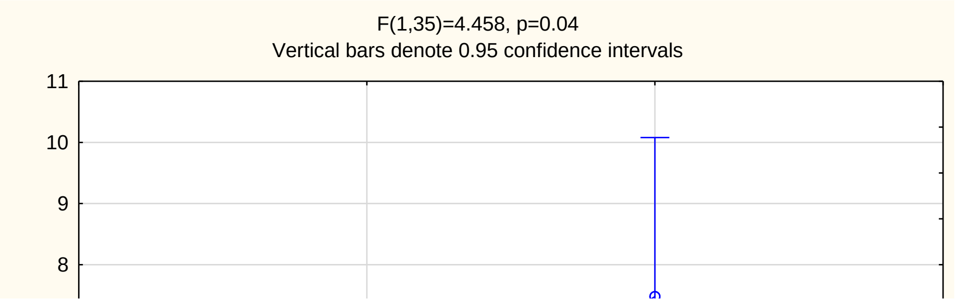

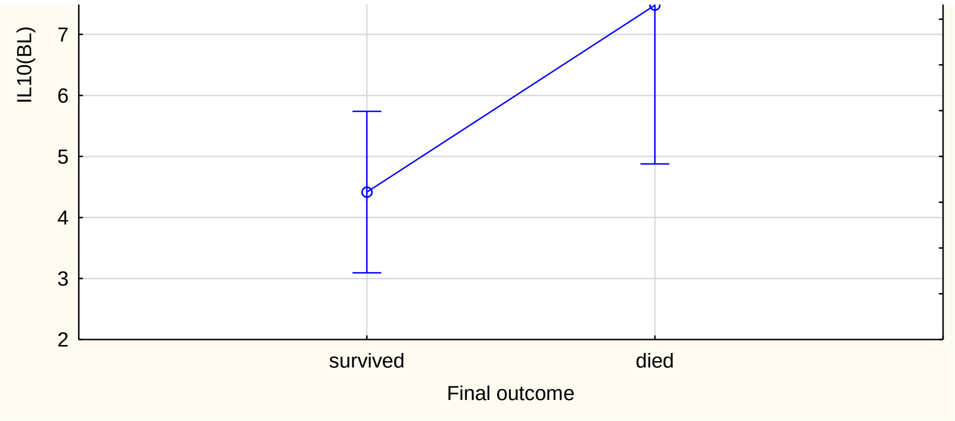

2.90.4) Welch tests

2.90.4.1) Final outcome adjusted means plot

2.91) TNFa(BL) with Age

2.91.1) ANOVA

	ANOVA type: III			
	Sum Sq	DF	F value	p value
(Intercept)	0.002	1	0.00009	0.99
Age	105.277	1	4.98006	0.03
Final outcome	3.683	1	0.1742	0.68
Residuals	739.888	35		

2.91.2) Levene's test

	Levene's test for homogeneity of variance	
	F test	p-value
Final outcome	F(1, 36)=0.58	0.45

2.91.3) Final outcome LS means graph

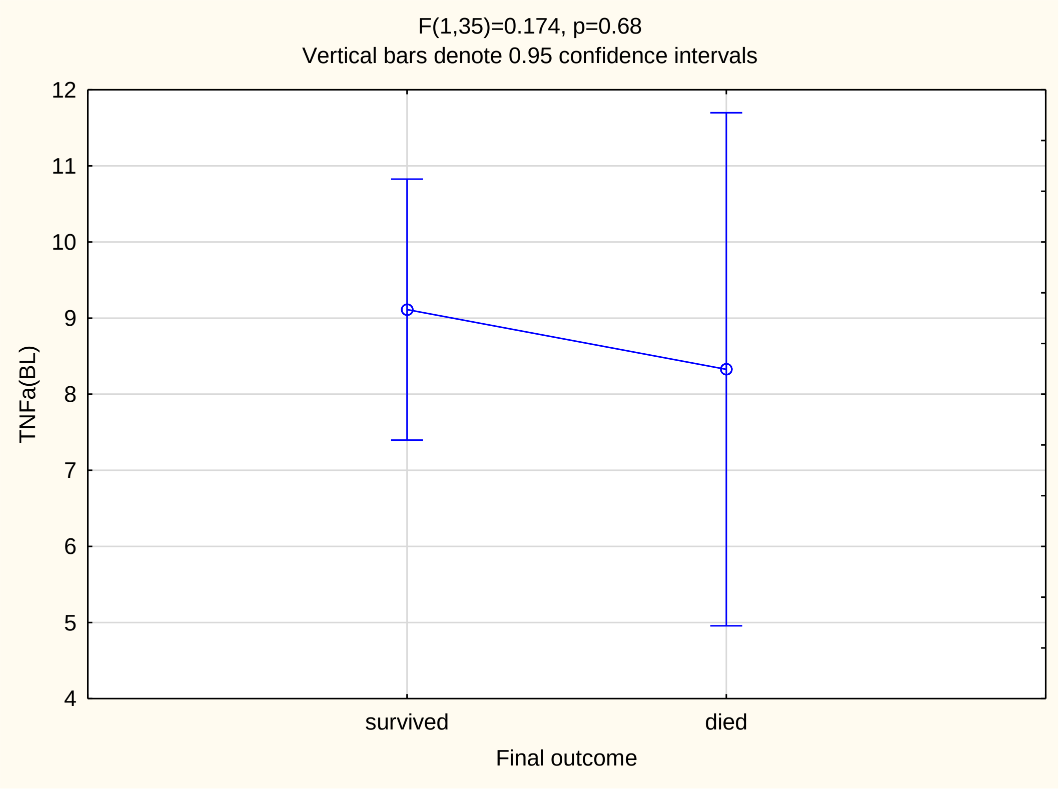

2.92) TNFa(AT)(winsorized) with Age

2.92.1) ANOVA

	ANOVA type: III			
	Sum Sq	DF	F value	p value
(Intercept)	0.43	1	4.1	0.05
Age	0.1	1	0.97	0.33
Final outcome	0.13	1	1.25	0.27
Residuals	3.7	35		

2.92.2) Levene's test

	Levene's test for homogeneity of variance	
	F test	p-value
Final outcome	F(1, 36)=4.89	0.03

2.92.3) Final outcome LS means graph

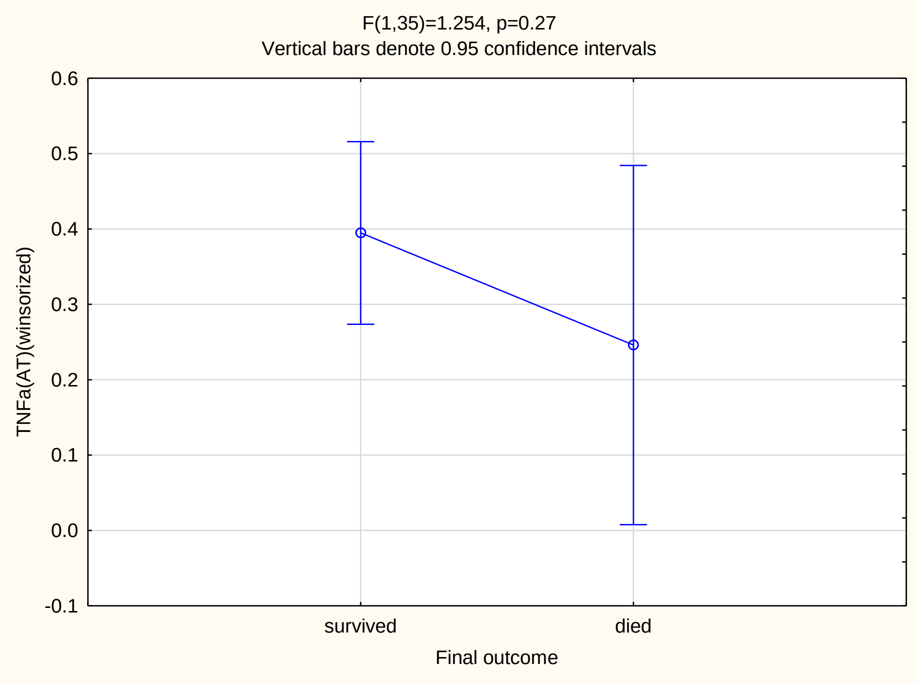

2.93) MCP1(AT)(winsorized) with Age

2.93.1) ANOVA

	ANOVA type: III			
	Sum Sq	DF	F value	p value

(Intercept)	395.45	1	2.63	0.11
Age	47.12	1	0.31	0.58
Final outcome	142.85	1	0.95	0.34
Residuals	5259.22	35		

2.93.2) Levene's test

	Levene's test for homogeneity of variance	
	F test	p-value
Final outcome	F(1, 36)=0.93	0.34

2.93.3) Final outcome LS means graph

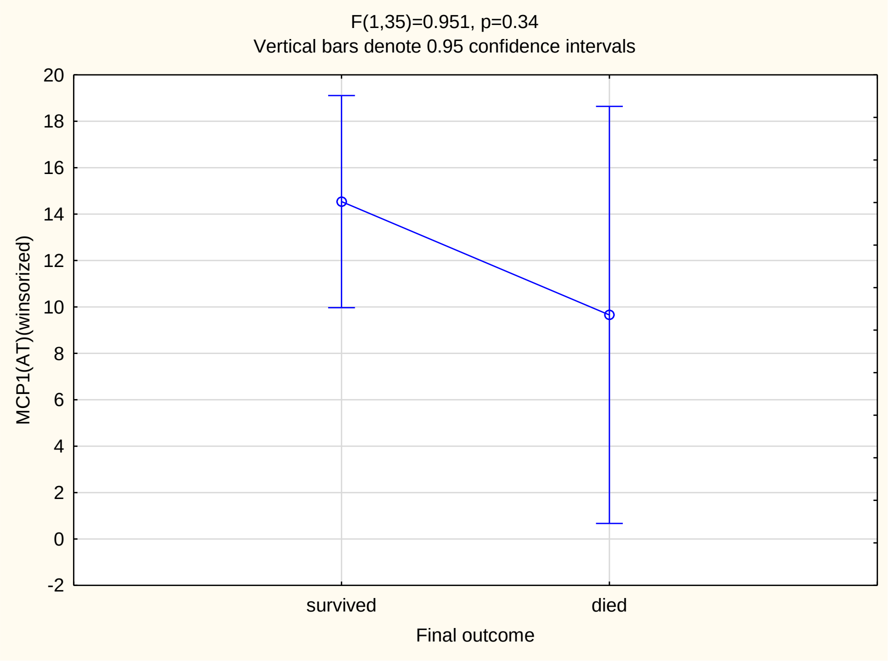

2.94) IL6(AT)(winsorized) with Age

2.94.1) ANOVA

	ANOVA type: III			
	Sum Sq	DF	F value	p value
(Intercept)	6.65	1	4.55	0.04
Age	1.82	1	1.24	0.27
Final outcome	1.15	1	0.79	0.38
Residuals	51.13	35		

2.94.2) Levene's test

	Levene's test for homogeneity of variance	
	F test	p-value
Final outcome	F(1, 36)=2.13	0.15

2.94.3) Final outcome LS means graph

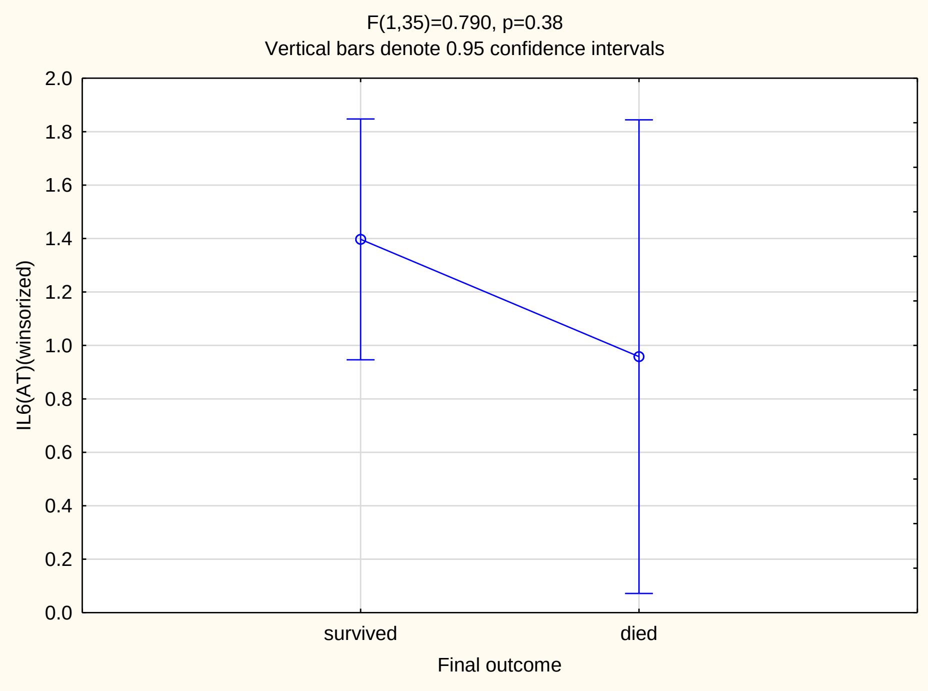

2.95) IL17 (AT)(winsorized) with Age

2.95.1) ANOVA

	ANOVA type: III			
	Sum Sq	DF	F value	p value
(Intercept)	0.96	1	4.27	0.05
Age	0.15	1	0.68	0.42
Final outcome	0.24	1	1.07	0.31
Residuals	7.9	35		

2.95.2) Levene's test

	Levene's test for homogeneity of variance	
	F test	p-value
Final outcome	F(1, 36)=1.61	0.21

2.95.3) Final outcome LS means graph

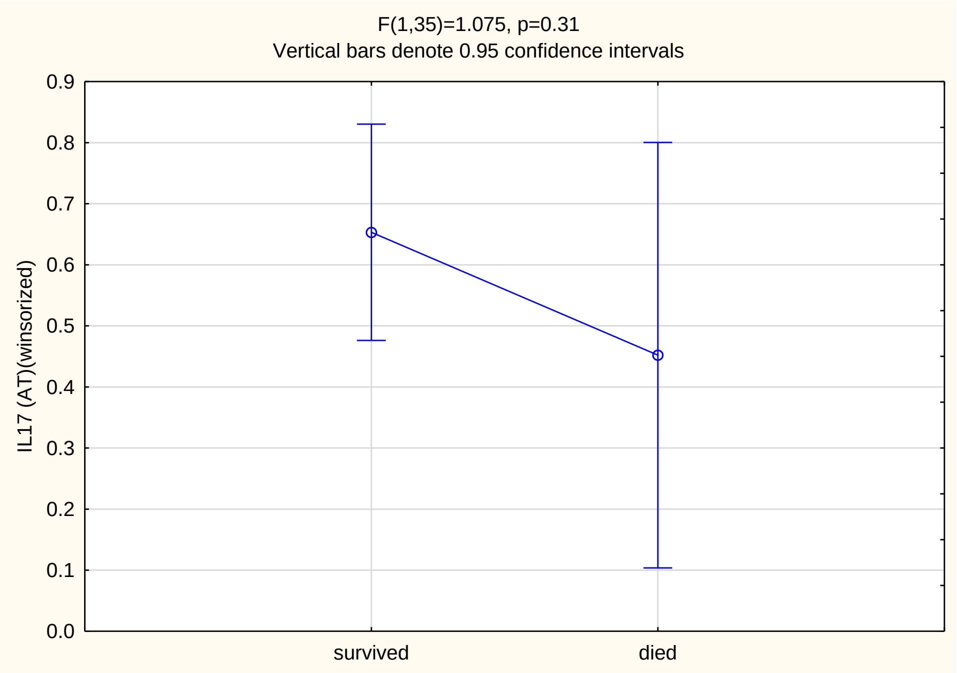

Final outcome

2.96) IL1b(BL)(winsorized) with Age

2.96.1) ANOVA

	ANOVA type: III			
	Sum Sq	DF	F value	p value
(Intercept)	42.7969	1	134.71	<0.01
Age	0.3733	1	1.175	0.29
Final outcome	0.0008	1	0.002	0.96
Residuals	11.1194	35		

2.96.2) Levene's test

	Levene's test for homogeneity of variance	
	F test	p-value
Final outcome	F(1, 36)=2.31	0.14

2.96.3) Final outcome LS means graph

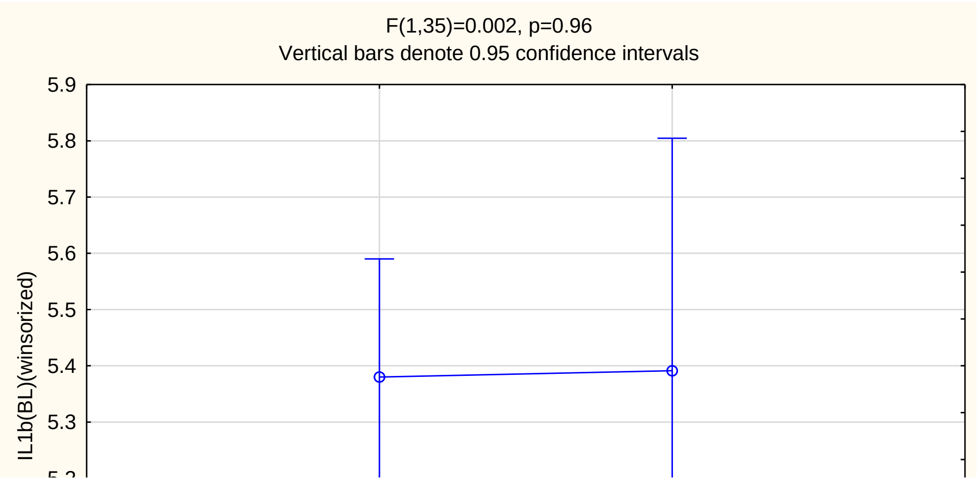

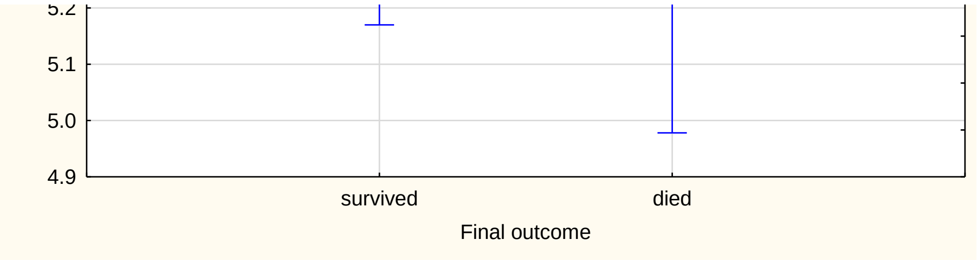

2.97) IL17(BL)(winsorized) with Age

2.97.1) ANOVA

	ANOVA type: III			
	Sum Sq	DF	F value	p value
(Intercept)	0.74	1	0.1	0.75
Age	5.16	1	0.69	0.41
Final outcome	0.8	1	0.11	0.74
Residuals	260.71	35		

2.97.2) Levene's test

	Levene's test for homogeneity of variance	
	F test	p-value
Final outcome	F(1, 36)=0.07	0.8

2.97.3) Final outcome LS means graph

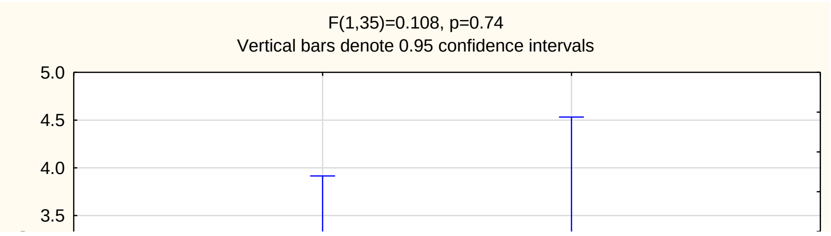

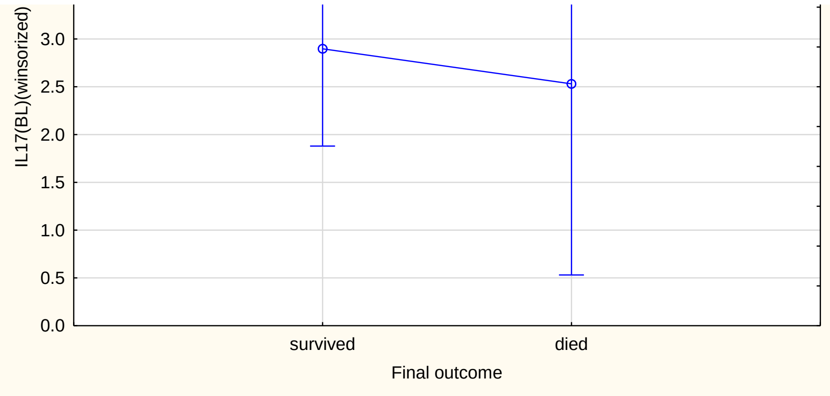

2.98) IL1b(Fl AT)(winsorized) with Age

2.98.1) ANOVA

	ANOVA type: III			
	Sum Sq	DF	F value	p value
(Intercept)	0.47	1	1.73	0.2
Age	0.03	1	0.12	0.73
Final outcome	0.75	1	2.73	0.11
Residuals	9.55	35		

2.98.2) Levene's test

	Levene's test for homogeneity of variance	
	F test	p-value
Final outcome	F(1, 36)=4.68	0.04

2.98.3) Final outcome LS means graph

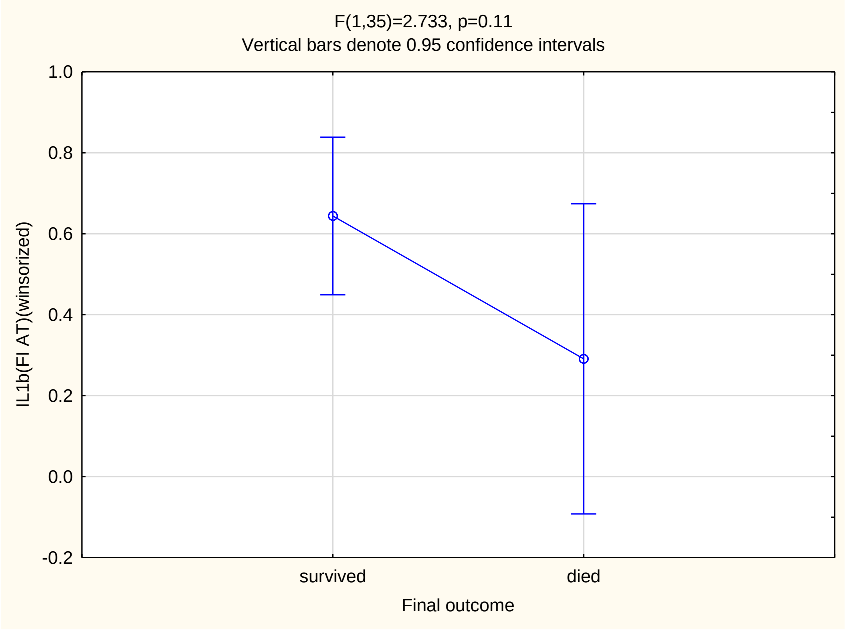
F(1,35)=2.733, p=0.11
Vertical bars denote 0.95 confidence intervals
1.0
0.8
0.6
0.4
0.2
0.0
-0.2
IL1b(FI AT)(winsorized)
survived
died
Final outcome

nsion

S
